# First-in-Class Selective Inhibitors of the Lysine Acetyltransferase KAT8

**DOI:** 10.1021/acs.jmedchem.2c01937

**Published:** 2023-05-08

**Authors:** Francesco Fiorentino, Sara Sementilli, Martina Menna, Federica Turrisi, Stefano Tomassi, Francesca Romana Pellegrini, Angela Iuzzolino, Francesca D’Acunzo, Alessandra Feoli, Hannah Wapenaar, Sophie Taraglio, Caterina Fraschetti, Donatella Del Bufalo, Gianluca Sbardella, Frank J. Dekker, Alessandro Paiardini, Daniela Trisciuoglio, Antonello Mai, Dante Rotili

**Affiliations:** † Department of Drug Chemistry and Technologies, Sapienza University of Rome, P.le A. Moro 5, Rome 00185, Italy; ‡ Institute of Molecular Biology and Pathology, National Research Council (CNR), Via degli Apuli 4, Rome 00185, Italy; § Department of Pharmacy, University of Naples “Federico II”, via Domenico Montesano 49, Naples 80131, Italy; ∥ Institute of Biological Systems (ISB), Italian National Research Council (CNR), Sezione Meccanismi di Reazione, c/o Department of Chemistry, Sapienza University of Rome, P. le A. Moro 5, Rome 00185, Italy; ⊥ Department of Pharmacy, University of Salerno, via Giovanni Paolo II 132, Fisciano (SA) 84084, Italy; # Department of Chemical and Pharmaceutical Biology, University of Groningen, Antonius Deusinglaan 1, Groningen 9713 AV, The Netherlands; ∇ Department of Biochemical Sciences, Sapienza University of Rome, P.le A. Moro 5, Rome 00185, Italy; ○ Preclinical Models and New Therapeutic Agents Unit, IRCCS-Regina Elena National Cancer Institute, Via Elio Chianesi 53, Rome 00144, Italy; ◆ Pasteur Institute, Cenci-Bolognetti Foundation, Sapienza University of Rome, P.le A. Moro 5, Rome 00185, Italy

## Abstract

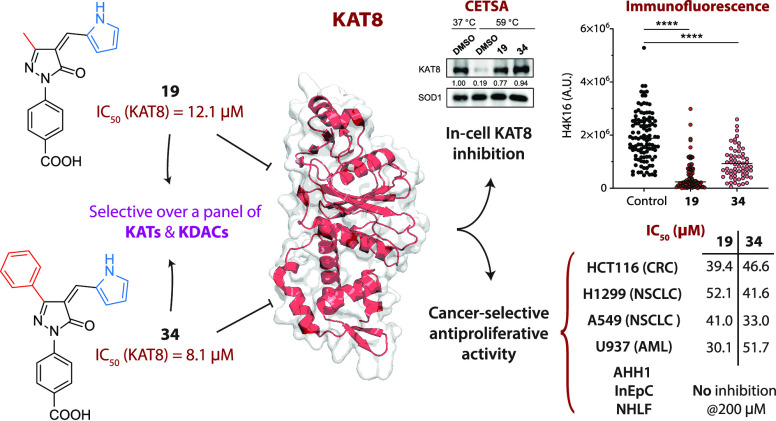

KAT8 is a lysine acetyltransferase primarily catalyzing the acetylation of Lys16 of histone H4 (H4K16). KAT8 dysregulation is linked to the development and metastatization of many cancer types, including non-small cell lung cancer (NSCLC) and acute myeloid leukemia (AML). Few KAT8 inhibitors have been reported so far, none of which displaying selective activity. Based on the KAT3B/KDAC inhibitor C646, we developed a series of *N*-phenyl-5-pyrazolone derivatives and identified compounds **19** and **34** as low-micromolar KAT8 inhibitors selective over a panel of KATs and KDACs. Western blot, immunofluorescence, and CETSA experiments demonstrated that both inhibitors selectively target KAT8 in cells. Moreover, **19** and **34** exhibited mid-micromolar antiproliferative activity in different cancer cell lines, including NSCLC and AML, without impacting the viability of nontransformed cells. Overall, these compounds are valuable tools for elucidating KAT8 biology, and their simple structures make them promising candidates for future optimization studies.

## 1 Introduction

Histone lysine acetylation is one of the key epigenetic post-translational modifications (PTMs) playing a crucial role in transcriptional and protein function regulation. The addition of an acetyl moiety to the ε-amino group of lysine residues is catalyzed by lysine acetyl transferases (KATs), which utilize acetyl coenzyme A (Ac-CoA) as a cosubstrate. Lysine acetylation neutralizes the positive charge on the ε-amino group, thereby profoundly influencing the biophysical properties of target proteins and affecting their interaction networks, subcellular localization, and sensitivity to degradation.^1^ The equilibrium between protein acetylation and deacetylation is tightly balanced, and its alteration is associated with a wide range of diseases, including neurodegeneration, inflammatory disorders, and cancer.^1−4^ On the basis of sequence homology and catalytic mechanism, KAT isoforms can be divided into three main families: the p300/CREB-binding proteins (p300/CBP) family, the GCN5-related *N*-Acetyltransferases (GNAT) family, and the MOZ, Ybf2, Sas2, and Tip60 (MYST) family.^5^

KAT8 (also called MOF or MYST1) is a MYST family member whose most studied catalytic activity consists of the acetylation on Lys16 of histone H4 (H4K16).^6^ KAT8 also acetylates transcription factors such as p53 and Nrf2, and it has recently been indicated to acetylate two additional histone H4 residues, namely, Lys5 (H4K5) and Lys8 (H4K8).^7^ KAT8 was initially identified in *Drosophila melanogaster* as part of the male-specific lethal (MSL) complex, which plays a key role in the dose compensation mechanism in males through H4K16 acetylation. Dose compensation is a regulatory mechanism that ensures males and females express the same amounts of X chromosome-associated gene products. In *Drosophila*, this is accomplished by doubling the transcription of X-linked genes in males, and the MSL complex has been demonstrated to be essential for this process.^7,8^ Although the dose compensation mechanism in mammals differs from that in flies, the MSL complex is also present in humans and comprises KAT8 as an acetylating subunit. In addition, KAT8 is part of the nonspecific lethal (NSL) complex, which acetylates Lys120 on p53, along with H4K16.^8^ Notably, the NSL complex was recently demonstrated to mediate acetylation of H4K5 and H4K8 at transcriptional start sites and is important for the expression of a subgroup of essential genes in human cells.^7^ Both MSL and NSL complexes regulate cell cycle progression^6^ and embryonic stem cell development.^9^ Furthermore, KAT8 is a key regulator of DNA damage response, autophagy, and apoptosis.^10^

**Figure 1:**
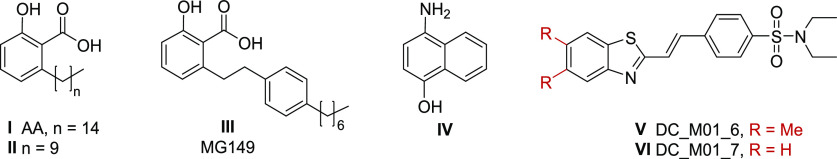
Currently reported KAT8 inhibitors.

Given its manifold functions in cellular homeostasis, KAT8 dysregulation is linked to the onset and progression of cancer.^1^ Early studies indicated that siRNA-mediated KAT8 silencing in HeLa cells led to downregulation of different oncogenes such as HOXA9, UCP2, KIAA0657, and HIP1 along with alteration of the cell cycle with a significant increase of cells in the G2/M stage.^6^ This report is in line with observations from different groups indicating an overexpression of *KAT8* in different cancer types in which it seems to play an oncogenic role. For instance, *KAT8* was found overexpressed in non-small cell lung cancer (NSCLC) where its activity promoted proliferation, migration, and adhesion. Indeed, *KAT8* knockdown with siRNA in NSCLC cell lines H1299 and A549 determined a moderate reduction of cell proliferation along with inhibition of cell migration and adhesion.^11^ This data is in line with another study indicating that *KAT8* knockdown in A549 cells decreased cell viability and induced cell cycle arrest at the G2/M phase.^12^ In addition, higher KAT8 levels were associated with a poor prognosis in patients affected by NSCLC.^13^ The two studies suggest that the oncogenic function of KAT8 is correlated with its acetylation activity toward H4K16^11^ and the transcription factor Nrf2.^13^ In breast cancer, KAT8-mediated acetylation was found to activate the transcriptional activator AIB1, an oncogene whose expression is increased in many cancer types.^14^ KAT8 is also overexpressed in oral tongue squamous cell carcinoma (OTSCC) where it plays a tumorigenic role by increasing the expression levels of the histone lysine methyltransferase EZH2.^15^ Furthermore, KAT8-mediated acetylation was shown to facilitate the oncogenic rearrangements of the mixed-lineage leukemia (*MLL*) gene. In a mouse model of *MLL-AF9*-driven leukemogenesis, *KAT8* gene deletion was associated with a decrease in acute myeloid leukemia (AML) cell proliferation.^16^*KAT8* is also overexpressed in endometrial carcinoma tissues, with its expression being associated with metastasis and shorter patient survival. Furthermore, high KAT8 levels promoted cell proliferation, migration, and invasiveness in endometrial carcinoma cell lines.^17^ Finally, KAT8 was demonstrated to possess a pivotal role in the vascular invasion of hepatocellular carcinoma (HCC).^18^

Altogether, these reports suggest that KAT8 is a promising target for the development of inhibitors that may act as therapeutics for different both solid and blood cancer types. Indeed, the KAT8-containing MSL complex has been recently demonstrated to be necessary for the maintenance of the proliferative potential of malignant cells. These findings were validated in various cancer cell lines (osteosarcoma, melanoma, fibrosarcoma, and breast cancer), as well as transformed dermal fibroblast-derived xenograft cancer models and patient-derived xenograft models of melanoma, gastric, and pancreatic cancer. Specifically, disruption of the MSL complex, with consequent loss of the associated mark, was shown to induce chromosomal instability of cancer cells, thereby leading to a progressive loss of their proliferative potential.^19^

Hence, the development of potent and selective KAT8 inhibitors (KAT8i) could greatly contribute to the discovery of new anti-cancer agents. In addition, selective KAT8i may be used as chemical probes to further clarify the function of this protein in both physiological and pathological settings. Nonetheless, only few KAT8i have been reported so far, and none of them display selective activity (Figure 1). To date, the reported KAT8i include anacardic acid (AA, **I**), which has *K_i_* and IC_50_ values of 64 and 43 μM,^20,21^ respectively, but also inhibits other KATs such as KAT3B (p300), KAT2B (KAT2B), and KAT5 (Tip60).^20−22^ Anacardic acid derivatives include compounds **II** (*K_i_* = 37 μM),^20^ and **III** (MG149, IC_50_ = 15–47 μM),^21^ which inhibit KAT8 in the mid-micromolar range, but display inhibitory activity also toward other KAT isoforms. Specifically, **II** also targets KAT3B and **III** is a KAT5 inhibitor. Another KAT8i is the fragment 4-amino-1-naphtol (**IV**, IC_50_ = 9.7 μM),^23^ which, however, inhibits KAT2B and KAT3B more efficiently.^23^ Recently, Zhang and co-workers reported a new series of KAT8i with the most potent compounds DC_M01_6 (**V**) and DC_M01_7 (**VI**) possessing IC_50_ values of 7.7 and 6 μM, respectively (Figure 1).^24^ Although these molecules could decrease H4K16 acetylation and inhibit the proliferation of HCT116 colon cancer cells, their selectivity over other KAT isoforms was not evaluated. Hence, further validation is necessary to imply a causal correlation between KAT8 inhibition and their observed phenotypic effects.

**Figure 2:**
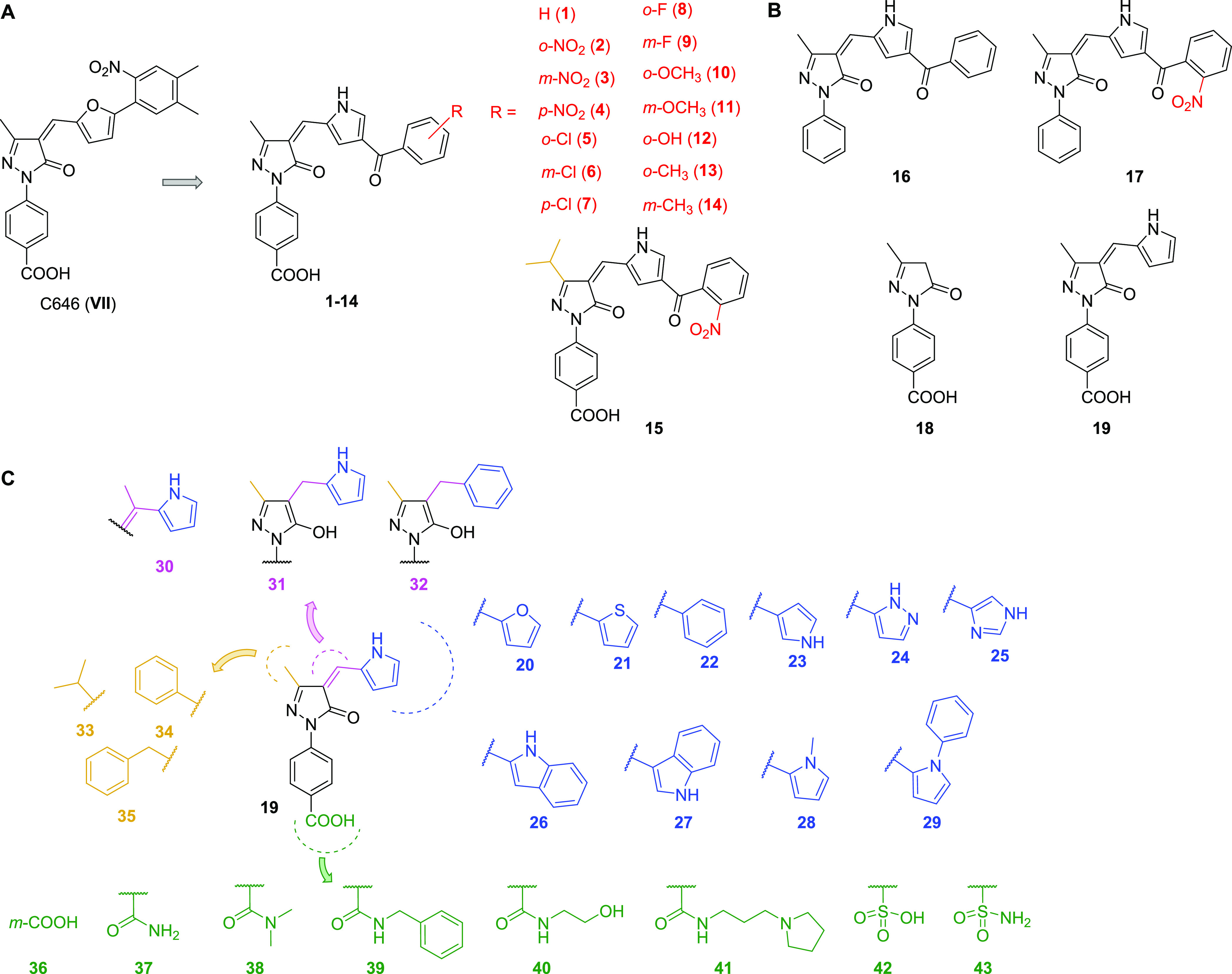
(A) Development of compounds **1**–**15** starting from the KAT3B/KDACs inhibitor C646 (**VII**). (B) Chemical structures of compounds **16**–**19** obtained via a molecular pruning approach. (C) Chemical structures of compounds **20**–**43** obtained by introducing various modifications on the structure of **19**.

Therefore, selective KAT8i have not been reported yet, although there is an urgent need for the development of novel chemical probes targeting this crucial enzyme, to further investigate the functions of KAT8 in both physiological and pathological contexts and hopefully to set the ground for new anticancer therapeutics. Following our long-standing research experience on KATi,^22,25−29^ we set out to develop novel KATi starting from the epigenetic modulator C646 (**VII**), an inhibitor of KAT3B (IC_50_ = 1.6 μM and *K_i_* = 0.4 μM),^30^ but also of lysine deacetylases (KDACs) with IC_50_ values of 7–25 μM for KDAC2, 3, 6, and 8.^31^ We designed a series of derivatives bearing a *N*-phenyl-5-pyrazolone core (compounds **1**–**43**, Figure 2), which were tested for their inhibitory potency against the three KAT enzymes KAT8, KAT3B, and KAT2B. The most promising inhibitors in terms of KAT8 inhibitory potency and isoform selectivity were then assessed in a panel of cancer cell lines, including the NSCLC cells H1299, A549, and NCI-H460, the MCF-7 breast cancer cell line, the AML cell lines U937 and THP-1, the colorectal carcinoma (CRC) cell lines HT29 and HCT116, and the HeLa cervical cancer cells.

## 2 Results and Discussion

### 2.1 Chemistry

**Scheme 1:**
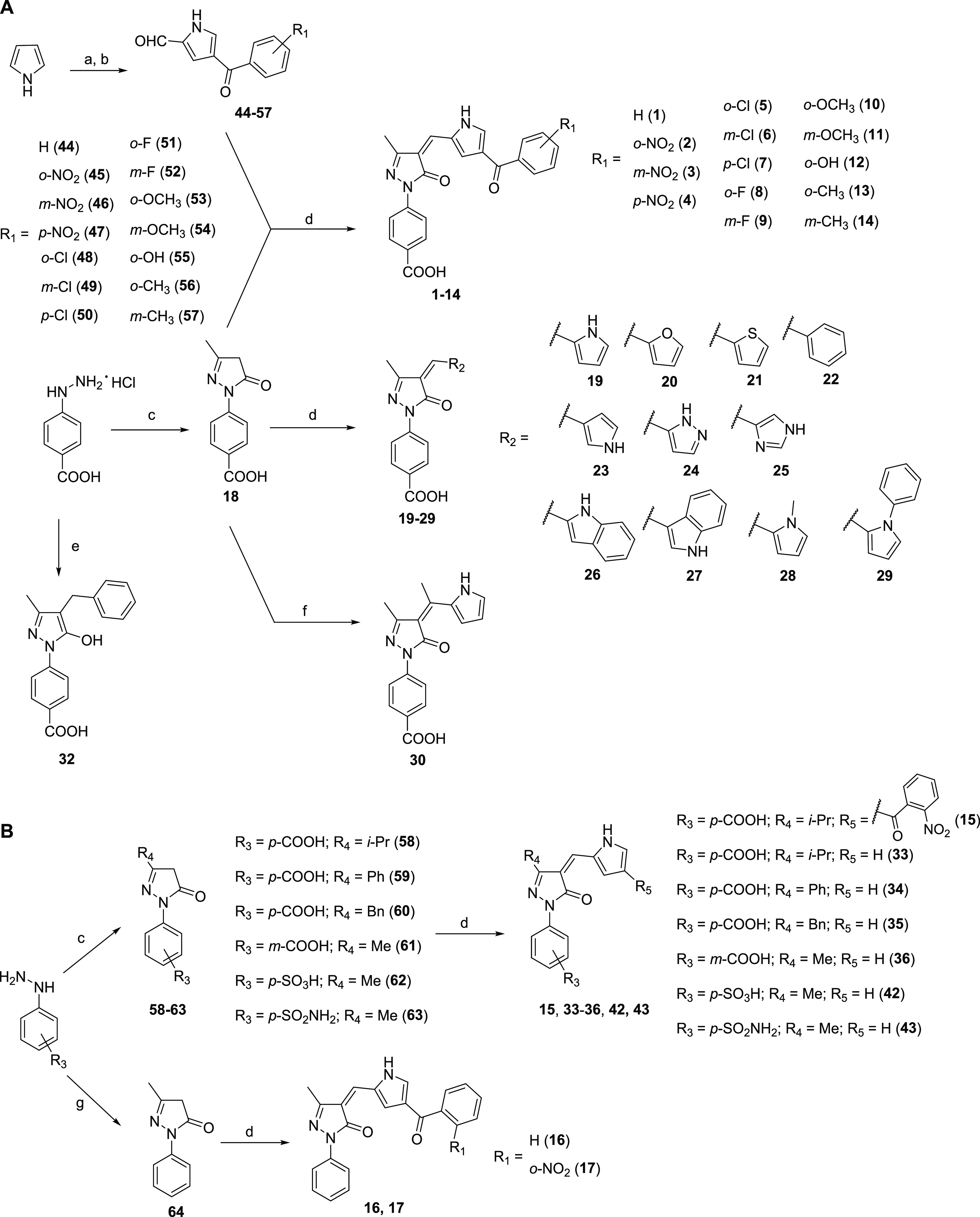
Synthesis of Compounds **1**–**30**, **32**–**36**, **42**, and **43** Reagents and conditions: (a) oxalyl chloride, dry DMF, dry DCE, 0 °C to rt; (b) appropriate benzoyl chloride, dry aluminum trichloride, rt; (c) appropriate β-ketoester, glacial acetic acid, reflux; (d) appropriate aldehyde, diethylamine, dry ethanol, 50 °C (rt for compounds **20**–**22**), N_2_; (e) ethyl 2-benzylacetoacetate, glacial acetic acid, reflux; (f) 2-acetylpyrrole, dry ethanol, 50 °C, N_2_; (g) ethyl acetoacetate, microwave, 120 °C.

The 4-benzoyl-1*H*-pyrrole-2-carbaldehyde intermediates **44**,^32^**45**, **46**, **47**,^33^**48**,^34^**49**, **50**, **51**,^35^**52**,^35^ and **53**–**57** were prepared via one-pot Vilsmeyer-Haack and Friedel-Crafts sequential reactions (Scheme 1A). The Vilsmeyer reagent was initially formed through the addition of oxalyl chloride to a solution of dry DMF in dry DCE, followed by the addition of pyrrole to yield the iminium intermediate. Dry aluminum trichloride and the appropriate benzoyl chloride were then added to obtain the intermediates **44**–**57**. The synthesis of the final compounds **1**–**14** and **19**–**29** started from the commercially available 4-hydrazinobenzoic acid hydrochloride, which underwent condensation with ethyl acetoacetate in glacial acetic acid under reflux conditions to yield the final compound **18**, as previously described.^30^ Compound **18** was converted into the final derivatives **1**–**14** via Knoevenagel condensation with aldehydes **44**–**57** in dry ethanol at 50 °C under a nitrogen atmosphere using diethylamine as a base (Scheme 1A). Compounds **19**, **20**,^36^**21**–**26**, **27**,^37^**28**, and **29** were obtained through the same reaction using the appropriate commercially available (hetero)aromatic aldehydes. The final compound **30** was obtained via Knoevenagel condensation of **18** with commercially available 2-acetylpyrrole under the same conditions as above. 4-Hydrazinobenzoic acid hydrochloride was also employed to prepare the final compound **32** following a condensation reaction with ethyl 2-benzylacetoacetate in glacial acetic acid under reflux conditions. 5-Pyrazolone intermediates **58**, **59**,^38^**60**, **61**,^30,39^**62**,^30^ and **63**(30) were synthesized from commercially available aryl hydrazines by condensation with the appropriate commercially available β-ketoester in glacial acetic acid under reflux conditions (Scheme 1B). Differently, intermediate **64** was obtained via condensation of phenylhydrazine and commercial ethyl acetoacetate under microwave irradiation at 120 °C, as previously reported.^40^ The final compounds **15**–**17**, **33**–**36**, **42**, and **43** were obtained through Knoevenagel condensation by treating the proper *N*-phenyl-5-pyrazolone intermediates (**58**–**63** for compounds **15**, **33**–**36**, **42**, and **43**; **64** for compounds **16**, **17**) with the appropriate aldehyde in dry ethanol at 50 °C under a nitrogen atmosphere using diethylamine as a base (Scheme 1B).

**Scheme 2:**
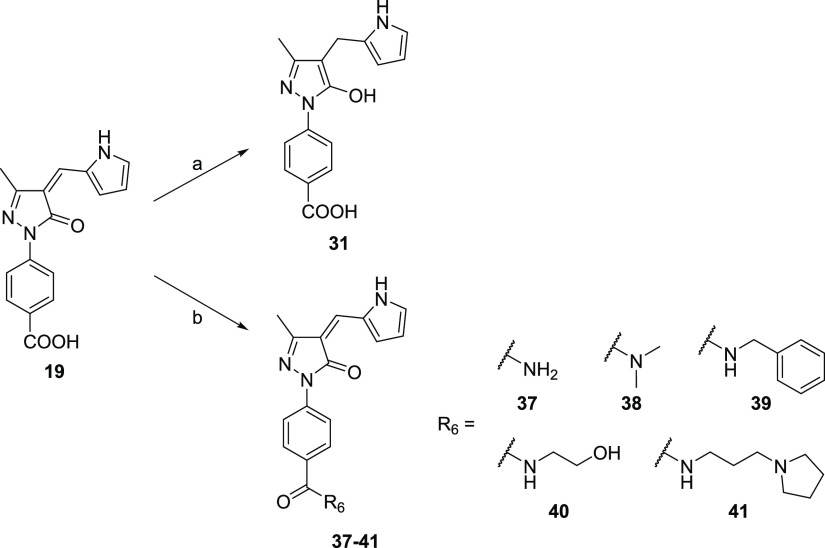
Synthesis of Compounds **31** and **37**–**41** Reagents and conditions: (a) sodium borohydride, dry THF/dry methanol 2:1, 0 °C to rt; (b) ammonia or an appropriate primary/secondary amine, triethylamine, PyBOP, dry DMF, rt, N_2_.

The final compound **31** was obtained as the 5-hydroxypyrazole prevalent tautomer from compound **19** by reducing the exocyclic double bond with sodium borohydride in a dry tetrahydrofuran/dry methanol mixture at 0 °C to rt (Scheme 2). The final compounds **37**–**41** were obtained from **19** via amide bond formation with ammonia (**37**) or the appropriate commercially available amines (**38**–**41**) at rt under a nitrogen atmosphere in dry DMF using benzotriazol-1-yloxytripyrrolidinophosphonium hexafluorophosphate (PyBOP) as a coupling reagent and triethylamine as a base (Scheme 2).

Each compound containing the exocyclic double bond was obtained as a single geometric isomer, except for compound **20**, obtained as a 3:1 (*Z*/*E*) mixture, in line with a previous report.^36^ We assigned the geometry of the double bond for each compound to *Z* based on NMR experiments, which are in line with earlier studies executed on numerous 4-arylidene *N*-phenyl-3-substituted-5-pyrazolones indicating the *Z* geometry as the favored one for this type of compounds.^30,36,37,39,41^

Chemical–physical data and elemental analyses for all newly synthesized final compounds **1**–**17** and **19**–**43** are reported in Tables S1 and S2 (Supporting Information), respectively. High-performance liquid chromatography (HPLC) traces for the final compounds tested in cells (**19**, **34**, and **39**) are reported in Figures S1–S3 (Supporting Information).

### 2.2 KAT8, KAT3B, and KAT2B Inhibition Assays and Structure–Activity Relationships

We initially prepared derivatives **1**–**15** bearing a general C646-like structure, in which the furan ring was replaced by a pyrrole and a carbonyl spacer was introduced between the pyrrole and aryl group (Figure 2A). Starting from the unsubstituted derivative **1**, we introduced different electron-withdrawing and electron-donating substitutions on the benzoyl moiety: *ortho*, *meta*, and *para* nitro (**2**, **3**, and **4**, respectively), *ortho*, *meta*, and *para* chloro (**5**, **6**, and **7**, respectively), *ortho* (**8**) and *meta* (**9**) fluoro, *ortho* (**10**) and *meta* (**11**) methoxy, *ortho* hydroxy (**12**), and *ortho* (**13**) and *meta* (**14**) methyl derivatives. Moreover, we prepared compound **15**, an analogue of **2** bearing an isopropyl moiety in place of the methyl group at the C3 position of the *N*-phenyl-5-pyrazolone ring.

These compounds were first tested to determine their inhibitory activity toward KAT8 (MYST family) and then against KAT3B and KAT2B, representative members of the other two main KAT families (p300/CBP and GNAT, respectively),^1^ to assess their isoform selectivity. Each assay was performed in triplicate with a 2-fold serial dilution starting at 200 μM concentration, yielding the relevant IC_50_ values. AA (**I**), MG149 (**III**), and C646 (**VII**) were also tested for comparison purposes (Table 1). Among the assayed molecules, compound **1**, bearing no substitution on the benzoyl moiety, along with the *ortho*-substituted derivatives **2** (*o*-NO_2_), **5** (*o*-Cl), and **13** (*o*-CH_3_) exhibited weak KAT8 inhibition, although they displayed 7- to 17-fold higher KAT3B inhibition, while none of them inhibited KAT2B. This data indicates that, although we could achieve KAT8 inhibition by applying some modifications to the C646 core (e. g., replacement of the furan ring by a pyrrole ring and introduction of a carbonyl spacer), the overall structure was still too similar to the parent compound to lose its preferential inhibition of KAT3B.

**Table 1:**
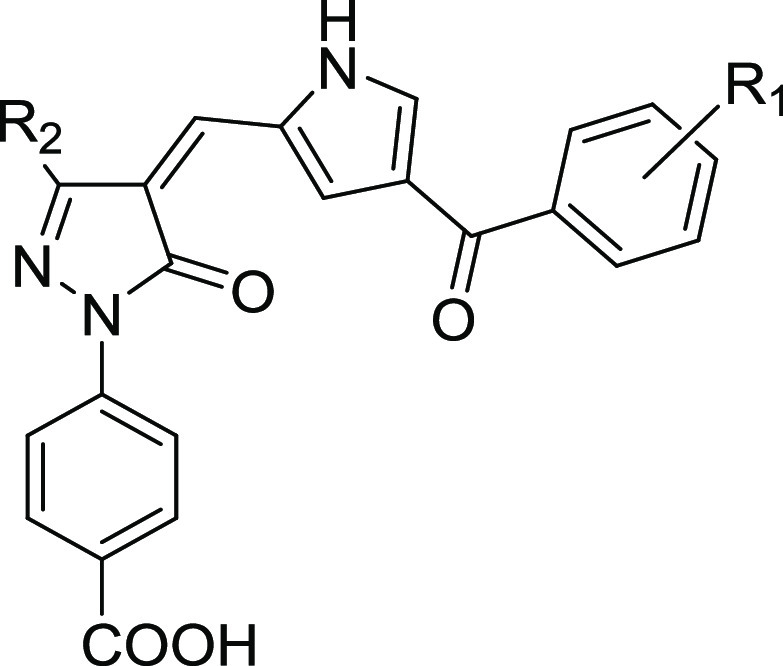
Inhibitory Activity of Compounds **1**–**15** against KAT8, KAT3B, and KAT2B^a^

			IC_50_^b^ (μM)		
compd	R_1_	R_2_	KAT8 (5 nM)	KAT3B (1 nM)	KAT2B (25 nM)
**1** (MC3983)	H	CH_3_	90.5 ± 11.3	13.6 ± 1.0	NI^c^
**2** (MC3637)	*o*-NO_2_	CH_3_	86.2 ± 10.0	9.1 ± 0.6	NI
**3** (MC4039)	*m*-NO_2_	CH_3_	>150	NT^d^	NT
**4** (MC4052)	*p*-NO_2_	CH_3_	NI	NT	NT
**5** (MC3987)	*o*-Cl	CH_3_	88.9 ± 12.1	5.3 ± 0.3	NI
**6** (MC4008)	*m*-Cl	CH_3_	>150	NT	NT
**7** (MC4049)	*p*-Cl	CH_3_	NI	NT	NT
**8** (MC3996)	*o*-F	CH_3_	>150	NT	NT
**9** (MC4023)	*m*-F	CH_3_	NI	NT	NT
**10** (MC3991)	*o*-OCH_3_	CH_3_	>150	NT	NT
**11** (MC4028)	*m*-OCH_3_	CH_3_	NI	NT	NT
**12** (MC4006)	*o*-OH	CH_3_	NI	NT	NT
**13** (MC3989)	*o*-CH_3_	CH_3_	95.0 ± 10.2	8.9 ± 0.6	NI
**14** (MC4020)	*m*-CH_3_	CH_3_	NI	NT	NT
**15** (MC4248)	*o*-NO_2_	*i*-Pr	>150	18.5 ± 1.4	NI
**I** (AA)			30.2 ± 2.1	22.9 ± 1.3	39.7 ± 1.1
**III** (MG149)			20.3 ± 1.3	22.3 ± 1.1	NI
**VII** (C646)			NI	0.191 ± 0.009	NI

a Values are means ± standard deviation (SD) of three independent experiments.

b Half maximal inhibitory concentration: dose required to inhibit the enzymatic activity by 50%. Enzyme concentrations used in the assays are specified in brackets in the table header.

c NI, no inhibition at 200 μM.

d NT, not tested.

Hence, with the aim of increasing KAT8 potency and selectivity and to gain SAR, we carried out a molecular pruning approach (Figure 2B). To this end, we removed the carboxylic group from compounds **1** and **2**, yielding compounds **16** and **17**, respectively. Alternatively, we removed either the whole 3-benzoyl-1*H*-pyrrole or only benzoyl moiety, leading to compounds **18** and **19**, respectively (Figure 2B). Remarkably, while compounds **16**–**18** were all inactive against KAT8, **19** exhibited a promising KAT8 inhibition (IC_50_ = 12.1 μM) coupled with a significant selectivity over KAT3B and KAT2B (no inhibition at 200 μM) (Table 2). These results indicate that the carboxylic group is important for compound activity (see compounds **16** and **17**) and a heteroaromatic group at the C4 position of the 5-pyrazolone ring is necessary for KAT8 inhibition (see compound **18**), while removal of the benzoyl portion shifts the selectivity from KAT3B to KAT8 (compare **1** in Table 1 with **19** in Table 2).

**Table 2:**
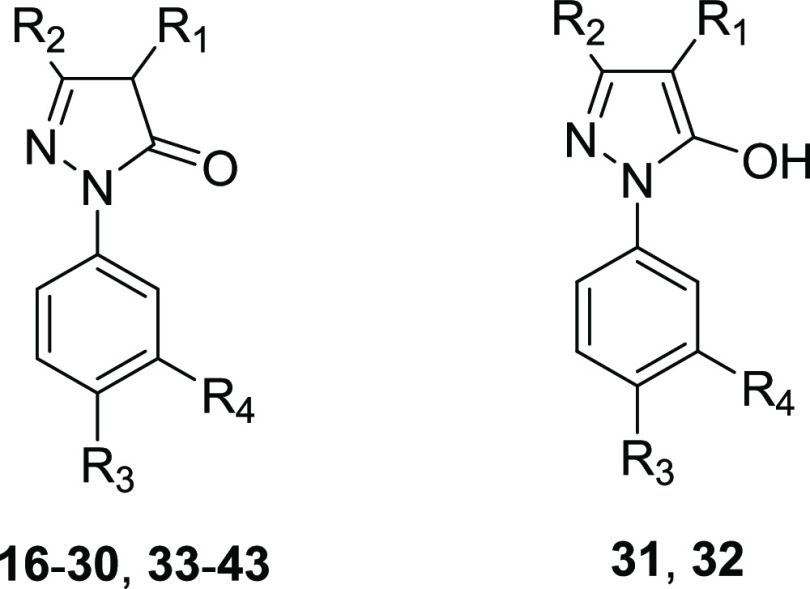

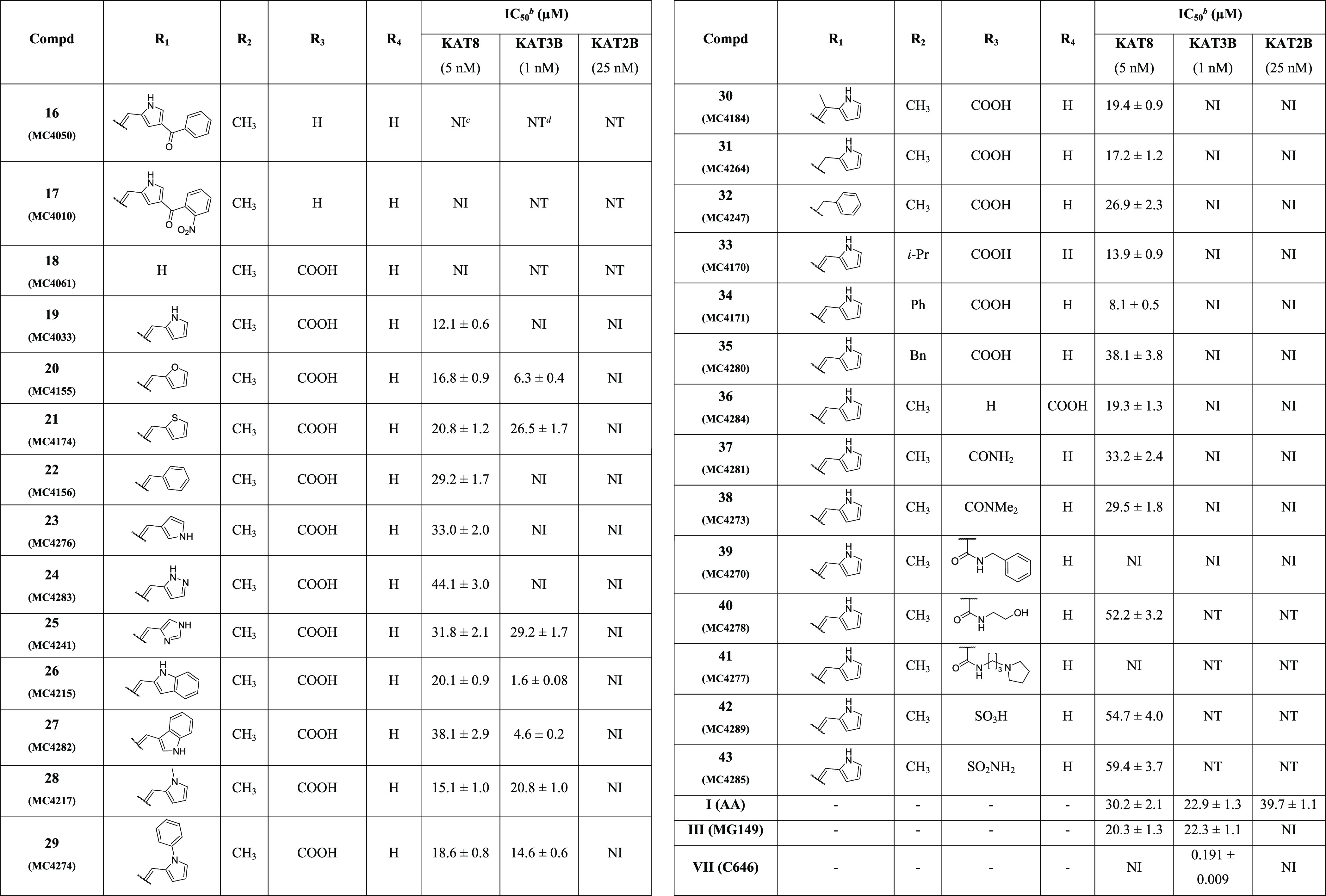
Inhibitory Activity of Compounds **16**–**43** against KAT8, KAT3B, and KAT2B^a^

a Values are means ± standard deviation (SD) of three independent experiments.

b Half maximal inhibitory concentration: dose required to inhibit the enzymatic activity by 50%. Enzyme concentrations used in the assays are specified in brackets in the table header.

c NI, no inhibition at 200 μM.

d NT, not tested.

The encouraging results obtained with compound **19** prompted us to develop new derivatives to investigate the SAR of this compound series and yield more potent and selective KAT8i (Figure 2C). To explore the importance of the pyrrole ring, we prepared compounds **20**–**27**, in which the pyrrole was replaced by different (hetero)aromatic rings, and compounds **28** and **29**, in which the pyrrole nitrogen presented a methyl or phenyl substitution, respectively. We explored the importance of the exocyclic double bond by either replacing the (1*H*-pyrrol-2-yl)methylidene with the 1-(1*H*-pyrrol-2-yl)ethylidene group (**30**), reducing it to the (1*H*-pyrrol-2-yl)methylene portion (**31**), or combining the double bond reduction with the replacement of pyrrole with a phenyl ring (**32**). Moreover, we investigated the influence of bulkier substitutions on the C3 of the 5-pyrazolone ring by replacing the methyl group with isopropyl (**33**), phenyl (**34**), and benzyl (**35**) moieties. Finally, we applied various modifications to the benzoic acid portion, by either changing the position of the carboxylic group from *para* to *meta* (**36**) or replacing it with various amides (**37**–**41**), sulfonic acid (**42**), or sulfonamide (**43**) functions.

These compounds were tested in the same inhibition assays (first against KAT8, then KAT3B and KAT2B for selectivity evaluation purposes) as above. Results obtained from these tests indicated that both adding a substitution to the pyrrole nitrogen of **19** and replacing the pyrrole ring with other (hetero)aryl moieties causes the loss of KAT8 selectivity, with the only exceptions being **22**, possessing a phenyl ring in place of the pyrrole, **23**, the 3′-substituted pyrrole regioisomer of **19**, and **24**, bearing a 5′-substituted pyrazole instead of the pyrrole ring (Table 2). These compounds were selective over KAT3B and KAT2B, although they displayed 2.4- to 3.7-fold lower KAT8 inhibitory potency compared to **19**. Similarly, addition of a methyl substituent on the exocyclic double bond (**30**) or its reduction (**31** and **32**, both were observed in ^1^H-NMR experiments in DMSO as the 5-hydroxypyrazole prevalent tautomeric forms)^42,43^ slightly decreased KAT8 inhibitory potency, while keeping the selectivity over KAT3B and KAT2B. Hence, the presence of the exocyclic double bond and, consequently, the α,β-unsaturated system is not crucial for KAT8 inhibition. Notably, substituting the methyl group on the C3 position of the 5-pyrazolone ring with the isopropyl (**33**), one maintained the KAT8 inhibitory activity and selectivity compared to **19**, and even increased potency in case of phenyl (**34**) substitution, yielding an IC_50_ value of 8.2 μM. In contrast, the introduction of a benzyl group at the C3 position of the 5-pyrazolone ring provided compound **35** that retained the selectivity over KAT3B and KAT2B but decreased the potency toward KAT8 (Table 2). Finally, moving the carboxyl group from *para* to *meta* position led to a slight decrease in KAT8 inhibitory potency (**36**), while replacing it with amides (**37**–**41**), sulfonic acid (**42**) or suldfonamide (**43**) groups led to a more significant potency decrease, especially in the cases of derivatives **39** and **41** (Table 2).

Overall, these results indicate that the (hetero)aromatic moiety bound to the C4 position of the *N*-phenyl-5-pyrazolone core is determinant for compound selectivity and the unsubstituted pyrrole is the preferred ring, leading to the highest KAT8 inhibition and selectivity over KAT3B and KAT2B. Moreover, the presence of the benzoic acid is essential for compound activity, with the *para* position being preferred over the *meta* one, and the introduction of a phenyl substitution at the C3 position of the 5-pyrazolone core increases KAT8 inhibition, likely through additional interactions, which are abolished when adding a methylene spacer.

Overall, for the first time, we managed to obtain KAT8i with a proved *in vitro* selectivity over other KAT isoforms, with compounds **19**, **31**, **33**, and **34** being the most potent, displaying IC_50_ values between 8 and 17 μM.

### 2.3 Effects of Compounds **19** and **34** on the Activity of a Panel of KATs and KDACs

To further explore the selectivity profile of this compound class, we decided to test the two most potent KAT8 inhibitors (**19** and **34**) against a panel of KATs (KAT2A and the MYST family members KAT5, KAT6A, KAT6B, and KAT7). AA was used as a positive control for KAT2A, while C646 was used as a positive control for the other KATs. Both compounds did not inhibit KAT2A at 200 μM, while they exerted minimal inhibition to the MYST family members at the same concentration (Table 3), with compound **34** exerting the maximum 20.9% inhibition against KAT6A at 200 μM. Moreover, since the synthesized compounds share the same *N*-phenyl-3-methyl-5-pyrazolone core as C646 (**VII**), we decided to test the KAT8i **19** and **34** against a panel of KDACs (KDAC1–3, 6, and 8) using the same assay we employed previously.^31^ C646 and SAHA were used as positive controls. Notably, in contrast to C646, none of the compounds was active against any of the assayed KDACs under the experimental conditions (Table S3).

**Table 3:** Inhibitory Activity of Compounds **19**, **34**, **I**, and **VII** against KAT2A, KAT5, KAT6A, KAT6B, and KAT7^a^

	% inhibition at 200 μM^b^				
compd	KAT2A (15 nM)	KAT5 (25 nM)	KAT6A (10 nM)	KAT6B (10 nM)	KAT7 (25 nM)
**19**	NI^c^	11.3 ± 0.3%	NI^*b*^	4.8 ± 1.6%	NI
**34**	NI	18.5 ± 1.3%	20.9 ± 0.4%	19.6 ± 1.0%	16.9 ± 0.7%
**I** (AA)	IC_50_ = 42.1 ± 2.3 μM	NT^d^	NT	NT	NT
**VII** (C646**)**	NT	IC_50_ = 18.3 ± 1.0 μM	IC_50_ = 15.0 ± 1.1 μM	IC_50_ = 5.6 ± 0.3 μM	IC_50_ = 8.0 ± 0.4 μM

a Values are means ± standard deviation (SD) of three independent experiments.

b IC_50_ values measured for control compounds AA and C646. Enzyme concentrations used in the assays are specified in brackets in the table header.

c NI, no inhibition.

d NT, not tested.

### 2.4 Surface Plasmon Resonance (SPR)-Based Binding Assay

**Figure 3:**
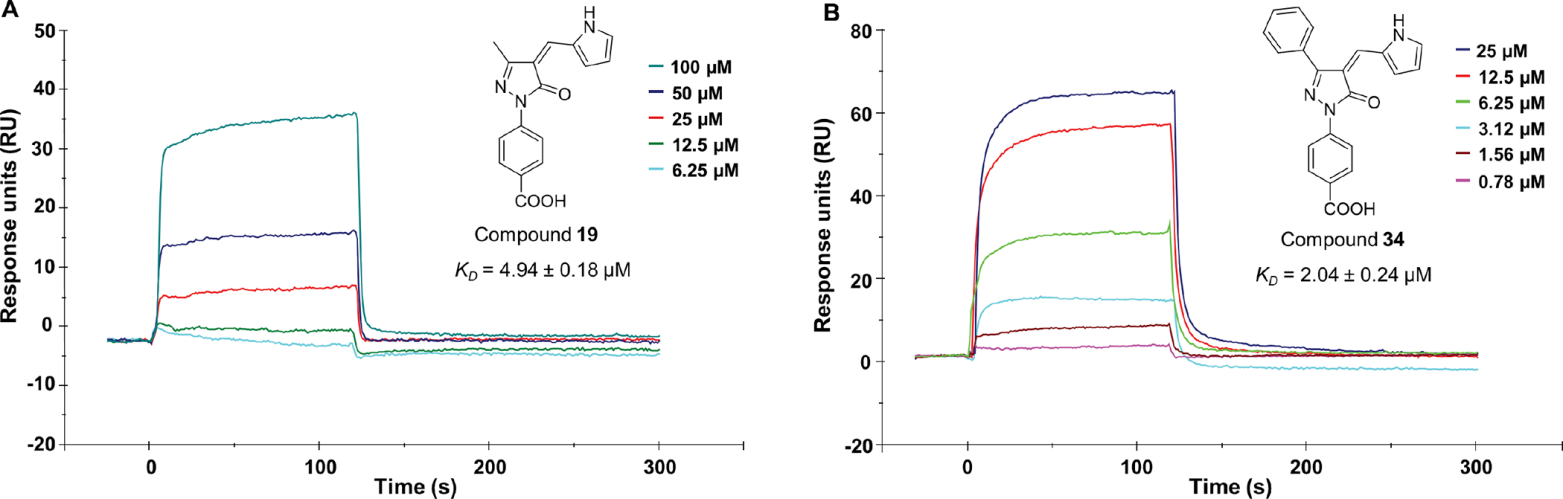
Sensorgrams obtained from the SPR interaction between immobilized KAT8 and compound **19** (A) or **34** (B). Each compound was injected at different concentrations with an association time of 120 s and a dissociation time of 200 s, with a flow rate of 30 μL/min.

To further validate the most potent and selective compounds **19** and **34** as KAT8i, we set out to perform a binding assay via SPR. This experiment enables the quantification of the direct interaction between KAT8 and any potential binder. In both cases, the interaction was dose-dependent, and the equilibrium dissociation constants (*K_D_*) were 4.94 ± 0.18 μM (**19**, Figure 3A) and 2.04 ± 0.24 μM (**34**, Figure 3B), consistent with the KAT8 inhibitory potency observed in the biochemical assay.

### 2.5 Compounds **19** and **34** Are Reversible KAT8 Inhibitors

Given the presence of an exomethylene vinyl moiety that may act as a Michael acceptor, we assessed whether the most potent compounds **19** and **34** could react with thiol-containing nucleophiles or act as covalent inhibitors. To this end, we performed HPLC in the same buffer of the enzymatic assay but with the addition of 2 mM (4 equivalents) of dithiothreitol (DTT), β-mercaptoethanol (BME), or reduced glutathione (GSH). The chosen concentration is in line with the physiological levels of GSH in cells, which are usually 1–2 mM.^44^ HPLC chromatography analysis at three different wavelengths (254, 286, and 395 nm) revealed no evidence of reaction between **19** or **34** and the tested nucleophiles after 1, 8, or 24 h of incubation at room temperature (Figure S4, Figure S5). To investigate the reversibility of KAT8 inhibition, we performed a preincubation assay by incubating **19** or **34** for 5, 10, 20, 30, 60, and 120 min with KAT8 before adding the H4 peptide and Ac-CoA substrates (final inhibitor concentration was 12.5 μM) and performing the same KAT8 activity inhibition assay as before (Figure S6A,B). In both cases, we did not observe time-dependent inhibition, thus suggesting that both compounds act as noncovalent inhibitors. We further confirmed these findings by performing a jump-dilution experiment. In this assay, after a 30 min exposure of 0.5 μM KAT8 to each inhibitor at a concentration equal to 10× their IC_50_ (121 μM for **19** and 81 μM for **34**), the mixtures were diluted 100-fold and KAT8 activity was measured after 10, 20, 30, 60, 120, and 240 min (Figure S6C). The assay indicated that KAT8 regains ∼90% of its activity after the inhibitor (**19** or **34**) dilution at any timepoint with its activity increasing with time as well as the no inhibitor (DMSO) control. Overall, these findings confirm that both **19** and **34** act as reversible inhibitors. The absence of thiol reactivity may be the result of the extensive conjugation of the polyaromatic systems present in both compounds.

### 2.6 Computational Analysis of Compound Binding to KAT8, KAT3B, and KAT2B

**Figure 4:**
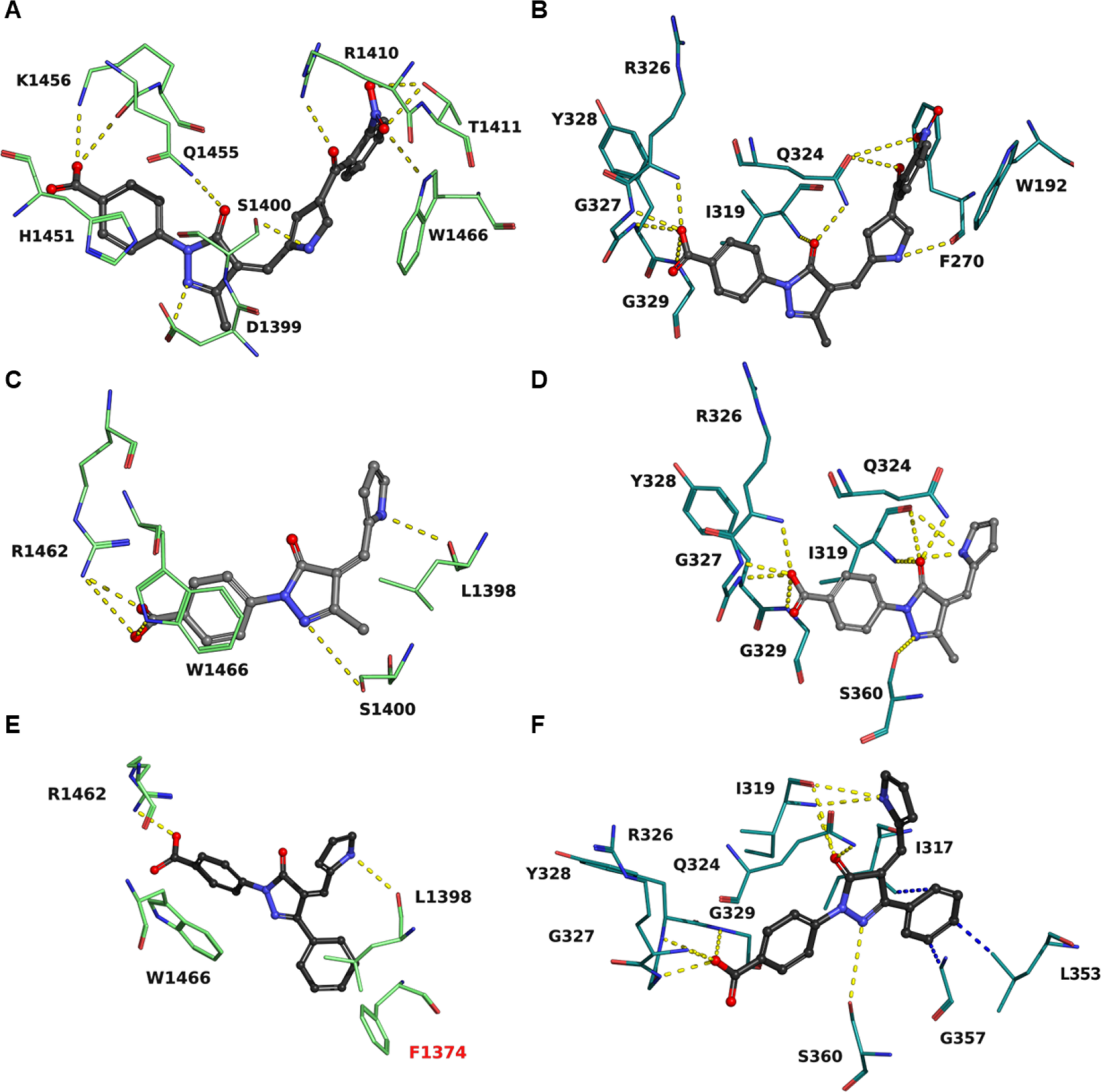
(A, B) Compound **2** (dark gray) docked in the active site of KAT3B (A) and KAT8 (B). (C, D) Compound **19** (gray) docked in the active site of KAT3B (C) and KAT8 (D). (E, F) Compound **34** (black) docked in the active site of KAT3B (E) and KAT8 (F). Hydrogen bonds are shown as yellow dashed lines. Hydrophobic interactions are shown as blue dashed lines. Residues of the biding pocket involved in the interactions are shown. Clashing residues are labeled in red.

To shed light on the binding mode and rationalize the selectivity of the novel KAT8i, we performed molecular docking and molecular dynamics (MD) experiments. We initially benchmarked the docking parameters for their ability to reproduce the conformation of several experimentally validated cocrystalized inhibitors or cosubstrates of KATs (Figure S7). Subsequently, we predicted the binding mode of the parent compound C646 to human KAT3B (PDB Code: 5KJ2)^45^ (Figure S8A) and compared it with the same pose in human KAT8 (PDB Code: 6PDB)^46^ (Figure S8B). The first series of inhibitors (**1**–**15**), which are structurally similar to C646 because it share a common structural skeleton made of a 4-(3-methyl-5-oxo-4*H*-pyrazol-1-yl)benzoic acid, exhibit a similar set of interactions with KAT3B compared to C646, as exemplified by compound **2** (compare Figure 4A with Figure S8A) that was used as representative of this series of molecules because it is one of the most active KAT3B/KAT8 inhibitors. Compared to C646, **2** has a pyrrole ring in place of the furan moiety, a 2-nitrophenyl group replacing the 2-nitro-4,5-dimethylphenyl group of C646, and an additional carbonyl between the pyrrole ring and 2-nitrobenzene. In KAT3B, both C646 and **2** engage in multiple hydrogen bonds and π-stacking interactions with key active site residues (compare Figure 4A with Figure S8A). The only differences concern the hydrogen bond interaction with Ser1400, which is mediated by the pyrrole NH group of **2** while in C646 by the carbonyl oxygen of the 5-pyrazolone ring, and with Arg1410, which involves the benzoyl carbonyl group of **2**, whereas in C646 the nitro group of the 2-nitro-4,5-dimethylbenzene moiety (Figure 4A and Figure S8A). These structural variations can explain the difference in the inhibitory potencies of the two molecules [IC_50_(KAT3B) = 0.191 μM for C646 vs IC_50_(KAT3B) = 9.1 μM for **2**]. C646 is not able to bind KAT8 because of the clash of its bulky 2-nitro-4,5-dimethylbenzene group with Phe270 inside the smaller cleft of KAT8 (Figure S8B). In fact, this cavity is narrower than that of KAT3B, due to the presence of bulky residues such as Phe270, Trp192, Gln324, and Pro321 (Figure S8B). Moreover, in contrast to what happens with KAT3B, the nitrogen atom at the 2-position of the 5-pyrazolone ring of C646 is not able to form any hydrogen bonds within the KAT8 active site (compare Figure S8A with Figure S8B). Conversely, compared to C646, compound **2** forms an additional hydrogen bond with the carbonyl group of the Phe270 backbone of KAT8, thanks to the presence of the hydrogen-bond donor NH group of the pyrrole ring (Figure 4B). Moreover, the additional degree of freedom provided by the carbonyl group between the pyrrole ring and 2-nitrophenyl moiety, which is missing in C646, confers the necessary flexibility to prevent the formation of clashes between the nitro group and Phe270 (Figure S8C). These structural differences may explain the higher KAT8 inhibition of the **1**–**15** compound series compared to C646, although the size of the KAT8 active site pocket is barely sufficient to accommodate their bulky benzoyl groups at the pyrrole C4 position (see compound **2** in Figure 4B).

The compound series based on **19** exhibit a small common structural skeleton consisting of a 4-(5-oxo-4*H*-pyrazol-1-yl)benzoic acid, while lacking the benzoyl moiety. In KAT3B, the structural scaffold shared by **19**- and **2**-like inhibitors mediates similar interactions, but the lack of a fourth aromatic ring in **19** weakens its binding affinity due to the absence of many hydrogen bonds, thus impairing its inhibitory activity (Figure 4C). Conversely, **19** fits perfectly into the KAT8 active site and engages in several hydrogen bonds with many residues (Figure 4D). Specifically, the carbonyl portion of the 5-pyrazolone ring and the NH group of the pyrrole moiety form an intricate network of hydrogen bonds with the side chain of Gln324 and with the backbone nitrogen and oxygen of Ile319. Moreover, the nitrogen at the 2-position of the 5-pyrazolone engages in a hydrogen bond with the side chain of Ser360 and the carboxylic group forms multiple hydrogen bonds with the backbone amide nitrogen atoms of Arg326, Gly327, Tyr328, and Gly329. These interactions, which are in line with those observed for **2** (Figure 4B), help us to rationalize the loss of inhibitory activity in compounds lacking the carboxylic group on the *N*-phenyl ring, such as **16** and **17**. Finally, no steric clashes are observed given the lack of the benzoyl moiety (Figure 4D), which is in line with the increased inhibitory activity of **19** compared to **1**. Interestingly, compound **34**, the most potent KAT8i identified in the study, presenting a phenyl ring in place of the methyl group at the C3 position of the 5-pyrazolone core, is nicely accommodated in the same binding pocket as **19**, with the phenyl portion pointing toward a large cavity defined by residues Ile317, Leu353, and Gly357 (Figure 4F). Conversely, the phenyl moiety clashes with Phe1374 in the active site of KAT3B (Figure 4E).

Compounds **19** and **34** were additionally investigated with 100 ns MD simulations within the active site of KAT8 to assess their binding mode and stability (Supplementary Movies 1–2). Indeed, it has been demonstrated that the correct ligand binding poses in docking are usually stable during MD simulations, when a 2.5 Å RMSD cut-off is applied.^47^ The obtained results (Figure S9) indicate that both compounds are stable within the assessed timeframe and preserve the previously mentioned key interactions. Specifically, both **19** and **34** exhibit RMSD values below the 2.5 Å threshold for >95% of the simulation time.

Compounds **26** and **27**, bearing a bulkier indole group in place of the pyrrole, display a drop in potency toward KAT8 due to a steric clash with Phe270 (Figure S10). Conversely, we observed a higher stabilization inside the KAT3B binding pocket, which appears to be mediated by additional stacking interactions of the indole substituent with His1451, and by the hydrogen bond with Asp1399, resulting in a good KAT3B inhibitory activity (Figure S11A). Finally, all the analyzed compounds, independently of their size and scaffold, were not able to inhibit KAT2B. This may be explained by the evidence that the active site of KAT2B is narrower than that of KAT8 and occupied by bulky residues such as Tyr612 and Gln525 (Figure S11B).

### 2.7 Target Engagement of KAT8i **19** and **34** and Effects on Histone Acetylation in Cancer Cells

**Figure 5:**
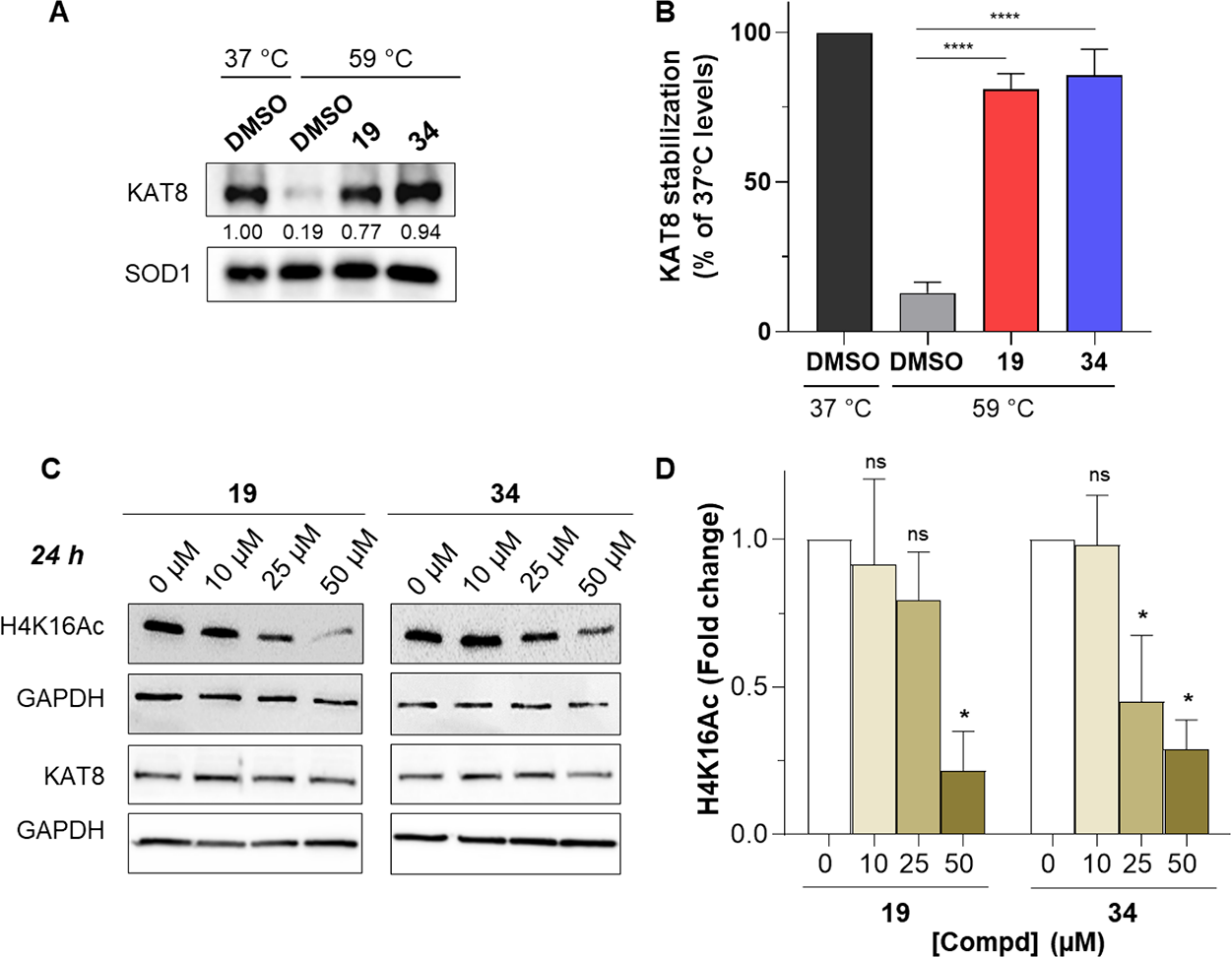
(A) Representative WB showing the target engagement of compounds **19** and **34** at 100 μM tested by CETSA in HT29 cells at 59 °C. (B) Quantification of KAT8 levels by densitometric analysis using the ImageJ software. The relative protein levels are expressed as percentage of KAT8 levels at 37 °C and normalized to SOD1. Experiments were performed in triplicate (*n* = 3). Results represent mean ± SD. The statistical analysis compares KAT8 inhibitor treatment vs control at 59 °C (ns, nonsignificant; ****, *p* < 0.0001; One-way ANOVA). Control (DMSO) consists of 0.5% (*v*/*v*) DMSO-treated cells. (C) WB analysis of H4K16Ac and KAT8 expression levels in the HT29 CRC cell line exposed to increasing doses of compounds **19** and **34** for 24 h. GAPDH has been used as a loading control. (D) Quantification of H4K16Ac levels by densitometric analysis using the ImageJ software. The relative protein levels are expressed as a fold change of treated versus untreated samples, after GAPDH normalization. Experiments were performed in duplicate (*n* = 2). Results represent mean ± SD. The statistical analysis compares KAT8 inhibitor treatment vs control (ns, nonsignificant; *, *p* < 0.05; one-way ANOVA). Control (0 μM) consists of 0.5% (*v*/*v*) DMSO-treated cells.

We then set out to investigate whether KAT8i **19** and **34** would bind their target protein in a cellular setting. To this end, we performed a cellular thermal shift assay (CETSA) in the CRC cell line HT29. To devise the methodology for assessing the target engagement of **19** and **34**, we initially subjected HT29 cells to increasing temperatures and determined the levels of KAT8 levels via Western blot (WB) using thermostable SOD1 as a loading control (Figure S12A). We then selected 59 °C as the temperature to evaluate KAT8 cellular binding as most of the protein had already precipitated at this temperature. Notably, both **19** and **34** exhibited target engagement as they could stabilize KAT8 against thermal-induced aggregation in cells (Figure 5A,B, and Figure S12B).

Since the main catalytic activity of KAT8 consists of acetylating H4K16, we set out to investigate the influence of **19** and **34** on the KAT8 action at the cellular level through WB experiments in HT29 cells. We examined the levels of histone H4K16 acetylation in cell lysates obtained from either control cells or cells treated with increasing concentrations of compounds **19** and **34** for 24 h (Figure 5C,D). Treatment with both compounds induced a decrease in histone H4 acetylation levels compared to those exhibited by control cells. To exclude the possibility that the observed reduction in H4K16 acetylation was due to an effect of both compounds on KAT8 expression, we also assessed the expression level of KAT8 in control and treated cells. Notably, no consistent variation in KAT8 levels was observed in cells following treatment with either compound, thereby suggesting a direct effect of **19** and **34** on KAT8 catalytic activity at the cellular level.

**Figure 6:**
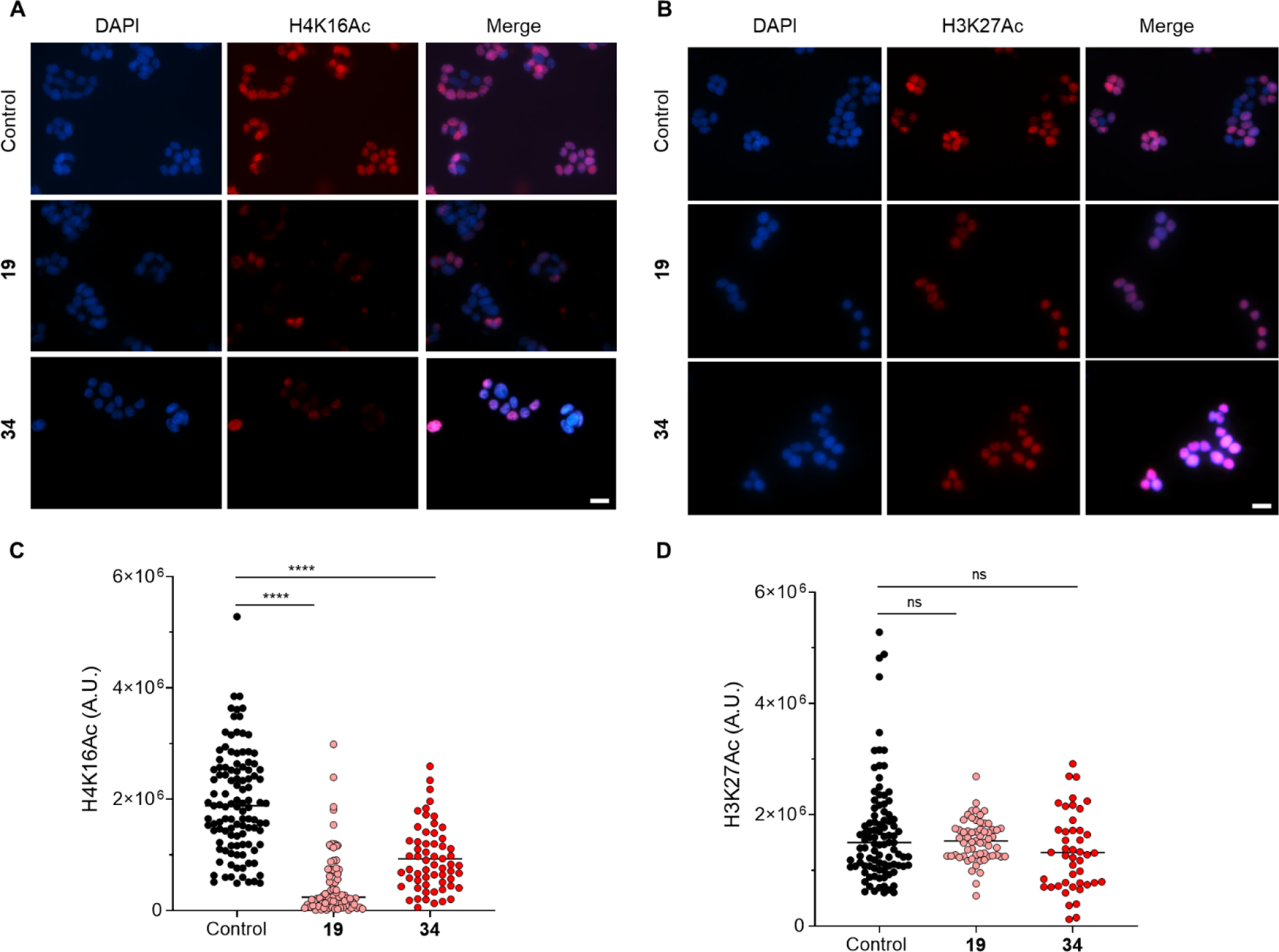
Effect of KAT8i **19** and **34** on histone acetylation in HT29 CRC cell line. (A) IF analysis of histone H4K16 acetylation (H4K16Ac) in HT29 cells exposed to KAT8i **19** and **34** (both at 50 μM). (B) IF analysis of histone H3K27 acetylation (H3K27Ac) in HT29 cells exposed to KAT8i **19** and **34**. The panels depict the patterns observed after 24 h treatment with control or the indicated compounds. Cells were immunostained using primary antibody against histone H4K16Ac that were revealed with Cy3-conjugated secondary antibody. Nuclei were counterstained with DAPI (blue). White scale bars indicate a distance of 20 μm. (C) The scatter plot illustrates the quantitative evaluation of the H4K16Ac signal intensity in at least 50 counted cells per condition. (D) The scatter plot illustrates the quantitative evaluation of the H3K27Ac signal intensity in at least 50 counted cells per condition. The statistical analysis compares KAT8 inhibitor treatment vs control (ns, nonsignificant; ****, *p* < 0.0001; Mann Whitney test). The control consists of 0.5% (*v*/*v*) DMSO-treated cells.

To corroborate these findings, we performed a cell-based immunofluorescence (IF) assay (Figure 6). We examined the levels of H4K16Ac in HT29 cells following 24 h incubation with each KAT8i at a concentration of 50 μM. These experiments revealed that most of the nuclei of control cells were positive for H4K16 acetylation; conversely, a significantly lower staining of the nuclei was observed in cells exposed to **19** and **34** (Figure 6A). This observation was validated by the measurement of fluorescence intensity. We measured an 80% reduction in H4K16Ac signal intensity in cells treated with compound **19**, compared with control, while more than 50% reduction in H4K16Ac was observed in the nuclei of **34**-treated cells (Figure 6C). To assess the cellular target selectivity of the two KAT8i, we also analyzed levels of acetylation at the lysine 27 of histone H3 (H3K27Ac), a residue that is preferentially acetylated by KAT3A/B. Notably, the nuclear staining of H3K27Ac from control and treated cells appeared very similar, and acetylation of histone H3 was observed in most nuclei under all experimental conditions examined (Figure 6B). Accordingly, signal quantification revealed no significant differences in the levels of histone H3K27Ac in control cells and cells treated with either inhibitor (Figure 6D). Overall, the WB and IF data point toward a cellular inhibitory activity of compounds **19** and **34** toward KAT8 catalytic activity with no influence on its expression levels nor on H3K27 acetylation.

### 2.8 Antiproliferative Activity of KAT8 Inhibitors **19** and **34** in a Panel of Cell Lines

**Figure 7:**
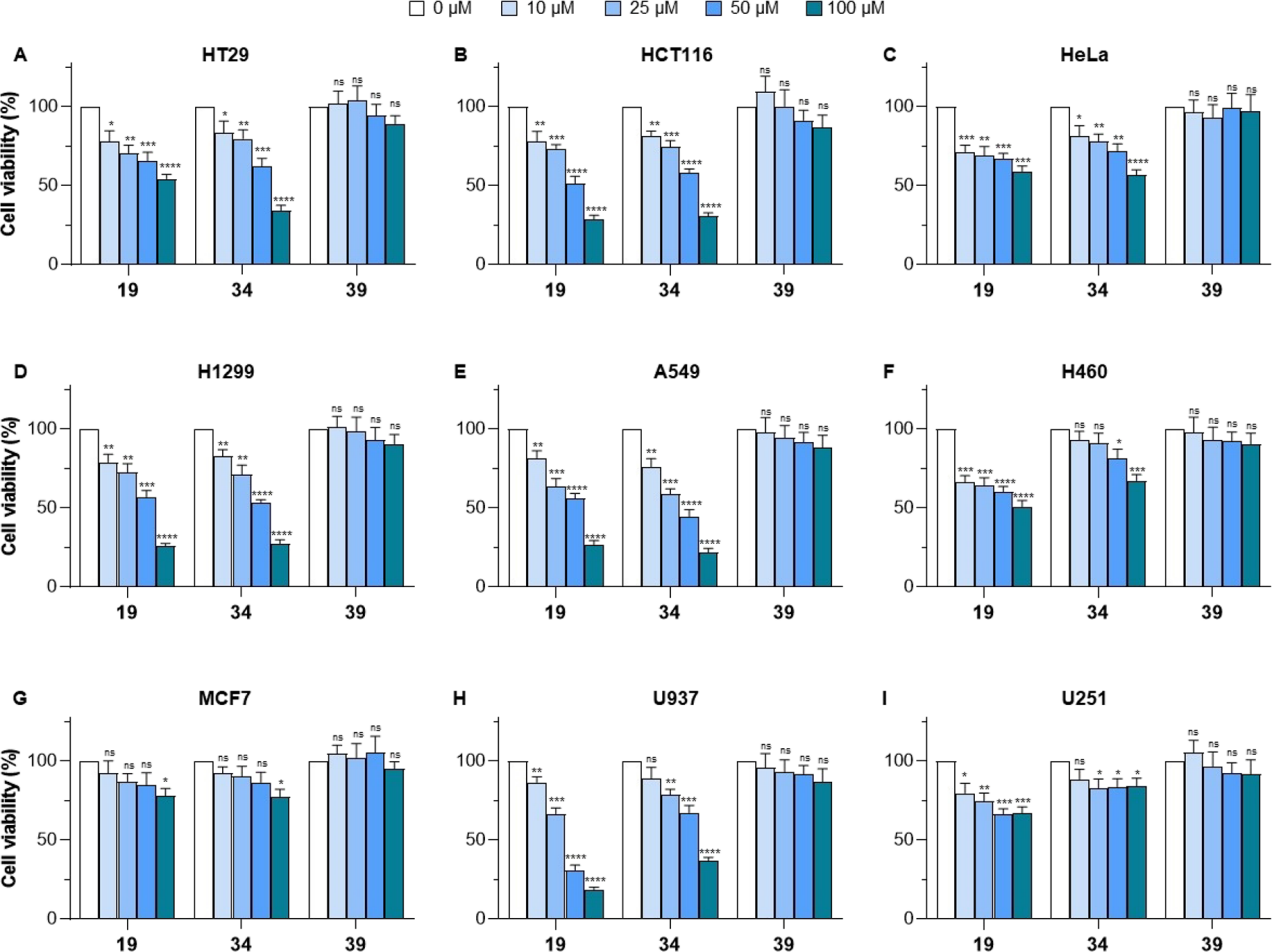
Antiproliferative activity of KAT8i **19** and **34** compared to **39** (negative control) tested at 10, 25, 50, and 100 μM in HT29 (A), HCT116 (B), HeLa (C), H1299 (D), A549 (E), H460 (F), MCF7 (G), U937 (H), and U251 (I) cells for 72 h. The statistical analysis compares KAT8 inhibitor treatment vs control (ns, nonsignificant, *, *p* < 0.05; **, *p* < 0.01; ***, *p* < 0.001****, *p* < 0.0001; multiple *t*-test corrected for multiple comparisons). Control (0 μM) consists of 0.5% DMSO-treated cells.

We then assessed the anticancer potential of compounds **19** and **34** by testing their effects on the proliferation of a panel of different cell lines, including CRC (HT29 and HCT116), uterus cervix cancer (HeLa), NSCLC (H1299, A549, and H460), breast cancer (MCF7), AML (U937), and glioblastoma (U251) cells. To exclude structure-related nonspecific effects on cell proliferation, we also included compound **39** in our analysis, as an internal negative control. We exposed each cell line for 72 h to increasing doses (10, 25, 50, 100 μM) of each inhibitor and measured cell viability through the 3-(4,5-dimethylthiazol-2-yl)-2,5-diphenyltetrazolium bromide (MTT)-based colorimetric assay (Figure 7). Both compounds displayed dose-dependent antiproliferative effects in HCT116, H1299, A549, and U937 cell lines. Compound **19** was significantly active in U937 (∼70% cell proliferation inhibition at 50 μM; >80% cell proliferation inhibition at 100 μM), while compound **34** displayed the highest activity in A549 cells (∼65% cell proliferation inhibition at 50 μM; ∼80% cell proliferation inhibition at 100 μM). Compounds **19** and **34** also exhibited a certain degree of antiproliferative activity in HT29 and HeLa cells, with **34** being more potent in HT29, in which it decreases cell viability by 70% at 100 μM. Conversely, **19** inhibited H460 proliferation, while **34** was essentially inactive. Moreover, neither inhibitor had any effect on the MCF7 and U251 cell lines.

To gain a better understanding of the anticancer activity of compounds **19** and **34**, we tested them at additional concentrations (1.25, 2.5, 5, 10, 25, 50, 100, and 200 μM) in the most sensitive cell lines (HT29, HCT116, H1299, A549, and U937) and determined their respective IC_50_ values (Figure S13). The measured IC_50_ values were in the 30–50 μM range for both inhibitors, with the exception of HT29, and confirmed the observations made with single-point experiments (Table 4). Specifically, **19** displayed lower IC_50_ values compared to **34** in U937 and, to a lesser extent, HCT116 cells, while **34** was more effective in the NSCLC cell lines H1299 and A549.

**Table 4:** IC_50_ Values (μM) of Compounds **19** and **34** after 72 h Treatment in HCT116, H1299, A549, and U937 Cell lines^a^

	IC_50_^b^ (μM), 72 h	
cell line	**19**	**34**
HT29	>100	54.0 ± 10.9
HCT116	39.4 ± 8.2	46.6 ± 11.5
H1299	52.1 ± 10.6	41.6 ± 6.8
A549	41.0 ± 9.8	33.0 ± 5.7
U937	30.1 ± 1.7	51.7 ± 9.5

a Values are means ± standard deviation (SD) of three independent experiments.

b Half maximal inhibitory concentration: dose required to reduce cell viability by 50%.

Overall, the effects of **34** are in line with previous experiments indicating reduced cell proliferation following KAT8 knockdown in H1299 and A549 NSCLC cell lines.^11,12^ Moreover, the cellular activity of **19** is consistent with that of the previously reported KAT8i **VI** that could inhibit the colony formation of HCT116 cells, and of **III** that exerted antiproliferative activity in U937 and other AML cell lines.^16^

**Figure 8:**
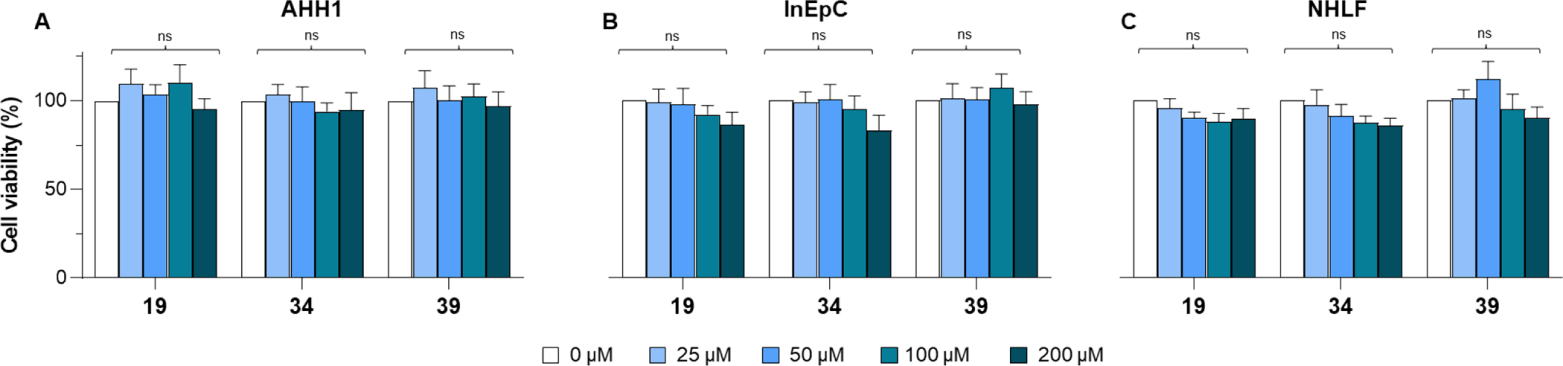
Effect of compounds **19**, **34**, and **39** on the proliferation of (A) human B lymphocyte (AHH1); (B) human intestinal epithelial cells (InEpC); and (C) normal human lung fibroblasts (NHLF) after 72 h incubation at 25, 50, 100, and 200 μM. The statistical analysis compares KAT8 inhibitor treatment vs control (ns, nonsignificant; multiple *t*-test corrected for multiple comparisons). The control (0 μM) consists of 0.5% (*v*/*v*) DMSO-treated cells.

Compounds **19**, **34**, and **39** were also tested at increasing concentrations (25, 50, 100, and 200 μM) in noncancer peripheral blood B lymphocyte AHH-1 cells, human intestinal epithelial cells (InEpC), and normal human lung fibroblasts (NHLF) to assess their differential toxicities. These cell lines act as controls for the cancer cell lines where the antiproliferative activity of compounds **19** and **34** was more evident. After 72 h of treatment, they did not significantly affect the proliferation of any healthy cell line at any of the tested concentrations (Figure 8), thereby suggesting that the observed antiproliferative effects of both KAT8i **19** and **34** can be regarded as cancer-selective.

### 2.9 Influence of KAT8 Inhibitors **19** and **34** on Apoptosis and Oncogene Expression

**Figure 9:**
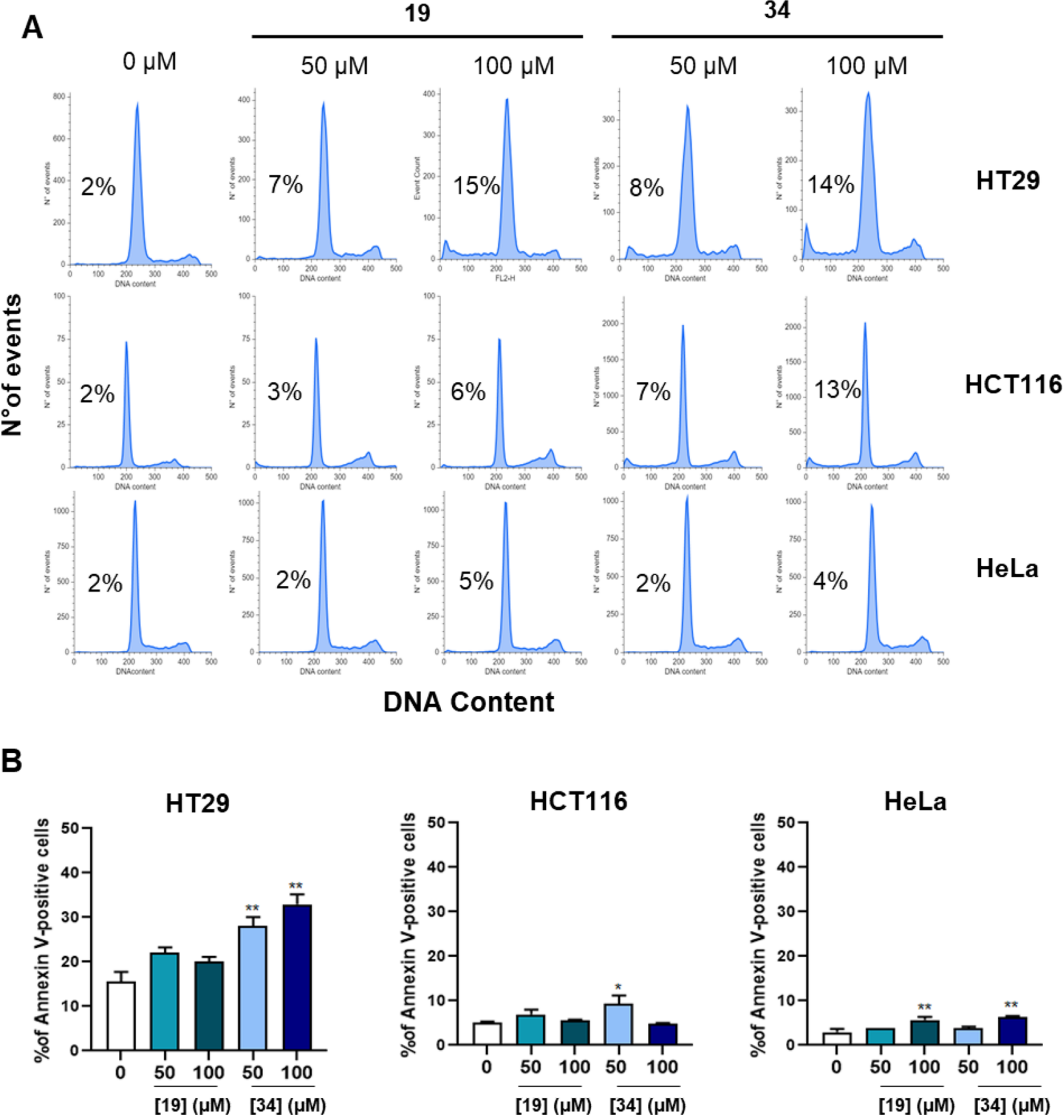
(A) Representative histograms of DNA content evaluated by propidium iodide staining in HT29, HCT-116, and HeLa cells treated with increasing concentrations of compound **19** and **34** for 72 h. The percentage of the sub-G1 peak is also reported. (B) Quantification of Annexin V positive cells in HT29, HCT-116, and HeLa cells treated with compounds **19** and **34** for 72 h. The statistical analysis compares KAT8 inhibitor treatment vs control (*p < 0.05; **p < 0.01; Mann Whitney test).

To gain insights into the influence of **19** and **34** on the cell cycle, we performed additional experiments with **19** and **34** in three different cancer cell lines (HT29, HCT116, and HeLa). As shown in the Figure 9A, cell cycle analysis by propidium iodide (PI) staining showed a slight increase in the percentage of cells with DNA hypodiploid peak, indicative of apoptosis, while no significative change in the different stages of cell cycle was observed in response to both compounds in all tested cell lines. Annexin V/PI analysis confirmed our observations, since only a slight increase in apoptotic cell population was observed in the cell lines when compared to control, with compound **34** being slightly more potent than **19** in all cell lines (Figure 9B). The results described above demonstrate that KAT8i treatment of cancer cells leads both cell proliferation arrest and induction of apoptosis at high doses. Previous studies have shown that KAT8 genetic silencing inhibits cancer cell proliferation mainly via regulation of cell cycle progression.^6,12^ Nevertheless, from these studies, it is not clear if the effects on the cell cycle are due to KAT8 catalytic activity and/or other functions. Moreover, siRNA-mediated downregulation abolishes protein expression almost completely, thereby altering the structural composition of the multiprotein complexes of which KAT8 is a component. Differently, KAT8 catalytic inhibition likely does not alter the stability of such complexes, resulting in less profound effects on cellular homeostasis. Consequently, suppression of the only KAT8 catalytic activity via small molecule inhibitors may not be sufficient to produce the same effects of siRNA-mediated KAT8 downregulation.

**Figure 10:**
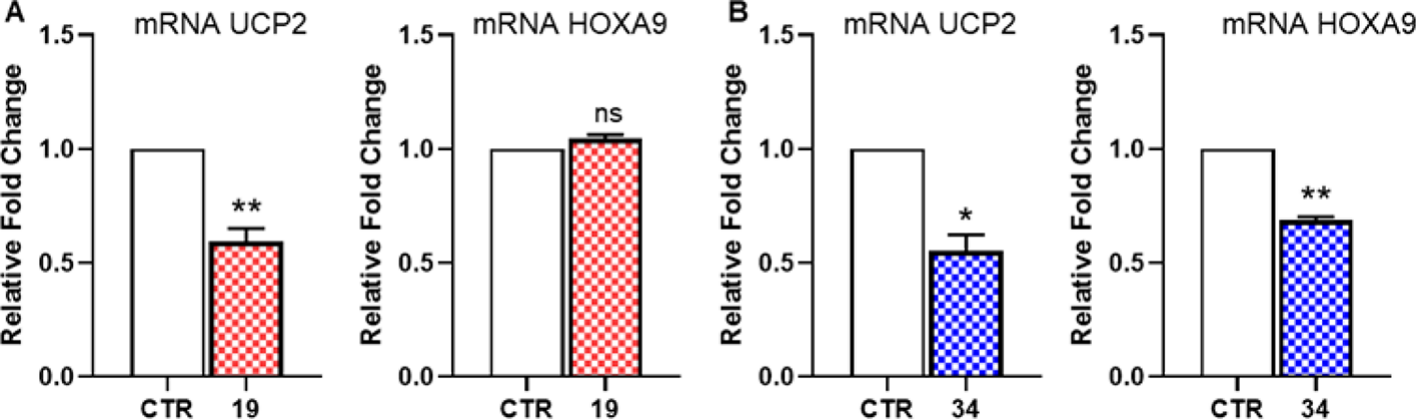
*UCP2* and *HOXA9A* mRNA expression evaluated by quantitative RT-PCR (qRT-PCR) in the HCT116 cell line exposed to 100 μM of compounds **19** (A) and **34** (B) for 48 h. Results are presented as the mean ± SEM of at least two independent experiments. The statistical analysis compares KAT8 inhibitor treatment vs control (*p < 0.05, **p < 0.01; Mann Whitney test).

Moreover, to further explore the antiproliferative mechanism of **19** and **34**, we examined whether they could modulate the expression of oncogenes *UCP2* and *HOXA9*, which have been shown to be downregulated following KAT8 silencing with siRNA.^6^ Interestingly, our analysis revealed that both inhibitors can reduce the mRNA levels of *UCP2*, with **34** being capable of downregulating also *HOXA9* in HCT116 cells (Figure 10). The observed *UCP2* downregulation is consistent with the effect reported in the same cell line for the KAT8i **VI**, which is endowed with an inhibitory potency similar to **19** and **34**, albeit its selectivity profile is unknown.^24^

### 2.10 Influence of KAT8 Inhibitors **19** and **34** on Autophagy

**Figure 11:**
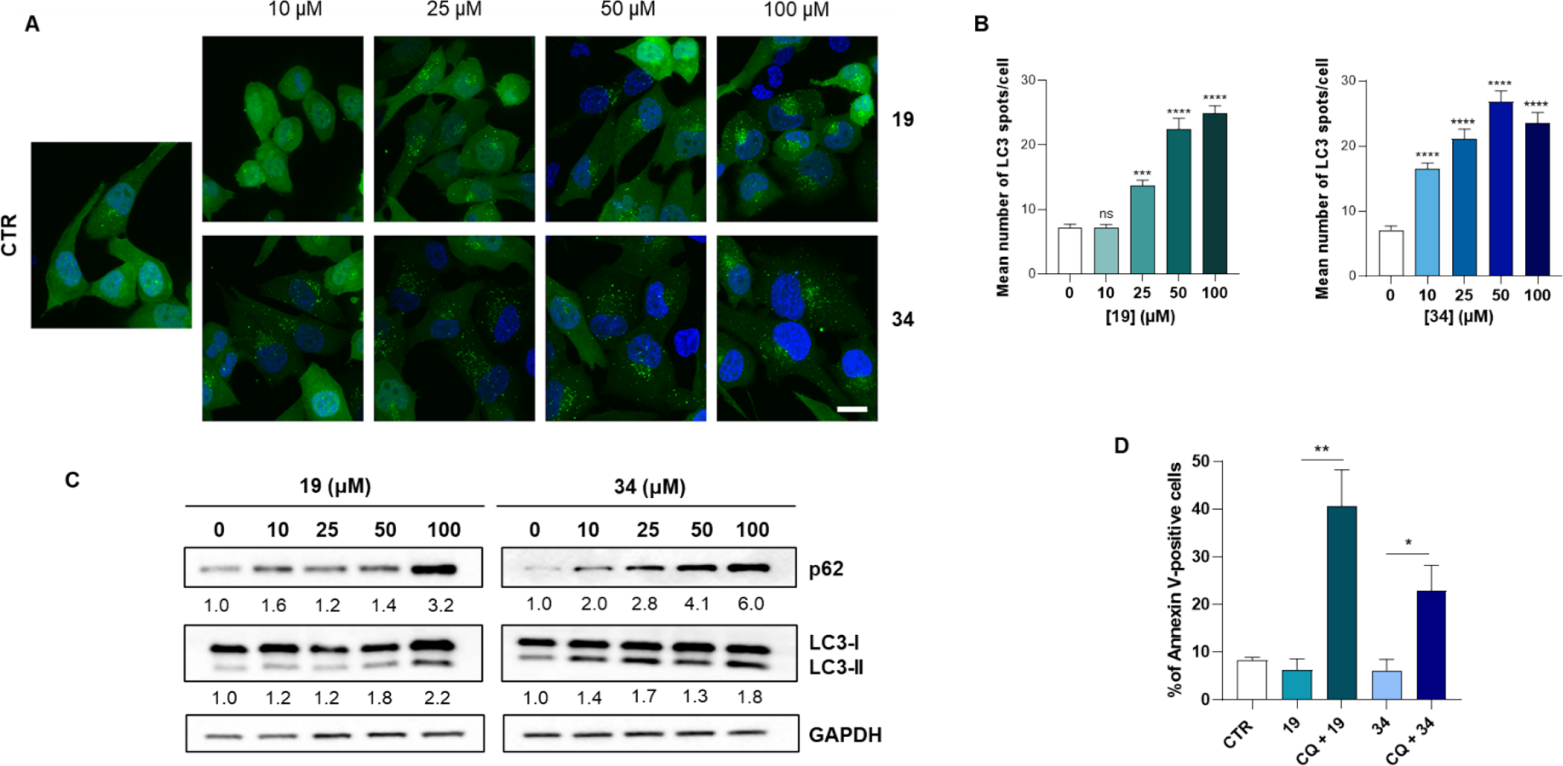
(A) Representative images of EGFP-LC3 H1299 cells exposed compound **19** and **34** for 48 h and stained with DAPI. Scale bar: 20 μm. (B) Quantification of the number of EGFP-LC3 dots/cell in cells treated as in panel A. Data are presented as median with an interquartile range. Symbols represent individual cells. The statistical analysis compares KAT8 inhibitor treatment vs control (****p < 0.0001; Mann Whitney test). (C) WB analysis of autophagic marker expression levels in HCT116 cells exposed to increasing doses of compounds **19** and **34** for 48 h. Cell lysates were collected to assess LC3I/II conversion and SQSTM1/p62 levels by immunoblotting. GAPDH was used as a loading and transferring control. Numbers report the densitometric values of band intensity, obtained by the densitometric analysis of the immunoblots. Values of the LC3-II/I ratio and of p62 were normalized to GAPDH as a loading control and are reported as the fold change (FC) over the control value. (D) Quantification of Annexin V fluorescence upon treatment of HCT116 cells with compounds **19** and **34** alone (100 μM) or in combination with autophagy inhibitor CQ (10 μM) for 72 h. The statistical analysis compares untreated and treated cells (*p < 0.05; Mann Whitney test).

Finally, since deacetylation of H4K16 is associated with autophagy induction and transcriptional regulation of autophagy-related genes,^48^ we characterized the influence of both KAT8i on autophagy. To test autophagy induction, we took advantage of H1299 cells stably expressing EGFP-LC3 (H1299-EGFP-LC3), a cellular tool used to determine the influence of the downregulation or overexpression of specific proteins in the autophagic flux as well as to study autophagy-modulating compounds. The cells were exposed to increasing doses of **19** and **34** for 48 h and observed by fluorescence microscopy (Figure 11A). As shown in Figure 11A,B, treatment with both compounds induced a dose-dependent redistribution of the LC3 protein in the cytoplasm with the formation of autophagosomes appearing as fluorescent spots. EGFP-LC3 dots per cell were significantly increased in response to **19** and **34** (Figure 11A,B). Consistent with these analyses, Western blot experiments showed a modulation of the LC3-II/-I ratio and p62 autophagic markers in HCT116 cells, indicating that both inhibitors trigger the activation of the autophagic process (Figure 11C). A similar response was also observed in the HeLa cell line with compound **34** (Figure S14). Unlike LC3-II, p62 does not usually increase when autophagy is induced. However, p62 upregulation can often be associated to autophagy by additional mechanisms of regulation or implicated in the apoptotic response.^49,50^ In cancer, autophagy has a dual role since it may act as either a stress response mechanism to protect cancer cells from drug insults or eliminate cancer cells by triggering a nonapoptotic cell death program. Therefore, we have also analyzed the impact of KAT8 inhibition on cell death when the autophagic pathway is inhibited by pretreatment with chloroquine (CQ). Interestingly, exposure of HCT116 cells to CQ increased the apoptotic effect of both KAT8i (Figure 11D and Figure S15). This suggests that autophagy activation following KAT8 inhibition might act as a prosurvival response and provides the proof of principle to study the combination of KAT8 and autophagy inhibitors more in depth.

## 3 Conclusions

KAT8 is involved in a plethora of pathways that are crucial for cell cycle regulation and genomic integrity.^6,7,9,10^ Hence, its dysregulation may have severe consequences on cellular homeostasis and may lead to aberrant proliferation,^19,51^ as observed in different cancer contexts, including NSCL, AML, breast cancer, OTSCC, and HCC.^11−18^ Consequently, potent and selective KAT8i would be highly beneficial for the treatment of many cancer types and would also serve as chemical probes to investigate KAT8 biology. Nonetheless, KAT8 drug discovery has been quite challenging, with no selective inhibitors reported to date.

In this study, using the structure of C646 as a starting point, we developed novel *N*-phenyl 3-substituted-4-arylidene-5-pyrazolone derivatives, many of them characterized by selective KAT8 inhibition. The first series of compounds, characterized by the presence of variously substituted 4-benzoyl-1*H*-pyrrole-2-methylidene moieties at the C4 position of the 5-pyrazolone ring, did not yield any potent and/or selective KAT8i (Table 1). The SAR drawn from the second series of derivatives indicate that removal of the benzoyl portion shifts the activity toward KAT8 inhibition, with the 2-pyrrolyl substitution being the preferred one among the different (hetero)aromatic rings assayed. Specifically, compound **19** is a 2-pyrrolylmethylidene containing KAT8i (IC_50_ = 12.1 μM) selective over KAT3B and KAT2B, while the replacement of its pyrrole with other rings (**20**–**29**) leads to a decrease in potency and/or selectivity (Table 2). Notably, compound **31**, the reduced analogue of **19**, exhibited selective KAT8 inhibition, with only a slight decrease in potency (IC_50_ = 17.2 μM). This data indicates that, although important, the exocyclic double bond, and therefore the presence of an α,β-unsaturated system, is not crucial for KAT8 inhibition. Remarkably, replacing the methyl substituent in the 5-pyrazolone core with a phenyl ring (**34**) leads to an increase in inhibitory potency, while substituting the carboxylic group with various amides, sulfonic acid, or sulfonamide (**37**–**43**) is detrimental for compound activity.

The two most potent compounds (**19** and **34**) also displayed selectivity over KAT2A, 5, 6A, 6B, and 7 (Table 3) as well as KDAC1–3, 6, and 8 (Table S3), and biophysical evaluation via SPR confirmed their direct interaction with KAT8, with *K_D_* values consistent with their *in vitro* inhibitory potency (Figure 3). Moreover, HPLC analysis, preincubation assays, and jump-dilution experiments confirmed that both **19** and **34** do not react with thiol-containing nucleophiles and are reversible inhibitors (Figures S4–S6).

Docking studies and MD simulations provided a rationale for the binding mode of **19** and **34** to KAT8, indicating the presence of many hydrogen bonds stabilizing the interaction, along with a cavity accommodating the bulky phenyl group of **34** (Figure 4D,F and Figure S9). Conversely, these compounds engage in only few interactions with KAT3B, with the phenyl ring of compound **34** even causing steric clashes in KAT3B (Figure 4C,E), which explains their isoform selectivity. Evaluation of target engagement by CETSA indicated that both inhibitors can interact with KAT8 in cells (Figure 5A,B and Figure S12B). WB assays indicated that **19** and **34** reduce H4K16Ac levels in HT29 cells (Figure 5), thereby suggesting that both molecules are capable of inhibiting KAT8 in cells. These results were corroborated by IF experiments, which also demonstrated that the inhibitors do not affect H3K27Ac levels (Figure 6). In addition, both compounds exhibited dose-dependent antiproliferative activity in CRC (HCT116), NSCLC (HT1299, A549), and AML (U937) cell lines, with IC_50_ values in the mid-micromolar range (Table 4). Moreover, the structurally related compound **39**, which did not display *in vitro* KAT8 inhibition, lacked antiproliferative activity. This data allows us to likely exclude the possibility that the anticancer activity of the tested KAT8i is due to nonspecific modes of action deriving from the presence of a common *N*-phenyl 3-substituted-4-arylidene-5-pyrazolone chemotype. Our results demonstrate that the reduction in the cell viability observed with KAT8i **19** and **34** in specific cell lines (HT-29, HCT116, and HeLa) is associated with apoptosis induction. Importantly, KAT8 inhibition also induced the activation of the autophagic program. Moreover, the suppression of autophagy upon CQ treatment significantly exacerbated the apoptosis triggered by KAT8i in HCT116 cells, suggesting that autophagy might have a prosurvival function in response to KAT8 inhibition. In addition, both KAT8i **19** and **34** exhibited cancer-selectivity as they did not affect the viability of noncancerous B lymphocyte AHH1 cells, human intestinal epithelial cells (InEpC), and normal human lung fibroblasts (NHLF) (Figure 8).

Overall, in this study, we identified first-in-class selective KAT8i through a molecular pruning strategy applied to a first series of pyrrole-containing analogues of the KAT3B/KDAC inhibitor C646. In this way, we obtained two derivatives (**19** and **34**) endowed with an activity profile sensibly different from that of C646. Indeed, despite being only micromolar KAT8i, **19** and **34** exhibited great selectivity over a panel of KATs and KDACs, also displaying the capability to target KAT8 and to exert cancer-selective antiproliferative effects in cells. Thus, the findings of this study suggest the use of **19** and **34** as chemical tools for better defining the biological roles and the therapeutic potential of KAT8 and support the idea that KAT8 inhibition may have therapeutic value for cancer treatment. Finally, given their simple structure and promising activity, the KAT8i presented here can represent valuable starting points for the development of optimized derivatives selectively targeting KAT8 with increased potency.

## 4 Experimental Section

### 4.1 Chemistry

Melting points were determined on a Buchi 530 melting point apparatus. ^1^H-NMR and ^13^C-NMR spectra were recorded at 400 and 100 MHz, respectively, with a Bruker AC400 spectrometer. Chemical shifts are reported in δ (ppm) units relative to the internal reference tetramethylsilane (Me_4_Si). Microwave-assisted reactions were performed with a Biotage Initiator (Uppsala, Sweden) high-frequency microwave synthesizer working at 2.45 GHz, fitted with a magnetic stirrer and sample processor; reaction vessels were Biotage microwave glass vials sealed with an applicable cap; temperature was controlled through the internal IR sensor of the microwave apparatus. Low-resolution mass spectra of final and intermediate compounds were recorded on an API-TOF Mariner by Perspective Biosystem (Stratford, Texas, USA), samples were injected by a Harvard pump using a flow rate of 5–10 μL/min, infused in the Electrospray system. All compounds were routinely checked by TLC and ^1^H-NMR; all final compounds were also checked by ^13^C-NMR. TLC was performed on aluminum-backed silica gel plates (Merck DC, Alufolien Kieselgel 60 F254) with spots visualized by UV light. All solvents were reagent grade and, when necessary, were purified and dried by standard methods. Concentration of solutions after reactions and extractions involved the use of a rotary evaporator operating at reduced pressure of ca. 20 Torr. Organic solutions were dried over anhydrous sodium sulfate. Elemental analysis has been used to determine the purity of all final compounds that is >95%. Analytical results are within ±0.40% of the theoretical values. The purity of the final compounds tested in cells **19**, **34**, and **39** was analyzed by HPLC. The HPLC system consisted of a Dionex UltiMate 3000 UHPLC (Thermo Fisher) system equipped with an automatic injector and column heater and coupled with a Diode Array Detector DAD-3000 (Thermo Fisher). The analytical controls were performed on a Hypersil GOLD C18 Selectivity 5 μm (4.6 × 250 mm) HPLC Column (Thermo Fisher) in gradient elution. Eluents: (A) H_2_O/CH_3_CN, 95/5 (*v*/*v*) + 0.1% TFA; (B) CH_3_CN/H_2_O, 95/5 (*v*/*v*) + 0.1% TFA. A 20 min linear gradient elution from 30% to 100% solvent B was followed by 5 min at 100% B. The flow rate was 1.0 mL/min, and the column was kept at a constant temperature of 30 °C. Samples were dissolved in solvent B at a concentration of 0.25 mg/mL, and the injection volume was 10 μL. All chemicals were purchased from Sigma-Aldrich s.r.l, Milan (Italy), or from TCI Europe N.V., Zwijndrecht (Belgium), and were of the highest purity.

#### 4.1.1 General Procedure for Synthesis of Final Compounds **1**–**15**, **19**, **23**–**27**, **34**–**36**, and **43**

A mixture containing the properly substituted *N*-phenyl-5-pyrazolone (compound **18** or intermediates **59**–**61** and **63**) (0.916 mmol, 1 equiv), the appropriate aldehyde (intermediates **44**–**57** or relevant commercial aryl aldehyde) (0.962 mmol, 1.05 equiv), and diethylamine (0.916 mmol, 0.095 mL, 1 equiv) in dry ethanol (10–20 mL) was stirred at 50 °C under a nitrogen atmosphere for 4–15 h. The reaction mixture was then filtered under vacuum and washed over filter in sequence with dry ethanol, diethyl ether, and petroleum ether. The solid over a filter was triturated with a solution of HCl 2 N (5–15 mL), filtered under vacuum, and washed over filter with water. The solid over a filter was again triturated with the appropriate ethyl acetate/diethyl ether mixture, filtered under vacuum, washed with ethyl acetate or diethyl ether, and recrystallized from the proper solvent to yield final compounds **1**–**15**, **19**, **23**–**27**, **34**–**36**, and **43**.

##### 4.1.1.1 (*Z*)-4-(4-((4-Benzoyl-1*H*-pyrrol-2-yl)methylene)-3-methyl-5-oxo-4,5-dihydro-1*H*-pyrazol-1-yl)benzoic Acid (MC3983, **1**)

^1^H-NMR (400 MHz; DMSO-*d*_6_) δ 2.34 (s, 3H, C*H*_3_), 7.59 (t, 2H, *J* = 7.2 Hz, C*H* benzoyl protons), 7.66–7.72 (m, 2H, C*H* benzoyl + C*H* methine protons), 7.84 (d, 2H, *J* = 7.2 Hz, C*H* benzoyl protons), 7.96 (s, 1H, C*H* pyrrole proton), 8.04 (d, 2H, *J* = 8.8 Hz, C*H* phenyl protons), 8.11 (d, 2H, *J* = 8.8 Hz, C*H* phenyl protons), 8.15 (s, 1H, C*H* pyrrole proton), 13.69 (br s, 1H, N*H*). ^13^C-NMR (100 MHz, DMSO-*d*_6_) δ 12.8, 117.7 (2C), 119.1, 126.5, 126.7, 126.8, 128.71 (2C), 128.76 (2C), 130.41, 130.48 (2C), 132.4, 134.6, 135.7, 138.5, 141.5, 152.4, 163.8, 166.9, 188.9. MS (ESI) *m*/*z:* 398 [M – H]^−^.

##### 4.1.1.2 (*Z*)-4-(3-Methyl-4-((4-(2-nitrobenzoyl)-1*H*-pyrrol-2-yl)methylene)-5-oxo-4,5-dihydro-1*H*-pyrazol-1-yl)benzoic Acid (MC3637, **2**)

^1^H-NMR (400 MHz; DMSO-*d*_6_) δ 2.32 (s, 3H, C*H*_3_), 7.53 (br s, 1H, C*H* methine proton), 7.74 (d, 1H, *J* = 7.2 Hz, C*H* benzoyl proton), 7.82–7.96 (m, 3H, C*H* benzoyl + C*H* pyrrole protons), 8.02–8.11 (m, 5H, C*H* phenyl + C*H* pyrrole protons), 8.26 (d, 1H, *J* = 8.4 Hz, C*H* benzoyl proton), 12.75 (br s, 1H, COOH), 13.64 (br s, 1H, N*H*). ^13^C-NMR (100 MHz, DMSO-*d*_6_) δ 12.8, 117.7 (2C), 119.8, 124.8, 125.2, 126.6, 126.9, 128.9, 130.5 (2C), 130.8, 131.5, 133.9, 134.1, 134.6, 135.5, 141.5, 146.6, 152.5, 163.7, 166.9, 186.8. MS (ESI) *m*/*z:* 443 [M – H]^−^.

##### 4.1.1.3 (*Z*)-4-(3-Methyl-4-((4-(3-nitrobenzoyl)-1*H*-pyrrol-2-yl)methylene)-5-oxo-4,5-dihydro-1*H*-pyrazol-1-yl)benzoic Acid (MC4039, **3**)

^1^H-NMR (400 MHz; DMSO-*d*_6_) δ 2.34 (s, 3H, C*H*_3_), 7.73 (br s, 1H, C*H* methine proton), 7.86–7.92 (m, 2H, C*H* benzoyl + C*H* pyrrole protons), 8.03 (d, 2H, *J* = 8.0 Hz, C*H* phenyl protons), 8.11 (d, 2H, *J* = 8.0 Hz, C*H* phenyl protons), 8.22 (s, 1H, C*H* pyrrole proton), 8.28 (d, 1H, *J* = 6.8 Hz, C*H* benzoyl proton), 8.51 (d, 2H, C*H* benzoyl protons), 13.72 (br s, 1H, N*H*). ^13^C-NMR (100 MHz, DMSO-*d*_6_) δ 12.8, 117.6 (2C), 119.6, 123.2, 126.0, 126.1, 126.58, 126.63, 130.45 (2C), 130.54, 130.7, 134.77, 134.88, 135.4, 139.6, 141.5, 147.8, 152.4, 163.7, 166.8, 186.7. MS (ESI) *m*/*z:* 443 [M – H]^−^.

##### 4.1.1.4 (*Z*)-4-(3-Methyl-4-((4-(4-nitrobenzoyl)-1*H*-pyrrol-2-yl)methylene)-5-oxo-4,5-dihydro-1*H*-pyrazol-1-yl)benzoic Acid (MC4052, **4**)

^1^H-NMR (400 MHz; DMSO-*d*_6_) δ 2.35 (s, 3H, CH_3_), 7.73 (br s, 1H, C*H* methine proton), 7.94 (s, 1H, C*H* pyrrole proton), 8.03–8.12 (m, 6H, C*H* phenyl + C*H* benzoyl protons), 8.17 (s, 1H, C*H* pyrrole proton), 8.40 (d, 2H, *J* = 8.4 Hz, C*H* benzoyl protons), 12.82 (br s, 1H, COOH), 13.73 (br s, 1H, N*H*). ^13^C-NMR (100 MHz, DMSO-*d*_6_) δ 13.2, 118.2 (2C), 120.2, 124.0, 124.3 (2C), 126.7, 129.8, 130.4 (2C), 130.9 (2C), 131.1, 135.3, 135.8, 136.2, 144.2, 149.8, 152.9, 164.2, 167.4, 188.0. MS (ESI) *m*/*z:* 443 [M – H]^−^.

##### 4.1.1.5 (*Z*)-4-(4-((4-(2-Chlorobenzoyl)-1*H*-pyrrol-2-yl)methylene)-3-methyl-5-oxo-4,5-dihydro-1*H*-pyrazol-1-yl)benzoic Acid (MC3987, **5**)

^1^H-NMR (400 MHz; DMSO-*d*_6_) δ 2.32 (s, 3H, CH_3_), 7.49–7.63 (m, 5H, C*H* benzoyl + C*H* methine protons), 7.91 (s, 1H, C*H* pyrrole proton), 7.98 (s, 1H, C*H* pyrrole proton), 8.03 (d, 2H, *J* = 8.4 Hz, C*H* phenyl protons), 8.10 (d, 2H, *J* = 8.4 Hz, C*H* phenyl protons), 13.62 (br s, 1H, N*H*). ^13^C-NMR (100 MHz, DMSO-*d*_6_) δ 12.8, 117.7 (2C), 119.7, 125.8, 126.6, 127.40, 127.42, 128.8, 129.6, 130.1, 130.5 (2C), 130.8, 131.7, 134.6, 135.6, 138.9, 141.5, 152.5, 163.8, 166.9, 188.1. MS (ESI) *m*/*z:* 432 [M – H]^−^.

##### 4.1.1.6 (*Z*)-4-(4-((4-(3-Chlorobenzoyl)-1*H*-pyrrol-2-yl)methylene)-3-methyl-5-oxo-4,5-dihydro-1*H*-pyrazol-1-yl)benzoic Acid (MC4008, **6**)

^1^H-NMR (400 MHz; DMSO-*d*_6_) δ 2.34 (s, 3H, C*H*_3_), 7.62 (t, 1H, C*H* benzoyl proton), 7.69 (s, 1H, C*H* methine proton), 7.74–7.81 (m, 3H, C*H* benzoyl protons), 7.94 (s, 1H, C*H* pyrrole proton), 8.04 (d, 2H, *J* = 8.6 Hz, C*H* phenyl protons), 8.11 (d, 2H, *J* = 8.6 Hz, C*H* phenyl protons), 8.17 (s, 1H, C*H* pyrrole proton), 12.98 (br s, 1H, COO*H*), 13.72 (br s, 1H, N*H*). ^13^C-NMR (100 MHz, DMSO-*d*_6_) 12.8, 117.7 (2C), 119.4, 126.3, 126.6, 127.4, 128.2, 130.47 (2C), 130.54, 130.7, 132.1, 133.5, 134.7, 135.6, 140.4, 141.5, 152.4, 163.8, 166.9, 170.4, 187.4. MS (ESI) *m*/*z:* 432 [M – H]^−^.

##### 4.1.1.7 (*Z*)-4-(4-((4-(4-Chlorobenzoyl)-1*H*-pyrrol-2-yl)methylene)-3-methyl-5-oxo-4,5-dihydro-1*H*-pyrazol-1-yl)benzoic Acid (MC4049, **7**)

^1^H-NMR (400 MHz; DMSO-*d*_6_) δ 2.35 (s, 3H, C*H*_3_), 7.66 (d, 2H, *J* = 8.0 Hz, C*H* benzoyl protons), 7.71 (s, 1H, C*H* methine proton), 7.87 (d, 2H, *J* = 8.0 Hz, C*H* benzoyl protons), 7.94 (s, 1H, C*H* pyrrole proton), 8.01–8.07 (m, 4H, C*H* phenyl protons), 8.23 (s, 1H, C*H* pyrrole proton), 13.79 (br s, 1H, N*H*). ^13^C-NMR (100 MHz, DMSO-*d*_6_) δ 14.2, 118.3 (2C), 120.7, 123.7, 126.5, 128.9, 130.6 (2C), 130.9, 131.5 (2C), 132.3, 133.9 (2C), 134.3, 135.1, 135.8, 140.2, 154.3, 163.6, 167.4, 187.8. MS (ESI) *m*/*z:* 432 [M – H]^−^.

##### 4.1.1.8 (*Z*)-4-(4-((4-(2-Fluorobenzoyl)-1*H*-pyrrol-2-yl)methylene)-3-methyl-5-oxo-4,5-dihydro-1*H*-pyrazol-1-yl)benzoic Acid (MC3996, **8**)

^1^H-NMR (400 MHz; DMSO-*d*_6_) δ 2.33 (s, 3H, CH_3_), 7.38–7.42 (m, 2H, C*H* benzoyl protons), 7.61–7.69 (m, 3H, C*H* benzoyl + C*H* methine protons), 7.93 (s, 1H, C*H* pyrrole proton), 8.03–8.12 (m, 5H, C*H* phenyl + C*H* pyrrole protons), 13.65 (br s, 1H, N*H*). ^13^C-NMR (100 MHz, DMSO-*d*_6_) δ 13.2, 116.8, 117.0, 118.1 (2C), 120.0, 125.23, 125.25, 126.3, 127.1, 127.97, 128.06, 128.2, 130.45, 130.46, 130.9 (2C), 131.1, 133.67, 133.72, 135.0, 136.1, 142.0, 152.9, 158.7, 160.1, 164.2, 167.3, 186.32, 186.33. MS (ESI) *m*/*z:* 416 [M – H]^−^.

##### 4.1.1.9 (*Z*)-4-(4-((4-(3-Fluorobenzoyl)-1*H*-pyrrol-2-yl)methylene)-3-methyl-5-oxo-4,5-dihydro-1*H*-pyrazol-1-yl)benzoic Acid (MC4023, **9**)

^1^H-NMR (400 MHz; DMSO-*d*_6_) δ 2.34 (s, 3H, C*H*_3_), 7.54–7.69 (m, 5H, C*H* methine + C*H* benzoyl protons), 7.93 (s, 1H, C*H* pyrrole proton), 8.04 (d, 2H, *J* = 7.2 Hz, C*H* phenyl protons), 8.11 (d, 2H, *J* = 7.2 Hz, C*H* phenyl protons), 8.17 (s, 1H, C*H* pyrrole proton), 13.70 (br s, 1H, N*H*). ^13^C-NMR (100 MHz, DMSO-*d*_6_) δ 13.2, 115.7, 115.8, 118.1 (2C), 119.58, 119.70, 119.78, 125.4, 126.79, 126.82, 127.1, 130.9 (2C), 131.0, 131.33, 131.38, 135.1, 136.0, 141.09, 141.12, 141.9, 152.8, 161.7, 163.1, 164.2, 167.3, 187.8. MS (ESI) *m*/*z:* 416 [M – H]^−^.

##### 4.1.1.10 (*Z*)-4-(4-((4-(2-Methoxybenzoyl)-1*H*-pyrrol-2-yl)methylene)-3-methyl-5-oxo-4,5-dihydro-1*H*-pyrazol-1-yl)benzoic Acid (MC3991, **10**)

^1^H-NMR (400 MHz; DMSO-*d*_6_) δ 2.32 (s, 3H, C*H*_3_), 3.76 (s, 3H, OC*H*_3_), 7.08 (t, 1H, *J* = 7.6 Hz, C*H* benzoyl proton), 7.21 (d, 1H, *J* = 8.4 Hz, C*H* benzoyl proton), 7.35 (d, 1H, *J* = 7.6 Hz, C*H* benzoyl proton), 7.51–7.55 (m, 2H, C*H* benzoyl + C*H* methine protons), 7.90 (s, 1H, C*H* pyrrole proton), 7.95 (s, 1H, C*H* pyrrole proton), 8.03 (d, 2H, *J* = 8.4 Hz, C*H* phenyl protons), 8.11 (d, 2H, *J* = 8.4 Hz, C*H* phenyl protons), 13.58 (br s, 1H, N*H*). ^13^C-NMR (100 MHz, DMSO-*d*_6_) δ 12.8, 55.6, 112.1, 117.6 (2C), 118.9, 120.4, 126.0, 126.5, 128.5, 128.6, 129.44, 130.39, 130.47 (2C), 131.8, 134.5, 135.8, 141.6, 152.4, 156.4, 163.8, 166.8, 189.1. MS (ESI) *m*/*z:* 428 [M – H]^−^.

##### 4.1.1.11 (*Z*)-4-(4-((4-(3-Methoxybenzoyl)-1*H*-pyrrol-2-yl)methylene)-3-methyl-5-oxo-4,5-dihydro-1*H*-pyrazol-1-yl)benzoic Acid (MC4028, **11**)

^1^H-NMR (400 MHz; DMSO-*d*_6_) δ 2.34 (s, 3H, C*H*_3_), 3.85 (s, 3H, OC*H*_3_), 7.25–7.50 (m, 4H, C*H* benzoyl protons), 7.70 (s, 1H, C*H* methine proton), 7.95–8.17 (m, 6H, C*H* phenyl + *CH* pyrrole protons), 12.99 (br s, 1H, COO*H*), 13.68 (br s, 1H, N*H*). ^13^C-NMR (100 MHz, DMSO-*d*_6_) δ 13.2, 55.8, 113.9, 118.1 (2C), 118.8, 119.6, 121.6, 126.9, 127.0, 127.3, 130.3, 130.87, 130.92 (2C), 135.1, 136.1, 140.3, 142.0, 152.9, 159.7, 164.3, 167.3, 188.9. MS (ESI) *m*/*z*: 428 [M – H]^−^.

##### 4.1.1.12 (*Z*)-4-(4-((4-(2-Hydroxybenzoyl)-1*H*-pyrrol-2-yl)methylene)-3-methyl-5-oxo-4,5-dihydro-1*H*-pyrazol-1-yl)benzoic Acid (MC4006, **12**)

^1^H-NMR (400 MHz; DMSO-*d*_6_) δ 2.34 (s, 3H, C*H*_3_), 6.96–7.02 (m, 2H, C*H* benzoyl proton), 7.47 (t, 1H, *J* = 7.2 Hz, C*H* benzoyl proton), 7.59 (d, 1H, *J* = 7.2 Hz, C*H* benzoyl proton), 7.65 (s, 1H, C*H* methine proton), 7.92 (s, 1H, C*H* pyrrole proton), 8.03–8.13 (m, 5H, C*H* phenyl + *CH* pyrrole protons), 10.79 (br s, 1H, O*H*), 12.95 (br s, 1H, COO*H*), 13.63 (br s, 1H, N*H*). ^13^C-NMR (100 MHz, DMSO-*d*_6_) δ 12.8, 117.1, 117.7 (2C), 119.0, 119.1, 124.3, 126.4, 126.6, 127.6, 130.29, 130.34, 130.5 (2C), 133.7, 134.7, 135.6, 141.6, 152.4, 157.9, 163.8, 166.9, 190.8. MS (ESI) *m*/*z*: 414 [M – H]^−^.

##### 4.1.1.13 (*Z*)-4-(3-Methyl-4-((4-(2-methylbenzoyl)-1*H*-pyrrol-2-yl)methylene)-5-oxo-4,5-dihydro-1*H*-pyrazol-1-yl)benzoic Acid (MC3989, **13**)

^1^H-NMR (400 MHz; DMSO-*d*_6_) δ 2.31 (s, 3H, C*H*_3_ at C3 of pyrazolone protons), 2.32 (s, 3H, C*H*_3_ at C2 of benzoyl protons), 7.32–7.38 (m, 2H, C*H* benzoyl protons), 7.44–7.48 (m, 2H, C*H* benzoyl protons), 7.53 (br s, 1H, C*H* methine proton), 7.91 (s, 1H, C*H* pyrrole proton), 7.96 (s, 1H, C*H* pyrrole proton), 8.03 (d, 2H, *J* = 8.8 Hz, C*H* phenyl protons), 8.11 (d, 2H, *J* = 8.8 Hz, C*H* phenyl protons), 13.60 (br s, 1H, N*H*). ^13^C-NMR (100 MHz, DMSO-*d*_6_) δ 12.8, 19.8, 117.6 (2C), 119.3, 125.5, 126.2, 126.6, 127.7, 128.3, 130.2, 130.5 (2C), 130.6, 131.0, 134.5, 135.5, 135.7, 139.2, 141.5, 152.4, 163.8, 166.7, 191.3. MS (ESI) *m*/*z*: 412 [M – H]^−^.

##### 4.1.1.14 (*Z*)-4-(3-Methyl-4-((4-(3-methylbenzoyl)-1*H*-pyrrol-2-yl)4ethylene)-5-oxo-4,5-dihydro-1*H*-pyrazol-1-yl)benzoic Acid (MC4020, **14**)

^1^H-NMR (400 MHz; DMSO-*d*_6_) δ 2.34 (s, 3H, C*H*_3_ at C3 of pyrazolone protons), 2.42 (s, 3H, C*H*_3_ at C3 of benzoyl protons), 7.44–7.48 (m, 2H, C*H* benzoyl protons), 7.62–7.68 (m, 3H, C*H* benzoyl + C*H* methine protons), 7.93 (s, 1H, C*H* pyrrole proton), 8.04 (d, 2H, *J* = 8.8 Hz, C*H* phenyl protons), 8.11–8.15 (m, 3H, C*H* phenyl + *CH* pyrrole protons), 13.69 (br s, 1H, N*H*). ^13^C-NMR (100 MHz, DMSO-*d*_6_) δ 13.2, 21.4, 118.1 (2C), 119.5, 126.5, 126.9, 127.0, 127.4, 129.0, 129.6, 130.8, 130.9 (2C), 133.4, 135.1, 136.1, 138.5, 139.0, 142.0, 152.9, 164.3, 167.3, 189.3. MS (ESI) *m*/*z*: 412 [M – H]^−^.

##### 4.1.1.15 (*Z*)-4-(3-Isopropyl-4-((4-(2-nitrobenzoyl)-1*H*-pyrrol-2-yl)methylene)-5-oxo-4,5-dihydro-1*H*-pyrazol-1-yl)benzoic Acid (MC4248, **15**)

^1^H-NMR (400 MHz; DMSO-*d*_6_) δ 1.32 (d, 6H, *J* = 6.8 Hz, 2 × C*H*_3_ isopropyl protons), 3.14–3.21 (m, 1H, C*H* isopropyl protons), 7.56 (br s, 1H, C*H* methine proton), 7.74 (d, 1H, *J* = 7.6 Hz, C*H* benzoyl proton), 7.85 (t, 1H, *J* = 8.0 Hz, C*H* benzoyl proton), 7.93–7.96 (m, 2H, C*H* benzoyl + C*H* pyrrole protons), 8.04–8.06 (m, 3H, C*H* phenyl + C*H* pyrrole protons), 8.11 (d, 2H, *J* = 8.8 Hz, C*H* phenyl protons), 8.26 (s, 1H, *J* = 8.0 Hz, C*H* benzoyl proton), 13.72 (br s, 1H, N*H*). ^13^C-NMR (100 MHz, DMSO-*d*_6_) δ 20.9 (2C), 26.1, 117.8 (2C), 118.2, 124.8, 125.6, 126.7, 126.87, 128.90, 130.5 (2C), 130.7, 131.4, 133.8, 134.6, 135.0, 135.5, 141.6, 146.6, 159.6, 164.0, 166.9, 186.8. MS (ESI) *m*/*z*: 471 [M – H]^−^.

##### 4.1.1.16 (*Z*)-4-(4-((1*H*-Pyrrol-2-yl)methylene)-3-methyl-5-oxo-4,5-dihydro-1*H*-pyrazol-1-yl)benzoic Acid (MC4033, **19**)

^1^H-NMR (400 MHz; DMSO-*d*_6_) δ 2.33 (s, 3H, C*H*_3_), 6.58 (t, 1H, C*H* pyrrole proton), 7.26 (br s, 1H, C*H* methine proton), 7.73 (s, 1H, C*H* pyrrole proton), 7.80 (s, 1H, C*H* pyrrole proton), 8.03 (d, 2H, *J* = 8.8 Hz, C*H* phenyl protons), 8.14 (d, 2H, *J* = 8.8 Hz, C*H* phenyl protons), 12.89 (br s, 1H, COO*H*), 13.60 (br s, 1H, N*H*). ^13^C-NMR (100 MHz, DMSO-*d*_6_) δ 12.8, 114.3, 115.5, 117.6 (2C), 126.3, 127.2, 130.3, 130.5 (2C), 132.2, 135.1, 141.9, 152.4, 164.1, 167.0. MS (ESI) *m*/*z*: 294 [M – H]^−^.

##### 4.1.1.17 (*Z*)-4-(4-((1*H*-Pyrrol-3-yl)methylene)-3-methyl-5-oxo-4,5-dihydro-1*H*-pyrazol-1-yl)benzoic Acid (MC4276, **23**)

^1^H-NMR (400 MHz; DMSO-*d*_6_) δ 2.31 (s, 3H, C*H*_3_), 7.04 (s, 1H, C*H* pyrrole proton), 7.29 (br s, 1H, C*H* methine proton), 7.80 (s, 1H, C*H* pyrrole proton), 7.99 (d, 2H, *J* = 8.4 Hz, C*H* phenyl protons), 8.13 (d, 2H, *J* = 8.4 Hz, C*H* phenyl protons), 8.38 (br s, 1H, C*H* pyrrole proton), 12.01 (br s, 1H, N*H*), 12.80 (br s, 1H, COO*H*). ^13^C-NMR (100 MHz, DMSO-*d*_6_) δ 13.1, 113.7, 116.9 (2C), 118.1, 120.3, 121.5, 125.8, 130.4 (2C), 132.2, 142.3, 142.8, 152.3, 163.0, 167.1. MS (ESI) *m*/*z*: 294 [M – H]^−^.

##### 4.1.1.18 (*Z*)-4-(4-((1*H*-Pyrazol-5-yl)methylene)-3-methyl-5-oxo-4,5-dihydro-1*H*-pyrazol-1-yl)benzoic Acid (MC4283, **24**)

^1^H-NMR (400 MHz; DMSO-*d*_6_) δ 2.37 (s, 3H, C*H*_3_), 7.82–7.98 (m, 2H, C*H* methine + C*H* pyrazole protons), 8.02–8.04 (m, 3H, C*H* phenyl + C*H* pyrazole protons), 8.10 (d, 2H, *J* = 8.8 Hz, C*H* phenyl protons), 12.84 (br s, 1H, COO*H*), 13.94 (br s, 1H, N*H*). ^13^C-NMR (100 MHz, DMSO-*d*_6_) δ 12.9, 116.9, 117.3 (2C), 124.0, 126.2, 130.46, 130.51 (2C), 130.59, 139.6, 141.6, 152.4, 166.9, 172.1. MS (ESI) *m*/*z*: 295 [M – H]^−^.

##### 4.1.1.19 (*Z*)-4-(4-((1*H*-Imidazol-4-yl)methylene)-3-methyl-5-oxo-4,5-dihydro-1*H*-pyrazol-1-yl)benzoic Acid (MC4241, **25**)

^1^H-NMR (400 MHz; DMSO-*d*_6_) δ 2.35 (s, 3H, C*H*_3_), 7.89 (br s, 1H, C*H* methine proton), 8.00–8.12 (m, 5H, C*H* imidazole + C*H* phenyl protons), 8.17 (s, 1H, C*H* imidazole proton), 13.43 (br s, 1H COO*H*). ^13^C-NMR (100 MHz, DMSO-*d*_6_) δ 12.8, 116.7, 117.4 (2C), 119.4, 126.3, 130.45 (2C), 130.52, 140.0, 141.7, 141.8, 152.2, 165.4, 166.9. MS (ESI) *m*/*z*: 295 [M – H]^−^.

##### 4.1.1.20 (*Z*)-4-(4-((1*H*-Indol-2-yl)methylene)-3-methyl-5-oxo-4,5-dihydro-1*H*-pyrazol-1-yl)benzoic Acid (MC4215, **26**)

^1^H-NMR (400 MHz; DMSO-*d*_6_) δ 2.33 (s, 3H, C*H*_3_), 7.15 (t, 1H, *J* = 7.6 Hz, C*H* indole proton), 7.41 (t, 1H, *J* = 7.6 Hz, C*H* indole proton), 7.60 (s, 1H, C*H* methine proton), 7.74–7.79 (m, 2H, C*H* indole protons), 8.03 (s, 1H, C*H* indole proton), 8.06 (d, 2H, *J* = 8.4 Hz, C*H* phenyl protons), 8.15 (d, 2H, *J* = 8.4 Hz, C*H* phenyl protons), 12.83 (s, 1H, N*H*). ^13^C-NMR (100 MHz, DMSO-*d*_6_) δ 12.9, 113.2, 117.7 (2C), 119.1, 121.09, 121.18, 122.6, 126.6, 127.81, 127.84, 130.5 (2C), 133.7, 136.2, 139.6, 141.5, 152.5, 163.6, 166.9. MS (ESI) *m*/*z*: 344 [M – H]^−^.

##### 4.1.1.21 (*Z*)-4-(4-((1*H*-Indol-3-yl)methylene)-3-methyl-5-oxo-4,5-dihydro-1*H*-pyrazol-1-yl)benzoic Acid (MC4282, **27**)^37^

^1^H-NMR (400 MHz; DMSO-*d*_6_) δ 2.45 (s, 3H, C*H*_3_), 7.33–7.35 (m, 2H, C*H* indole protons), 7.60–7.63 (m, 1H, C*H* indole proton), 8.02 (d, 2H, *J* = 8.4 Hz, C*H* phenyl protons), 8.17–8.21 (m, 4H, C*H* phenyl + C*H* indole + C*H* methine protons), 9.82 (s, 1H, C*H* indole proton), 12.76 (br s, 2H, COO*H* + N*H*). ^13^C-NMR (100 MHz, DMSO-*d*_6_) δ 13.1, 112.5, 113.1, 116.9 (2C), 117.7, 118.8, 122.3, 123.7, 125.6, 128.2, 130.4 (2C), 136.5, 138.0, 138.7, 142.4, 152.2, 163.3, 167.0. MS (ESI) *m*/*z*: 344 [M – H]^−^.

##### 4.1.1.22 (*Z*)-4-(4-((1*H*-Pyrrol-2-yl)methylene)-5-oxo-3-phenyl-4,5-dihydro-1*H*-pyrazol-1-yl)benzoic Acid (MC4171, **34**)

^1^H-NMR (400 MHz; DMSO-*d*_6_) δ 6.60 (s, 1H, C*H* pyrrole proton), 7.38 (s, 1H, C*H* methine proton), 7.59–7.60 (m, 3H, C*H* phenyl protons), 7.74–7.75 (m, 3H, C*H* phenyl + C*H* pyrrole protons), 7.80 (s, 1H, C*H* pyrrole proton), 8.07 (d, 2H, *J* = 8.7 Hz, C*H* carboxyphenyl protons), 8.23 (d, 2H, *J* = 8.7 Hz, C*H* carboxyphenyl protons), 12.93 (br s, 1H, COO*H*), 13.81 (br s, 1H, N*H*). ^13^C-NMR (100 MHz, DMSO-*d*_6_) δ 114.0, 115.2, 118.6 (2C), 127.2, 129.2 (2C), 129.3, 129.5 (2C), 130.2, 130.9 (2C), 131.1, 131.3, 133.8, 136.7, 142.3, 153.8, 164.7, 167.4. MS (ESI) *m*/*z*: 356 [M – H]^−^.

##### 4.1.1.23 (*Z*)-4-(4-((1*H*-Pyrrol-2-yl)methylene)-3-benzyl-5-oxo-4,5-dihydro-1*H*-pyrazol-1-yl)benzoic Acid (MC4280, **35**)

^1^H-NMR (400 MHz; DMSO-*d*_6_) δ 4.12 (s, 2H, C*H*_2_), 6.57 (s, 1H, C*H* pyrrole proton), 7.22–7.25 (m, 2H, C*H* methine + C*H* phenyl protons), 7.32–7.35 (m, 2H, C*H* phenyl protons), 7.40–7.42 (m, 2H, C*H* phenyl protons), 7.74 (s, 1H, C*H* pyrrole proton), 7.84 (s, 1H, C*H* pyrrole proton), 8.04 (d, 2H, *J* = 8.4 Hz, C*H* carboxyphenyl protons), 8.14 (d, 2H, *J* = 8.4 Hz, C*H* carboxyphenyl protons), 13.63 (br s, 1H, N*H*). ^13^C-NMR (100 MHz, DMSO-*d*_6_) δ 32.6, 114.3, 114.5, 117.8 (2C), 126.6 (2C), 127.64, 128.6 (4C), 130.2, 130.5 (2C), 132.8, 135.2, 137.6, 141.9, 154.1, 164.1, 166.9. MS (ESI) *m*/*z*: 370 [M – H]^−^.

##### 4.1.1.24 (*Z*)-3-(4-((1*H*-Pyrrol-2-yl)methylene)-3-methyl-5-oxo-4,5-dihydro-1*H*-pyrazol-1-yl)benzoic Acid (MC4284, **36**)

^1^H-NMR (400 MHz; DMSO-*d*_6_) δ 2.34 (s, 3H, C*H*_3_), 6.57 (s, 1H, C*H* pyrrole proton), 7.25 (br s, 1H, C*H* methine proton), 7.57 (t, 1H, *J* = 7.8 Hz, C*H* phenyl proton), 7.72 (s, 1H, C*H* pyrrole proton), 7.76–7.79 (m, 2H, C*H* pyrrole + C*H* phenyl protons), 8.20 (d, *J* = 7.8 Hz, 1H, C*H* phenyl proton), 8.65 (s, 1H, C*H* phenyl proton), 13.66 (br s, 1H, N*H*). ^13^C-NMR (100 MHz, DMSO-*d*_6_) δ 12.8, 114.1, 115.7, 119.0, 122.3, 125.3, 127.0, 129.3, 130.2, 131.5, 132.0, 135.0, 138.7, 151.9, 163.8, 167.1. MS (ESI) *m*/*z*: 294 [M – H]^−^.

##### 4.1.1.25 (*Z*)-4-(4-((1*H*-Pyrrol-2-yl)methylene)-3-methyl-5-oxo-4,5-dihydro-1*H*-pyrazol-1-yl)benzenesulfonamide (MC4285, **43**)

^1^H-NMR (400 MHz; DMSO-*d*_6_) δ 2.34 (s, 3H, C*H*_3_), 6.59 (s, 1H, C*H* pyrrole proton), 7.27 (br s, 1H, C*H* methine proton), 7.35 (s, 2H, SON*H*_2_), 7.74 (s, 1H, C*H* pyrrole proton), 7.81 (s, 1H, C*H* pyrrole proton), 7.91 (d, 2H, *J* = 8.8 Hz, C*H* benzenesulfonamide ring protons), 8.19 (d, 2H, *J* = 8.8 Hz, C*H* benzenesulfonamide ring protons), 13.59 (br s, 1H, N*H*). ^13^C-NMR (100 MHz, DMSO-*d*_6_) δ 12.8, 114.3, 115.4, 117.8 (2C), 126.8 (2C), 127.3, 130.2, 132.3, 135.2, 139.5, 140.9, 152.4, 164.1. MS (ESI) *m*/*z*: 331 [M + H]^+^.

#### 4.1.2 General Procedure for Synthesis of Final Compounds **20**–**22**, **28**–**30**, **33**, and **42**

A mixture containing the properly substituted *N*-phenyl-5-pyrazolone (compound **18** or intermediates **58** and **62**) (0.916 mmol, 1 equiv), the appropriate commercial aldehyde or 2-acetylpyrrole (for compound **30**) (0.962 mmol, 1.05 equiv), and diethylamine (0.916 mmol, 0.095 mL, 1 equiv) in dry ethanol (5–15 mL) was stirred at 50 °C (rt for final compounds **20**–**22**) under a nitrogen atmosphere for 5–28 h. The reaction mixture was concentrated under reduced pressure to volume of about 1 mL and triturated with a solution of HCl 2 N (5–15 mL), filtered under vacuum, and washed over filter with water. The filtration of the suspension afforded the crude compound that was purified by silica gel column chromatography eluting with the appropriate chloroform (or dichloromethane)/methanol/acetic acid mixture (for compounds **20**–**22**, **28**–**30**, **33**) or ethyl acetate/THF/methanol/acetic acid (40:50:10:1, for compound **42**) to afford final compounds **20**–**22**, **28**–**30**, **33**, and **42**.

##### 4.1.2.1 4-(4-(Furan-2-ylmethylene)-3-methyl-5-oxo-4,5-dihydro-1*H*-pyrazol-1-yl)benzoic Acid (MC4155, **20**)

*Z*/*E* (3:1) mixture.^36^^1^H-NMR (400 MHz; DMSO-*d*_6_) δ 2.35 (s, 3H, C*H*_3_**-** major isomer), 2.67 (s, 1H, C*H*_3_**-** minor isomer), 6.92 (br s, 0.33 H, 1 × C*H* furan proton **-** minor isomer), 6.97 (d, 1H, *J* = 3.8 Hz, 1 × C*H* furan proton **-** major isomer), 7.60 (s, 0.3 H, 1 × C*H* methine proton **-** minor isomer), 7.68 (d, 0.3 H, *J* = 3.7 Hz, 1 × C*H* furan proton **-** minor isomer), 7.75 (s, 1H, 1 × C*H* methine proton major **-** isomer), 8.00–8.10 (m, 5.3 H, 4 × C*H* phenyl protons **-** both isomers), 8.29 (br s, 1.3 H, 1 × C*H* furan proton **-**major isomer +1 × C*H* furan proton **-**minor isomer), 8.64 (d, 1H, *J* = 3.8 Hz, 1 × C*H* furan proton **-** major isomer), 12.86 (br s, 1.3 H, 1 × COO*H* – both isomers). ^13^C-NMR (100 MHz, DMSO-*d*_6_) δ 12.9, 17.7, 114.8, 115.2, 116.9, 117.1, 119.8, 121.0, 125.2, 126.1, 127.8, 128.3, 130.56, 130.64, 131.0, 141.6, 141.8, 149.1, 149.6, 150.3, 150.8, 151.3, 152.0, 162.1, 164.9, 167.0. MS (ESI) *m*/*z*: 295 [M – H]^−^.

##### 4.1.2.2 (*Z*)-4-(3-Methyl-5-oxo-4-(thiophen-2-ylmethylene)-4,5-dihydro-1*H*-pyrazol-1-yl)benzoic Acid (MC4174, **21**)

^1^H-NMR (400 MHz; DMSO-*d*_6_) δ 2.37 (s, 3H, C*H*_3_), 7.40 (t, 1H, *J* = 3.6 Hz, C*H* thiophene proton), 8.02 (d, 2H, *J* = 8.8 Hz, C*H* phenyl protons), 8.10 (d, 2H, *J* = 8.8 Hz, C*H* phenyl protons), 8.23–8.32 (m, 3H, C*H* thiophene + C*H* methine protons), 12.78 (br s, 1H, COO*H*). ^13^C-NMR (100 MHz, DMSO-*d*_6_) δ 13.0, 117.0 (2C), 120.7, 126.0, 128.8, 130.6 (2C), 136.4, 139.0, 140.4, 141.8, 143.0, 152.1, 162.3, 166.9. MS (ESI) *m*/*z*: 311 [M – H]^−^.

##### 4.1.2.3 (*Z*)-4-(4-Benzylidene-3-methyl-5-oxo-4,5-dihydro-1*H*-pyrazol-1-yl)benzoic Acid (MC4156, **22**)

^1^H-NMR (400 MHz; DMSO-*d*_6_) δ 2.39 (s, 3H, C*H*_3_), 7.59 (t, 2H, *J* = 7.4 Hz, C*H* phenyl protons), 7.66 (t, 1H, *J* = 7.4 Hz, C*H* phenyl proton), 7.90 (s, 1H, C*H* methine proton), 8.02 (d, 2H, *J* = 8.8 Hz, C*H* carboxyphenyl protons), 8.08 (d, 2H, *J* = 8.8 Hz, C*H* carboxyphenyl protons), 8.59 (d, 2H, *J* = 7.4 Hz, C*H* phenyl protons), 12.78 (br s, 1H, COO*H*). ^13^C-NMR (100 MHz, DMSO-*d*_6_) δ 13.2, 117.3 (2C), 119.2, 126.4, 128.2, 128.8 (2C), 129.6 (2C), 132.9, 133.5, 133.8 (2C), 141.6, 152.9, 161.9, 166.9. MS (ESI) *m*/*z*: 305 [M – H]^−^.

##### 4.1.2.4 (*Z*)-4-(3-Methyl-4-((1-methyl-1*H*-pyrrol-2-yl)methylene)-5-oxo-4,5-dihydro-1*H*-pyrazol-1-yl)benzoic Acid (MC4217, **28**)

^1^H-NMR (400 MHz; DMSO-*d*_6_) δ 2.37 (s, 3H, C*H*_3_), 3.94 (s, 3H, N-C*H*_3_), 6.44–6.45 (m, 1H, C*H* pyrrole proton), 7.52 (s, 1H, C*H* methine proton), 7.58 (s, 1H, C*H* pyrrole proton), 8.00 (d, 2H, *J* = 8.8 Hz, C*H* phenyl protons), 8.13 (d, 2H, *J* = 8.8 Hz, C*H* phenyl protons), 8.64 (d, 1H, *J* = 3.2 Hz, C*H* pyrrole proton), 12.80 (br s, 1H, COO*H*). ^13^C-NMR (100 MHz, DMSO-*d*_6_) δ 13.0, 34.2, 111.7, 115.7, 116.9 (2C), 125.4, 125.6, 129.3, 130.4 (2C), 131.7, 135.7, 142.4, 152.2, 162.6, 167.0. MS (ESI) *m*/*z*: 308 [M – H]^−^.

##### 4.1.2.5 (*Z*)-4-(3-Methyl-5-oxo-4-((1-phenyl-1*H*-pyrrol-2-yl)methylene)-4,5-dihydro-1*H*-pyrazol-1-yl)benzoic Acid (MC4274, **29**)

^1^H-NMR (400 MHz; DMSO-*d*_6_) δ 2.07 (s, 3H, C*H*_3_), 6.69–6.71 (m, 1H, C*H* pyrrole proton), 7.18 (s, 1H, C*H* methine proton), 7.55–7.76 (m, 5H, C*H* phenyl protons), 7.82 (s, 1H, C*H* pyrrole proton), 8.00 (d, 2H, *J* = 8.8 Hz, C*H* carboxyphenyl protons), 8.12 (d, 2H, *J* = 8.8 Hz, C*H* carboxyphenyl protons), 8.80 (d, 1H, *J* = 3.2 Hz, C*H* pyrrole proton), 12.85 (br s, 1H, COO*H*). ^13^C-NMR (100 MHz, DMSO-*d*_6_) δ 12.6, 112.9, 116.9 (2C), 117.3, 125.2, 125.7, 127.0 (2C), 128.9, 129.1, 129.8 (2C), 130.4 (2C), 131.8, 134.5, 137.3, 142.1, 151.7, 162.3, 167.0. MS (ESI) *m*/*z*: 370 [M – H]^−^.

##### 4.1.2.6 (*Z*)-4-(4-(1-(1*H*-Pyrrol-2-yl)ethylidene)-3-methyl-5-oxo-4,5-dihydro-1*H*-pyrazol-1-yl)benzoic Acid (MC4184, **30**)

^1^H-NMR (400 MHz; DMSO-*d*_6_) δ 2.55 (s, 3H, C*H*_3_ at C3 of pyrazolone ring proton), 2.81 (s, 3H, C*H*_3_ ethylidene proton), 6.58 (s, 1H, C*H* pyrrole proton), 7.52 (s, 1H, C*H* pyrrole proton), 7.72 (s, 1H, C*H* pyrrole proton), 8.03 (d, 2H, *J* = 8.8 Hz, C*H* phenyl protons), 8.14 (d, 2H, *J* = 8.8 Hz, C*H* phenyl protons), 12.83 (br s, 1H, COO*H*), 15.28 (br s, 1H, N*H*). ^13^C-NMR (100 MHz, DMSO-*d*_6_) δ 20.5, 20.8, 114.0, 114.7, 118.4 (2C), 124.7, 126.6, 130.4 (2C), 131.3, 132.9, 141.8, 151.6, 152.3, 164.1, 167.0. MS (ESI) *m*/*z*: 308 [M – H]^−^.

##### 4.1.2.7 (*Z*)-4-(4-((1*H*-Pyrrol-2-yl)methylene)-3-isopropyl-5-oxo-4,5-dihydro-1*H*-pyrazol-1-yl)benzoic Acid (MC4170, **33**)

^1^H-NMR (400 MHz; DMSO-*d*_6_) δ 1.32 (d, 6H, *J* = 6.8 Hz, 2 × C*H*_3_ isopropyl protons), 3.19–3.24 (m, 1H, C*H* isopropyl proton), 6.58 (s, 1H, C*H* pyrrole proton), 7.30 (br s, 1H, C*H* methine proton), 7.72 (s, 1H, C*H* pyrrole proton), 7.87 (s, 1H, C*H* pyrrole proton), 8.04 (d, 2H, *J* = 8.5 Hz, C*H* phenyl protons), 8.16 (d, 2H, *J* = 8.5 Hz, C*H* phenyl protons), 12.89 (br s, 1H, COO*H*), 13.65 (br s, 1H, N*H*). ^13^C-NMR (100 MHz, DMSO-*d*_6_) δ 21.2 (2C), 25.9, 113.9, 114.2, 117.7 (2C), 126.3, 127.4, 130.2, 130.5 (2C), 132.2, 134.5, 142.0, 159.7, 164.4, 167.0. MS (ESI) *m*/*z*: 322 [M – H]^−^.

##### 4.1.2.8 (*Z*)-4-(4-((1*H*-Pyrrol-2-yl)methylene)-3-methyl-5-oxo-4,5-dihydro-1*H*-pyrazol-1-yl)benzenesulfonic Acid (MC4289, **42**)

^1^H-NMR (400 MHz; DMSO-*d*_6_) δ 2.32 (s, 3H, C*H*_3_), 6.56 (s, 1H, C*H* pyrrole proton), 7.24 (br s, 1H, C*H* methine proton), 7.66 (d, 2H, *J* = 8.5 Hz, C*H* benzenesulfonic acid ring protons), 7.70 (s, 1H, C*H* pyrrole proton), 7.77 (s, 1H, C*H* pyrrole proton), 7.94 (d, 2H, *J* = 8.5 Hz, C*H* benzenesulfonic acid ring protons), 13.71 (br s, 1H, N*H*). ^13^C-NMR (100 MHz, DMSO-*d*_6_) δ 12.8, 114.0, 115.9, 117.5 (2C), 126.3 (2C), 126.7, 130.2, 131.8, 134.9, 138.4, 144.5, 151.6, 163.6. MS (ESI) *m*/*z*: 332 [M + H]^+^.

#### 4.1.3 General Procedure for Synthesis of Final Compounds **16** and **17**

A mixture containing intermediate **64** (prepared as previously reported,^40^ 0.366 mmol, 63.8 mg, 1 equiv), the appropriate 4-benzoyl-1*H*-pyrrole-2-carbaldehyde (**44** for **16** and **45** for **17**, 0.366 mmol, 1 equiv), and diethylamine (0.366 mmol, 0.038 mL, 1 equiv) in dry ethanol (5 mL) was stirred at 50 °C under a nitrogen atmosphere for 2–3 h. The reaction mixture was then filtered under vacuum, washed over a filter with dry ethanol, diethyl ether, and petroleum ether, and recrystallized from ethanol to afford final compounds **16** and **17**.

##### 4.1.3.1 (*Z*)-4-((4-Benzoyl-1*H*-pyrrol-2-yl)methylene)-5-methyl-2-phenyl-2,4-dihydro-3*H*-pyrazol-3-one (MC4050, **16**)

^1^H-NMR (400 MHz; DMSO-*d*_6_) δ 2.33 (s, 3H, C*H*_3_), 7.24 (t, 1H, *J* = 7.2 Hz, C*H* phenyl proton), 7.47 (t, 2H, *J* = 7.2 Hz, C*H* phenyl protons), 7.58 (d, 2H, *J* = 7.2 Hz, C*H* benzoyl protons), 7.66–7.68 (m, 2H, C*H* benzoyl + C*H* methine protons), 7.84 (d, 2H, *J* = 7.2 Hz, C*H* benzoyl protons), 7.91 (s, 1H, C*H* pyrrole proton), 7.95 (d, 2H, *J* = 7.2 Hz, C*H* phenyl protons), 8.14 (s, 1H, C*H* pyrrole proton), 13.83 (br s, 1H, N*H*). ^13^C-NMR (100 MHz, DMSO-*d*_6_) δ 12.7, 118.8 (2C), 119.6, 125.1, 126.1, 126.8, 128.69 (2C), 128.76 (2C), 129.0 (2C), 130.5, 132.3, 134.3, 135.3, 138.1, 138.5, 151.6, 163.3, 188.9. MS (ESI) *m*/*z*: 356 [M + H]^+^.

##### 4.1.3.2 (*Z*)-5-Methyl-4-((4-(2-nitrobenzoyl)-1*H*-pyrrol-2-yl)methylene)-2-phenyl-2,4-dihydro-3*H*-pyrazol-3-one (MC4010, **17**)

^1^H-NMR (400 MHz; DMSO-*d*_6_) δ 2.30 (s, 3H, C*H*_3_), 7.24 (t, 1H, *J* = 7.4 Hz, C*H* phenyl proton), 7.44–7.48 (m, 3H, C*H* phenyl + C*H* methine protons), 7.74 (d, 1H, *J* = 7.2 Hz, C*H* benzoyl proton), 7.82–7.86 (m, 2H, C*H* benzoyl + C*H* pyrrole protons), 7.92–7.94 (m, 3H, C*H* phenyl + C*H* benzoyl protons), 8.01 (s, 1H, C*H* pyrrole proton), 8.24–8.26 (m, 1H, C*H* benzoyl proton), 13.78 (s, 1H, N*H*). ^13^C-NMR (100 MHz, DMSO-*d*_6_) δ 12.7, 118.8 (2C), 120.2, 124.8, 125.1, 126.8, 126.9, 128.93, 128.96 (2C), 130.9, 131.4, 133.7, 134.6, 135.1, 135.5, 138.0, 146.6, 151.6, 163.2, 186.8. MS (ESI) *m*/*z*: 401 [M + H]^+^.

#### 4.1.4 Procedure for Synthesis of 4-(4-((1*H*-Pyrrol-2-yl)methyl)-5-hydroxy-3-methyl-1*H*-pyrazol-1-yl)benzoic Acid (MC4264, **31**)

Sodium borohydride (0.186 mmol, 7.04 mg, 1.1 equiv) was added to a solution of **19** (0.169 mmol, 50.0 mg, 1 equiv) in a mixture (6 mL) dry THF/dry methanol (2:1, v/v) at 0 °C, and the resulting reaction mixture was stirred at rt for 4.5 h. After completion, the reaction mixture was evaporated under reduced pressure to give a crude product that was triturated with water (4 mL) with pH corrected to 5–6 with HCl 2 N. The suspension was then filtered under vacuum and washed over filter with water. The crude was then again triturated with diethyl ether (1.5 mL), filtered under vacuum, and washed over filter with petroleum ether to yield the final compound **31** as gray powder. ^1^H-NMR (400 MHz; DMSO-*d*_6_) for prevalent enol form^42,43^ δ 2.05 (s, 3H, C*H*_3_), 3.54 (s, 2H, C*H*_2_), 5.68 (s, 1H, C*H* pyrrole proton), 5.86 (s, 1H, C*H* pyrrole proton), 6.56 (s, 1H, C*H* pyrrole proton), 7.92 (d, 2H, C*H* phenyl proton), 8.00 (d, 2H, C*H* phenyl proton), 10.46 (s, 1H, N*H*), 11.03 (br s, 1H, O*H*), 12.79 (br s, 1H, COO*H*). ^13^C-NMR (100 MHz, DMSO-*d*_6_) δ 12.8, 20.1, 104.8, 107.2, 110.0, 116.3 (2C), 117.5, 126.0, 130.0, 130.5 (2C), 141.6, 149.3, 157.9, 166.9*.* MS (ESI) *m*/*z*: 296 [M – H]^−^.

#### 4.1.5 General Procedure for Synthesis of Final Compounds **37**–**41** (MC4281, MC4273, MC4270, MC4278, MC4277)

A mixture containing **19** (0.338 mmol, 100 ng, 1 equiv), triethylamine (1.35 mmol, 0.189 mL, 4 equiv), and PyBOP (0.406 mmol, 1.2 equiv) in dry DMF (3.5 mL) was stirred at rt under a nitrogen atmosphere for 1 h. Then, ammonia (for compound **37**) or an appropriate commercially available primary/secondary amine (for compounds **38**–**41**) (0.389 mmol, 1.15 equiv) was added and the reaction mixture was stirred at rt under a nitrogen atmosphere for 3.5–4.5 h. After completion, the reaction mixture was quenched by adding water (45 mL) and the resulting precipitate was filtered and washed over the filter with water. The crude product was purified by silica gel column chromatography eluting with the appropriate chloroform/methanol (for compounds **37** and **40**), chloroform/methanol/ammonia (for compound **41**), or ethyl acetate/dichloromethane (for compound **38** and **39**) mixture to afford the desired final compounds **37**–**41**.

##### 4.1.5.1 (*Z*)-4-(4-((1*H*-Pyrrol-2-yl)methylene)-3-methyl-5-oxo-4,5-dihydro-1*H*-pyrazol-1-yl)benzamide (MC4281, **37**)

^1^H-NMR (400 MHz; DMSO-*d*_6_) δ 2.33 (s, 3H, C*H*_3_), 6.56–6.58 (m, 1H, C*H* pyrrole proton), 7.26 (br s, 1H, C*H* methine proton), 7.33 (s, 1H, CONH*H*), 7.72 (s, 1H, C*H* pyrrole proton), 7.79 (s, 1H, C*H* pyrrole proton), 7.96–7.99 (m, 3H, C*H* benzamide ring + CON*H*H protons), 8.08 (d, 2H, *J* = 8.8 Hz, C*H* benzamide ring protons), 13.63 (br s, 1H, N*H* pyrrole proton). ^13^C-NMR (100 MHz, DMSO-*d*_6_) δ 12.8, 114.2, 115.7, 117.5 (2C), 127.0, 128.5 (2C), 129.9, 130.2, 132.0, 135.0, 140.7, 152.1, 163.9, 167.3. MS (ESI) *m*/*z*: 295 [M + H]^+^.

##### 4.1.5.2 (*Z*)-4-(4-((1*H*-Pyrrol-2-yl)methylene)-3-methyl-5-oxo-4,5-dihydro-1*H*-pyrazol-1-yl)-*N*,*N*-dimethylbenzamide (MC4273, **38**)

^1^H-NMR (400 MHz; DMSO-*d*_6_) δ 2.33 (s, 3H, C*H*_3_), 2.98 (s, 6H, N(C*H*_3_)_2_), 6.56–6.58 (m, 1H, C*H* pyrrole proton), 7.25 (br s, 1H, C*H* methine proton), 7.52 (d, 2H, *J* = 8.8 Hz, C*H* benzamide ring protons), 7.71 (s, 1H, C*H* pyrrole proton), 7.79 (s, 1H, C*H* pyrrole proton), 8.05 (d, 2H, *J* = 8.8 Hz, C*H* benzamide ring protons), 13.67 (br s, 1H, N*H*). ^13^C-NMR (100 MHz, DMSO-*d*_6_) δ 12.8, 35.0 (2C), 114.2, 115.7, 117.8 (2C), 126.9, 128.1 (2C), 130.2, 131.9, 132.2, 135.1, 139.2, 151.9, 163.8, 169.8. MS (ESI) *m*/*z*: 323 [M + H]^+^.

##### 4.1.5.3 (*Z*)-4-(4-((1*H*-Pyrrol-2-yl)methylene)-3-methyl-5-oxo-4,5-dihydro-1*H*-pyrazol-1-yl)-*N*-benzylbenzamide (MC4270, **39**)

^1^H-NMR (400 MHz; DMSO-*d*_6_) δ 2.33 (s, 3H, C*H*_3_), 4.50 (d, 2H, C*H*_2_), 6.56–6.58 (m, 1H, C*H* pyrrole proton), 7.24–7.27 (m, 2H, C*H* methine + C*H* phenyl protons), 7.34 (d, 4H, *J* = 8.4 Hz, C*H* phenyl protons), 7.72 (s, 1H, C*H* pyrrole proton), 7.80 (s, 1H, C*H* pyrrole proton), 8.01 (d, 2H, *J* = 8.8 Hz, C*H* benzamide ring protons), 8.11 (d, 2H, *J* = 8.8 Hz, C*H* benzamide ring protons), 9.06 (t, 1H, *J* = 5.6 Hz, CON*H*Bn), 13.63 (br s, 1H, N*H*). ^13^C-NMR (100 MHz, DMSO-*d*_6_) δ 12.8, 42.7, 114.2, 115.7, 117.6, 126.8 (2C), 127.1, 127.3 (2C), 128.28 (2C), 128.36 (2C), 130.0, 130.3, 132.1, 135.1, 139.8, 140.7, 152.2, 164.0, 165.7. MS (ESI) *m*/*z*: 385 [M + H]^+^.

##### 4.1.5.4 (*Z*)-4-(4-((1*H*-Pyrrol-2-yl)methylene)-3-methyl-5-oxo-4,5-dihydro-1*H*-pyrazol-1-yl)-*N*-(2-hydroxyethyl)benzamide (MC4278, **40**)

^1^H-NMR (400 MHz; DMSO-*d*_6_) δ 2.33 (s, 3H, C*H*_3_), 3.35–3.37 (m, 2H, NHC*H*_2_CH_2_OH), 3.51–3.53 (m, 2H, NHCH_2_C*H*_2_OH), 4.75 (t, 1H, *J* = 5.2 Hz, O*H*), 6.56–6.58 (m, 1H, C*H* pyrrole proton), 7.26 (br s, 1H, C*H* methine proton), 7.72 (s, 1H, C*H* pyrrole proton), 7.80 (s, 1H, C*H* pyrrole proton), 7.96 (d, 2H, *J* = 8.8 Hz, C*H* benzamide ring protons), 8.09 (d, 2H, *J* = 8.8 Hz, C*H* benzamide ring protons), 8.45 (t, 1H, *J* = 5.2 Hz, CON*H*), 13.63 (br s, 1H, N*H*). ^13^C-NMR (100 MHz, DMSO-*d*_6_) δ 12.8, 42.2, 59.8, 114.2, 115.7, 117.5 (2C), 127.0, 128.2 (2C), 130.2, 130.2, 132.0, 135.1, 140.6, 152.1, 163.9, 165.7. MS (ESI) *m*/*z*: 339 [M + H]^+^.

##### 4.1.5.5 (*Z*)-4-(4-((1*H*-Pyrrol-2-yl)methylene)-3-methyl-5-oxo-4,5-dihydro-1*H*-pyrazol-1-yl)-*N*-(3-(pyrrolidin-1-yl)propyl)benzamide (MC4277, **41**)

^1^H-NMR (400 MHz; DMSO-*d*_6_) δ 1.69–1.72 (m, 6H, CONH-CH_2_-C*H*_2_-CH_2_-N + 2 × C*H*_2_ pyrrolidine protons), 2.33 (s, 3H, C*H*_3_) 2.43–2.47 (m, 6H, CONH-CH_2_-CH_2_-C*H*_2_-N + 2 × C*H*_2_ pyrrolidine protons), 3.31–3.33 (m, 2H, CONH-C*H*_2_-CH_2_-CH_2_-N protons), 6.57–6.58 (m, 1H, C*H* pyrrole proton), 7.26 (br s, 1H, C*H* methine proton), 7.72 (s, 1H, C*H* pyrrole proton), 7.79 (s, 1H, C*H* pyrrole proton), 7.93 (d, 2H, *J* = 8.8 Hz, C*H* benzamide ring protons), 8.09 (d, 2H, *J* = 8.8 Hz, C*H* benzamide ring protons), 8.57 (t, 1H, *J* = 5.2 Hz, CON*H*), 13.63 (br s, 1H, N*H*). ^13^C-NMR (100 MHz, DMSO-*d*_6_) δ 12.8, 23.1 (2C), 28.3, 38.1, 53.65 (2C), 53.68, 114.2, 115.7, 117.5 (2C), 127.1, 128.1 (2C), 130.23, 130.31, 132.1, 135.1, 140.5, 152.1, 163.9, 165.5. MS (ESI) *m*/*z*: 406 [M + H]^+^.

#### 4.1.6 General Procedure for Synthesis of Carbaldehydes **45**, **46**, **49**, **50**, and **53**–**57**

A solution of oxalyl chloride (8.166 mmol, 1.036 g, 0.691 mL, 1.1 equiv) in dry DCE (5 mL) was added dropwise to a solution of dry DMF (8.166 mmol, 596.9 mg, 0.629 mL, 1.1 equiv) in dry DCE (5 mL) at 0 °C. The reaction mixture was stirred for 10 min at 0 °C, then 10 min at rt, and cooled again at 0 °C. To this mixture was added dropwise at 0 °C a solution of commercially available 1*H*-pyrrole (7.425 mmol, 498.1 mg, 0.515 mL, 1 equiv) in dry DCE (5 mL). The resulting mixture was stirred at rt for 1 h. Then, dry aluminum trichloride (18.56 mmol, 2.475 g, 2.5 equiv) was added to the reaction mixture followed by dropwise addition of a solution of the proper commercial benzoyl chloride (8.538 mmol, 1.1 equiv) in dry DCE (5 mL) at 0 °C. Once the addition of the relevant benzoyl chloride was complete, the reaction mixture was stirred at rt for 5–6 h. Upon the conclusion of the reaction, the mixture was quenched by pouring it onto a mixture of ice (25 g) and 2 N potassium hydroxide solution (5 mL), followed by addition of a solution of sodium hydroxide (50% *w*/*w*, 6 mL). The mixture was stirred for 10 min at 0 °C, supplemented with ethyl acetate (140 mL), and then poured into a separatory funnel. After separation of the first organic phase, the aqueous one was extracted with ethyl acetate (6 × 30 mL). The combined organic phases were dried over sodium sulfate and concentrated under vacuum to furnish a crude that was purified by silica gel column chromatography by eluting with the proper mixture of ethyl acetate/hexane to provide the desired carbaldehydes.

##### 4.1.6.1 4-(2-Nitrobenzoyl)-1*H*-pyrrole-2-carbaldehyde (**45**)

^1^H-NMR (400 MHz; DMSO-*d*_6_) δ 7.34 (s, 1H, C*H* pyrrole proton), 7.68 (d, 2H, C*H* benzoyl + C*H* pyrrole protons), 7.80 (t, 1H, *J* = 7.8 Hz, C*H* benzoyl proton), 7.89 (t, 1H, *J* = 7.8 Hz, C*H* benzoyl proton), 8.21 (d, 1H, *J* = 8.0 Hz, C*H* benzoyl proton), 9.59 (s, 1H, C*H*O), 12.94 (br s, 1H, N*H*).

##### 4.1.6.2 4-(3-Nitrobenzoyl)-1*H*-pyrrole-2-carbaldehyde (**46**)

^1^H-NMR (400 MHz; DMSO-*d*_6_) δ 7.54 (s, 1H, C*H* pyrrole proton), 7.85 (t, 1H, *J* = 7.8 Hz, C*H* benzoyl proton), 7.91 (s, 1H, C*H* pyrrole proton), 8.24 (d, 1H, *J* = 7.6 Hz, C*H* benzoyl proton), 8.48 (d, 2H, *J* = 8.0 Hz, C*H* benzoyl protons), 9.65 (s, 1H, C*H*O), 12.97 (br s, 1H, N*H*).

##### 4.1.6.3 4-(3-Chlorobenzoyl)-1*H*-pyrrole-2-carbaldehyde (**49**)

^1^H-NMR (400 MHz; CDCl_3_) δ 7.47 (t, 2H, C*H* pyrrole + C*H* benzoyl protons), 7.58 (d, 1H, *J* = 8.0 Hz, C*H* benzoyl proton), 7.72–7.75 (m, 2H, C*H* benzoyl + C*H* pyrrole protons), 7.84 (s, 1H, C*H* benzoyl protons), 9.66 (s, 1H, C*H*O), 10.22 (s, 1H, N*H*).

##### 4.1.6.4 4-(4-Chlorobenzoyl)-1*H*-pyrrole-2-carbaldehyde (**50**)

^1^H-NMR (400 MHz; CDCl_3_) δ 7.46 (s, 1H, C*H* pyrrole proton), 7.50 (d, 2H, *J* = 8.4 Hz, C*H* benzoyl protons), 7.73 (m, 1H, C*H* pyrrole proton), 7.83 (d, *J* = 8.4 Hz, 2H, C*H* benzoyl protons), 9.65 (s, 1H, C*H*O), 10.38 (s, 1H, N*H*).

##### 4.1.6.5 4-(2-Methoxybenzoyl)-1*H*-pyrrole-2-carbaldehyde (**53**)

^1^H-NMR (400 MHz; CDCl_3_) δ 3.82 (s, 1H, OC*H*_3_), 7.01–7.07 (m, 2H, C*H* benzoyl protons), 7.38–7.43 (m, 2H, C*H* benzoyl + C*H* pyrrole protons), 7.48 (t, 1H, *J* = 7.9 Hz, C*H* benzoyl proton), 7.68 (s, 1H, C*H* pyrrole proton), 9.59 (s, 1H, C*H*O), 10.15 (s, 1H, N*H*).

##### 4.1.6.6 4-(3-Methoxybenzoyl)-1*H*-pyrrole-2-carbaldehyde (**54**)

^1^H-NMR (400 MHz; CDCl_3_) δ 3.89 (s, 1H, OC*H*_3_), 7.13–7.17 (m, 1H, C*H* benzoyl proton), 7.39–7.44 (m, 3H, C*H* benzoyl + C*H* pyrrole protons), 7.49 (s, 1H, C*H* benzoyl proton), 7.73 (s, 1H, C*H* pyrrole proton), 9.65 (s, 1H, C*H*O), 10.19 (s, 1H, N*H*).

##### 4.1.6.7 4-(2-Hydroxybenzoyl)-1*H*-pyrrole-2-carbaldehyde (**55**)

^1^H-NMR (400 MHz; DMSO-*d*_6_) δ 6.88–6.95 (m, 2H, C*H* benzoyl protons), 7.38–7.43 (m, 2H, C*H* benzoyl + C*H* pyrrole protons), 7.58 (d, 1H, *J* = 7.8 Hz, C*H* benzoyl proton), 7.68 (s, 1H, C*H* pyrrole proton), 9.56 (s, 1H, C*H*O), 10.91 (s, 1H, N*H*), 12.73 (s, 1H, O*H*).

##### 4.1.6.8 4-(2-Methylbenzoyl)-1*H*-pyrrole-2-carbaldehyde (**56**)

^1^H-NMR (400 MHz; CDCl_3_) MHz; DMSO-*d*_6_) δ 2.41 (s, 1H, C*H*_3_), 7.25–7.32 (m, 2H, C*H* benzoyl + C*H* pyrrole protons), 7.38–7.44 (m, 3H, C*H* benzoyl protons), 7.56 (s, 1H, C*H* pyrrole proton), 9.61 (s, 1H, C*H*O), 10.37 (s, 1H, N*H*).

##### 4.1.6.9 4-(3-Methylbenzoyl)-1*H*-pyrrole-2-carbaldehyde (**57**)

^1^H-NMR (400 MHz; CDCl_3_) δ 2.46 (s, 1H, OC*H*_3_), 7.38–7.43 (m, 2H, C*H* benzoyl protons), 7.49 (s, 1H, C*H* pyrrole protons), 7.65–7.68 (m, 2H, C*H* benzoyl protons), 7.73 (s, 1H, C*H* pyrrole proton), 9.65 (s, 1H, C*H*O), 10.27 (s, 1H, N*H*).

#### 4.1.7 General Procedure for Synthesis of Final Compound **32** and Intermediates **58** and **60**

The appropriate β-ketoester (2.44 mmol, 1.15 equiv) was added dropwise to a suspension of the properly substituted commercial phenylhydrazine (2.12 mmol, 1 equiv) in glacial acetic acid (2 mL) at rt. The resulting mixture was stirred under an inert atmosphere and reflux conditions for 6–24 h. Upon completion of the reaction, the mixture was filtered under vacuum and the solid washed with glacial acetic acid (previously warmed up) and diethyl ether. The solid on filter was then triturated with water (5–10 mL), filtered under vacuum, and washed over filter with water to yield final compound **32** and intermediates **58** and **60**. In the case of **32**, the resulting solid was again triturated with 10 mL of an ethyl acetate/diethyl ether (1:1) mixture, filtered under vacuum, and washed over filter with petroleum ether to afford final compound **32** as a white solid.

##### 4.1.7.1 4-(4-Benzyl-5-hydroxy-3-methyl-1*H*-pyrazol-1-yl)benzoic Acid (MC4247, **32**)

^1^H-NMR (400 MHz; DMSO-*d*_6_) for the prevalent enol form^42,43^ δ 2.08 (s, 3H, C*H*_3_), 3.62 (s, 2H, C*H*_2_), 7.16 (t, 1H, *J* = 6.8 Hz, C*H* phenyl proton), 7.22–7.29 (m, 4H, C*H* phenyl protons), 7.92 (d, 2H, *J* = 8.8 Hz, C*H* carboxyphenyl protons), 7.99 (d, 2H, *J* = 8.8 Hz, C*H* carboxyphenyl protons), 11.22 (br s, 1H, O*H*), 12.65 (br s, 1H, COO*H*). ^13^C-NMR (100 MHz, DMSO-*d*_6_) δ 13.1, 27.3, 106.7, 117.1 (2C), 125.8, 128.1 (2C), 128.3 (2C), 130.5 (2C), 137.1, 140.9, 149.8, 157.3, 166.9. MS (ESI) *m*/*z:* 307 [M – H]^−^.

##### 4.1.7.2 4-(3-Isopropyl-5-oxo-4,5-dihydro-1*H*-pyrazol-1-yl)benzoic Acid (**58**)

Obtained as the 5-hydroxypyrazole tautomer 4-(5-hydroxy-3-isopropyl-1*H*-pyrazol-1-yl)benzoic acid. ^1^H-NMR (400 MHz; DMSO-*d*_6_) δ 1.20 (d, 6H, *J* = 6.8 Hz, 2 × C*H*_3_), 2.76–2.83 (m, 1H, C*H*), 5.42 (s, 1H, C*H* pyrazole proton), 7.91 (d, 2H, *J* = 8.2 Hz, C*H* benzoic acid ring protons), 7.99 (d, 2H, *J* = 8.2 Hz, C*H* benzoic acid ring protons), 11.80 (br s, 1H, O*H*), 12.75 (br s, 1H, COO*H*).

##### 4.1.7.3 4-(3-Benzyl-5-oxo-4,5-dihydro-1*H*-pyrazol-1-yl)benzoic Acid (**60**)

Obtained as the 5-hydroxypyrazole tautomer 4-(3-benzyl-5-hydroxy-1*H*-pyrazol-1-yl)benzoic acid. ^1^H-NMR (400 MHz; DMSO-*d*_6_) δ 3.82 (s, 2H, C*H*_2_), 5.34 (s, 1H, C*H* pyrazole proton), 7.21–7.23 (m, 1H, C*H* phenyl proton), 7.30–7.33 (m, 4H, C*H* phenyl protons), 7.92 (d, 2H, *J* = 8.8 Hz, C*H* benzoic acid ring protons), 8.00 (d, 2H, *J* = 8.8 Hz, C*H* benzoic acid ring protons), 12.04 (br s, 1H, O*H*), 12.85 (br s, 1H, COO*H*).

### 4.2 Cloning and Protein Expression

KAT8 plasmid was obtained by inserting the gene fragment encoding the catalytic domain of KAT8 (amino acids 173–458) plus an *N*-terminal 6×His tag and the TEV protease cleavage site into a pET28b expression vector (Novagen) between NcoI and XhoI cloning sites, using an In-Fusion cloning kit (Clonetech). The plasmid was amplified by transforming it into *E. coli* Stellar Competent Cells (Takara), and the DNA sequence was verified by Sanger sequencing. The plasmid was transformed in *E. coli* BL21(DE3) (New England Biolabs). Cells were grown in LB media supplemented with 50 μg/mL kanamycin at 37 °C. When cultures reached optical density at 600 nm (OD600) of ∼0.6, isopropyl-β-d-1-thiogalactopyranoside (IPTG) was added at a final concentration of 0.5 mM and cultures were further grown for 18 h at 16 °C. Cells were collected by centrifugation at 5000×*g* for 10 min at 4 °C.

### 4.3 Protein Purification

Cell pellets were resuspended in buffer containing 250 mM NaCl, 20 mM HEPES (pH 7.5), EDTA-free protease inhibitor cocktail (Roche), and 10 μg/mL DNaseI. The cell suspension lysed via sonication, and insoluble material was pelleted by centrifugation at 20,000×*g* for 20 min at 4 °C. The supernatant was filtered before loading onto a 5 mL HisTrap-HP column (GE Healthcare) equilibrated in 300 mM NaCl, 20 mM HEPES (pH 7.5), 0.1% β-mercaptoethanol, and 20 mM imidazole. After the clarified supernatant was loaded, the column was initially washed with 50 mL of 300 mM NaCl, 20 mM HEPES (pH 7.5), 0.1% β-mercaptoethanol, and 20 mM imidazole, and washed again with 50 mL of 300 mM NaCl, 20 mM HEPES (pH 7.5), 0.1% β-mercaptoethanol, and 80 mM imidazole. The bound protein was eluted with 300 mM NaCl, 20 mM HEPES (pH 7.5), 0.1% β-mercaptoethanol, and 400 mM imidazole. Peak fractions were combined, incubated with TEV protease, and dialyzed overnight against 300 mM NaCl, 20 mM HEPES (pH 7.5), 1 mM DTT, and 5% glycerol. This solution was then loaded again onto a 5 mL HisTrap-HP column equilibrated with 300 mM NaCl, 20 mM HEPES (pH 7.5), 1 mM DTT, and 5% glycerol, and the flow through, containing untagged KAT8, was collected, while His-tagged TEV and the His-tagged cleaved peptide remained bound to the resin. The protein was then concentrated in an Amicon Ultra-15 concentrator unit (Millipore) with a molecular cutoff of 3 kDa to 3 mg/mL and loaded onto a Superdex 200 Increase 10/300 GL SEC column (GE Healthcare) pre-equilibrated with 200 mM NaCl, 20 mM HEPES (pH 7.5), 1 mM DTT, and 5% glycerol.

### 4.4 Radioactive KAT8 Inhibition Assay

KAT8 inhibition assays were performed using the previously purified KAT8 (173–458, 5 nM), radio-isotope-labeled [^3^H]Acetyl-CoA (Perkin Elmer, 3 μM) as the acetyl donor, and biotinylated H4 peptide (Sigma-Aldrich, 2.5 μM) as a substrate in a 100 mM NaCl, 40 mM Tris·HCl (pH 7.5), and 1 mM DTT assay buffer. Purified KAT8 was incubated with the relevant inhibitor (1% DMSO final) in a 2-fold serial dilution starting at 200 μM for 20 min at rt. Then, [^3^H]Ac-CoA and biotinylated peptide were added to a total assay volume of 50 μL and the reaction mixture was incubated at rt for 4 h. The reaction was stopped, and the mixture was transferred to a 96-well streptavidin scintillant coated FlashPlate (Perkin Elmer) and incubated for 60 min at rt. The FlashPlate was washed with 0.1% Tween-20, and the liquid scintillation counting (MicroBeta Microplate Counter, Perkin Elmer) was employed to measure the radioactive signal. Experiments were performed in biological triplicates. Signal reduction compared to control was plotted (as inhibition percentage) as a function of compound concentration and fitted by nonlinear regression (GraphPad Prism 8.0) finally yielding the IC_50_ values.

### 4.5 HPLC-Based Analysis of Inhibitors’ Thiol Reactivity

Compounds **19** and **34** were dissolved in a solution containing 100 mM NaCl and 40 mM Tris·HCl (pH 7.5) with the addition of DTT, BME, or GSH (each at 2 mM) to a final concentration of 0.5 mM and incubated for 1, 8, and 24 h at rt. HPLC analysis was then performed at three wavelengths (254, 286, and 395 nm) under the same conditions described in the “Chemistry” paragraph of the Experimental Section.

### 4.6 Analysis of KAT8 Mode of Inhibition by **19** and **34**

The preincubation assay was performed by incubating KAT8 with the relevant inhibitor (12.5 μM final concentration) for 5, 10, 20, 30, 60, and 120 min at rt. The activity of KAT8 was then measured as described in the “Radioactive KAT8 inhibition assay” paragraph of the Experimental Section. The jump-dilution analysis was performed by incubating the previously purified KAT8 (500 nM) with the relevant inhibitor at a concentration equal to 10 × IC_50_ for 20 min at rt. The solution was then diluted 100× in the assay buffer containing [^3^H]Acetyl-CoA (3 μM) and biotinylated H4 peptide (2.5 μM) such that the enzyme concentration was 5 nM and the compound was at 0.1 × IC_50_. Fifty microliters were then incubated at rt for 10, 20, 30, 60, 120, and 240 min, and the mixtures were then processed as described in the “Radioactive KAT8 inhibition assay” paragraph of the Experimental section. Radioactive signal was normalized to the DMSO control reaction incubated for 240 min, converted in percent of KAT8 activity after 240 min, and plotted against each measurement time point. Experiments were performed in biological triplicates.

### 4.7 KAT2A, KAT2B, KAT3B, KAT5, KAT6A, KAT6B, and KAT7 Inhibition Assays

The effects of the compounds on other KATs were determined using the HotSpot KAT assay (Reaction Biology Corporation, Malvern, PA, USA) according to the supplier’s procedure. The recombinant catalytic domains of human KAT2A (aa 323–837, 15 nM), KAT2B (aa 492–658, 25 nM), KAT3B (aa 1284–1672, 1 nM), KAT6A (aa 488–778, 10 nM), KAT6B (aa 657–1069, 10 nM), and full length human KAT5 (25 nM) and KAT7 (25 nM) were used. Each enzyme was incubated with the relevant histone peptide [H3 peptide for KAT2A, KAT2B, KAT3B, KAT6A, and KAT7 (2.5–5 μM, depending on the KAT isoform); H2A peptide for KAT5 (1.25 μM); H4 peptide for KAT6B (2.5 μM)] as a substrate and radio-isotope-labeled [^3^H]Acetyl-CoA (0.2–1.5 μM, depending on the KAT isoform) as an acetyl donor in the assay buffer [50 mM Tris–HCl (pH 8.0), 50 mM NaCl, 0.1 mM EDTA, 1 mM DTT, 0.1 mM PMSF, 1% DMSO] for 1 h at 30 °C in the presence or absence of various compounds (1% DMSO final) titrated in a 2-fold serial dilution starting at 200 μM. Histone peptide acetylation was assessed by liquid scintillation counting using a Tri-Carb 2800TR Liquid Scintillation Analyzer (Perkin Elmer). Experiments were performed in biological triplicates. Signal reduction compared to control was plotted (as inhibition percentage) as a function of compound concentration. In cases in which an IC_50_ was calculated, the values were fitted by nonlinear regression (GraphPad Prism 8.0).

### 4.8 KDACs Inhibition Assays

KDAC1, 2, 3 6, and 8 inhibition assays were performed as described previously.^52^ Briefly, the relevant human recombinant KDAC was diluted in an assay buffer with the following composition: 150 mM NaCl, 25 mM Tris–HCl (pH 8.0), 2.7 mM KCl, and 1 mM MgCl_2_; BSA (0.1 mg/mL) was also added in the case of KDAC1. Forty microliters of this solution were added to 10 μL of each inhibitor (200 μM final concentration in 1% DMSO final) and 50 μL of KDAC Assay substrate (Boc-Lys-(Ac)-AMC, 7–100 μM final concentration, depending on the KDAC isoform), and the resulting solution was mixed. The plate was covered and incubated at rt for 45 min, and then 50 μL of the KDAC Stop Solution (SAHA, 100 μM final concentration + trypsin, 2 mg/mL final concentration) was added in all wells. After thorough mixing of the resulting solution, the plate was covered and incubated at 37 °C for 25 min and the fluorescence (λ_emission_ = 460 nm; λ_excitation_ = 390 nm) was measured on a EnSight Multimode Plate Reader (Perkin Elmer). The shown values are average of three technical replicates ± SD.

### 4.9 SPR-Based KAT8 Binding Experiments

SPR analyses were performed on a Biacore 3000 optical biosensor (GE Healthcare) equipped with a research-grade CM5 sensor chip. KAT8 was immobilized (30 μg/mL in 10 mM sodium acetate, pH 4.5) at a flow rate of 10 μL/min by using a standard amine-coupling protocol to obtain a density of 12 kRU. One flow cell was left empty for background subtractions. Both compounds, dissolved in DMSO (100%), were diluted in assay buffer (150 mM NaCl, 10 mM HEPES pH 7.4, 0.005% Tween-20), always maintaining a final 1% DMSO concentration. Binding experiments were performed at 25 °C by using a flow rate of 30 μL/min, with 120 s monitoring of association and 200 s monitoring of dissociation. Regeneration of the surfaces was performed, when necessary, by a 10 s injection of 1 mM NaOH. The simple 1:1 binding model of the BIAevaluation software was used for determining equilibrium dissociation constant (*K*_D_) and kinetic dissociation (*k*_d_) and association (*k*_a_) constants by using eqs 1 and 2:
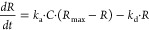
1where *R* represents the response unit and *C* is the concentration of the analyte.
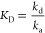
2

### 4.10 Molecular Docking

In this analysis, the following crystal structures were retrieved from Protein Data Bank (PDB; https://www.rcsb.org/): 4PZS - KAT3B histone acetyltransferase domain in complex with acetyl-CoA;^53^ 5KJ2 - KAT3B histone acetyltransferase in complex with the inhibitor A-485;^45^ 6BA4 - MYST acetyltransferase domain in complex with acetyl-CoA cofactor;^54^6PDB - MYST acetyltransferase domain in complex with inhibitor 80;^46^ and 4NSQ: KAT2B histone acetyltransferase in complex with CoA.^55^ Marvin Sketch (https://chemaxon.com/products/marvin) was used to draw the different compounds. OBabel (https://openbabel.org/) was used for the interconversion between file formats, for the energy evaluation and minimization of molecules. In this analysis, the default General Amber Force Field (GAFF) has been used to minimize the inhibitors and to obtain energy-minimized structures. Molegro Virtual Docker (MVD; http://molexus.io/molegro-virtual-docker)^56^ has been used to perform both normal and template-based dockings of the inhibitors with default parameters, except for the “Grid resolution (Å)” parameter set to 0.20 and the “Max population size” parameter increased to 150. The same procedure was used for the template-based docking of the inhibitors of each class, using as template the highest-affinity representative compound (docked without restraints). In addition, MVD has been used to optimize the ligand pose obtained after the docking simulations. This optimization is possible through the energy minimization of the ligand (Tools > Ligand Energy Inspector > Action > Minimize Ligand) and of the active site residues (Tools > Sidechain Minimization). The docking parameters used for all the simulations were benchmarked for their ability to reproduce the conformation of several experimentally validated cocrystalized inhibitors of KATs: 4PZS, crystal structure of p300 histone acetyltransferase domain in complex with acetyl-coenzyme A;^53^ 5KJ2, p300/CBP inhibitor A-485 complex;^45^ 6BA4, KAT8 acetyltransferase domain in complex with acetyl-CoA;^54^ 6CT2, MYST histone acetyltransferase KAT6A/B in complex with WM-1119;^54^ 6PDB, KAT6A in complex with inhibitor 8040;^46^ and 7SZQ, p300/CBP in complex with the azaindazole inhibitor ETL^57^ (Figure S4).

### 4.11 MD Simulations

Setup of MD simulations, trajectory analysis, and visualization was carried out on a Linux workstation with an Intel Core i9-9820X 3.3 GHz processor. All MD simulations were run on a dual GPU cluster equipped with two NVIDIA GeForce RTX 2080. Protein structure preparation and analysis were carried out using PyMod 3.^58^ All structures were preprocessed to assign alternates to the highest occupancy conformation, add hydrogens and missing loops using homology modeling, and fix discrepancies between sequence and structure. The protonation state of titratable residues at pH 7.0 was manually checked for consistency. Each system’s nonprotein and nonligand atoms were completely removed before the structure was saved for simulation, except for the structural water molecules 4.5 Å from the ligand, which were considered during the simulations. The general Amber force field^59^ was used to parameterize the ligands, whereas the ff14SB force field^60^ was used to parametrize the protein atoms. The AM1-BCC forcefield was used to assign the ligand appropriate partial charges.^61^ Using the TIP3P^62^ model for water molecules, each system under investigation was solvated in a cubic box with a padding of 15 Å. To neutralize the system and reach a salt content of 0.154 M, the appropriate number of sodium and chloride ions was introduced. Each system was energy reduced with the conjugate-gradient approach for a total of 5000 steps prior to MD simulations to eliminate collisions and undesirable interactions. Afterward, each minimized complex was subjected to an equilibration protocol consisting of a total of 5 ns of in the canonical ensemble (NVT) and isothermal–isobaric ensemble (NPT), with harmonic positional restraints (5 kcal·mol^–1^·Å^–2^ force constant) applied on both protein and ligand atoms. MD simulations at the production stage were performed for 100 ns using an integration timestep of 4 fs, keeping the temperature at a constant value of 310 K, with the ACEMD 3^63^ engine, which is based on OpenMM 7.^64^

### 4.12 Cell Cultures and Reagents

Human commercially available AML U937, breast cancer MCF7, lung adenocarcinoma NCI-H460, H1299 and A-549, glioblastoma U251, B lymphocyte AHH1 cell lines were maintained in compete 10% foetal bovine serum (FBS,) RPMI 1640 (Euroclone, Milan, IT) medium. Human commercially available colon cancer HCT116 and HT29 lines and cervix adenocarcinoma HeLa cells were maintained in compete 10% FBS Dulbecco’s Modified Eagle Medium (DMEM) high glucose medium (Euroclone). l-Glutamine, FBS, and penicillin streptomycin mixture added to medium were obtained from Euroclone. All cell lines were cultured at 37 °C in a humidified atmosphere of 5% CO_2_:95% air and were tested for mycoplasma contamination routinely by RT-PCR.

### 4.13 Target Engagement Analysis

Target engagement in cells was determined by CETSA.^65,66^ For CETSA experiments with cells treated in culture, ∼1 × 10^6^ HT29 were treated with DMSO (0.5% *v*/*v* final) or 100 μM compound for 4 h at 37 °C and 5% CO_2_ in a humidified incubator. The cells were harvested, washed twice with PBS, and resuspended in PBS with protease inhibitors. The cell suspensions were then distributed into PCR tubes, heated at the indicated temperature in a SimpliAmp Thermal Cycler (Applied Biosystems) for 3 min, and then cooled for another 3 min at rt. The cell suspensions were then lysed by freeze-thawing three times with 3 min incubations in an ethanol/dry ice bath and water bath at 37 °C. The lysates were then centrifuged at 20,000×*g* for 30 min at 4 °C to remove cellular debris and protein aggregates, and lysates were prepared for WB analysis**.** Proteins were separated by SDS-PAGE on 13.5% gels and were transferred to nitrocellulose membranes (Biorad Laboratories). Membranes were blocked for 1 h and incubated overnight with the appropriate primary antibody at 4 °C. After incubation, membranes were washed and incubated with the appropriate secondary antibody (Biorad Laboratories) for 1 h at room temperature. Following primary rabbit monoclonal antibodies KAT8 (1:1000, D5T3R Rabbit mAb #46862 Cell Signalling Technology), and SOD1 (1:1000, Rabbit mAb #2770, Cell Signalling Technology). Detection was performed by an ECL Western Blotting Substrate (Biorad Laboratories). Images of blots were acquired through the ChemiDoc Imaging Systems (Biorad Laboratories). The band signals were quantified using ImageJ and values normalized to the 37 °C DMSO control.

### 4.14 IF and WB Analyses

For IF analysis, 1 × 10^5^ HT-29 cells were grown on sterile coverslips. After 24 h, cells were exposed to different compounds for 24 h. IF staining for different acetylated lysine residues of histone H3 (H3K27Ac) and H4 (H4K16Ac) were carried out on PFA-fixed cells blocked with 20% goat serum in PBS and incubated overnight at 4 °C with primary rabbit monoclonal antibodies histone H3K27Ac (D5E4, Rabbit mAb#8173) and H4K16Ac (E2B8W, Rabbit mAb#13534, Cell Signalling Technology). Primary antibodies were detected using rabbit anti-IgG secondary antibodies conjugated with Cy3 (Jackson Immunoresearch 711-165-152). DNA was stained using 0.05 μg/mL 4′,6-diamidino-2-phenylindole (DAPI). The samples were acquired using an Olympus AX70 (Olympus America Inc.) microscope. Serial *Z* stacks of 0.4 μm thickness were acquired, taking care to cover the entire cell volume. For quantification of fluorescence intensity signals, images of untreated or treated cells were analyzed and processed using opensource Cell Profiler 4.1.3 image analysis software (https://cellprofiler.org/) to measure fluorescence-integrated intensity values for antibody binding relating to individual cells as previously reported.^67^

For WB experiments, the cells were lysed in RIPA buffer supplemented with 1× SIGMAFAST protease inhibitor cocktail (Sigma-Aldrich, S8830); 20 μg of total protein was resolved in 13.5% gels by SDS-PAGE. Then, the proteins were transferred to nitrocellulose membranes (Biorad Laboratories). Membranes were blocked for 1 h and incubated overnight with the appropriate primary antibody at 4 °C. After incubation, membranes were washed and incubated with the appropriate secondary antibody (Biorad Laboratories) for 1 h at room temperature. Following primary rabbit monoclonal antibodies H4K16Ac (1:1000, E2B8W, Rabbit mAb#13534, Cell Signalling Technology), KAT8 (1:1000, D5T3R Rabbit mAb #46862 Cell Signalling Technology), GAPDH (1:1000, sc-32233, Santa Cruz Biotechnologies), anti-LC3B (Sigma-Aldrich, L7543), anti-SQSTM1/p62 (Santa Cruz Biotechnology, sc-28,359), and anti-GAPDH (Santa Cruz Biotechnology, sc-32,233). Secondary antibodies: anti-mouse (Bio-Rad Laboratories, 1,706,515) or anti-rabbit (Bio-Rad Laboratories, 1,706,516) IgG-horseradish peroxidase-conjugated antibodies. Detection was performed by ECL Western Blotting Substrate (Biorad Laboratories). Images of blots were acquired through the ChemiDoc Imaging Systems (Biorad Laboratories). The band signals were quantified using ImageJ, and values were normalized to relative controls, depending on the analysis.

### 4.15 Analysis of Cell Proliferation

For analysis of cell proliferation, cells were plated in sextuplicate on 96-well plates in complete media. After 24 h, cells were exposed to different drugs for 72 h. The inhibitory effect of different drugs was assessed by MTT (Sigma, St. Louis, MO, USA) colorimetric assays as previously reported.^67^ Aqueous DMSO (0.5% *v*/*v*) was used as a control (untreated). The results are reported as “viability of drug-treated cells/viability of untreated cells” × 100 and represent the average ±SD of three independent experiments. IC_50_ values were using fitted by nonlinear regression using GraphPad Prism 8.0.

### 4.16 Flow Cytometry

For the analysis of the progression of cells through cell cycle phases and sub-G1 peak identification, 24 h after plating, cells were treated with the different compounds. After 72 h, both floating and adherent cells were collected and fixed with cold 70% ethanol. At least 24 h after fixation, cells were incubated with 0.025 mg/mL Propidium Iodide for 15 min at room temperature and analyzed. For Annexin V analysis, 24 h after plating, cells were treated with 50 and 100 μM of the compounds. In the case of chloroquine experiments, 10 μM chloroquine (Sigma-Aldrich, A9165) was added 30 min before compounds. 72 h after treatment, both floating and adherent cells were collected and stained with an Annexin V-FITC apoptosis detection kit (ENZO #ALX-850-020-KI). Samples were incubated 15 min in the dark at rt, and 10,000 events were then analyzed.

### 4.17 Quantitative Real Time PCR

Total RNA was isolated from cells after 48 h of treatment with compounds **19** and **34** (100 μM) using TRIzol reagent (Life Technologies) following the manufacturer’s instructions. cDNA was obtained by reverse transcription using a SensiFast cDNA Synthesis kit (Meridian Bioscience). qRT-PCR was performed using a Luna Universal qPCR Master Mix (BioLabs) and detected by Quant Studio3. The fold change of gene expression data was calculated by using the ddCT method. All samples were run in sextupled, and the results were presented as mean ± SEM. Detailed sequence of the primers used in the experiments can be found in the list below.

**UCP2—**Forward: 5′-GCAACCCAGCTCAAGGTCAG-3′
Reverse 5′-CTGAGAAGGCTCAGGCAAATG-3′
**HOXA9 —** Forward: 5′-AGGCGCCTTCTCTGAAAACA-3′
Reverse: 5′-GGCTGCTGGGTTATTGGGAT-3′

### 4.18 Fluorescence Microscopy in EGFP-LC3 H1299 Cells and Analysis

The stable EGFP-LC3 H1299 cell line was used to detect autophagic structures by fluorescence spinning disk microscopy. Cells were grown on glass coverslips, treated with compounds for 48 h and fixed in 3.7% formaldehyde (Sigma, F1635) DNA was counterstained with 0.05 μg/mL DAPI (4,6-diamidino-2-phenylindole; Sigma-Aldrich, D9542). At least 500 cells were counted for each experimental point, and cells showing ≥15 dots were considered positive. Dots were counted using a pipeline created in a previous work on the CellProfiler software.^68^ Data are presented as median with interquartile range. Samples were observed under a Nikon Eclipse Ti2 microscope with a 60× (1.4 NA) objective, equipped with a CrestOptics X-Light V3 confocal spinning disk and a back illuminated Kinetix sCMOS camera.

## Supporting Information Available

Melting point, recrystallization system, and yield data; elemental analyses for final compounds; inhibitory activities of compounds and SAHA against KDAC1-3, 6, and 8; HPLC traces for compounds **19**, **34**, and **39**; HPLC traces of compounds **19** and **34** in the presence of DTT, BME, or GSH; preincubation and jump-dilution assays; examples of KAT/inhibitor complexes obtained from docking, compared to experimentally determined structures from the PDB; molecular docking results for C646 and compound**2**; RMSD plots of the MD simulation of **19** and **34**; predicted binding mode of compound **26** to KAT8; KAT8 aggregation temperature CETSA experiments for compounds **19** and **34**; antiproliferative activities of compounds **19** and **34**; and representative dot plot of dot plot of Annexin V-FITC fluorescence upon treatment of HCT116 cells with **19** and **34** alone or in combination with CQ (PDF)
MD simulation of **19** in complex with KAT8 (Supplementary Movie 1) (MP4)
MD simulation of **34** in complex with KAT8 (Supplementary Movie 2) (MP4)
Molecular formula strings with associated biological data (CSV)

## Abbreviations

AA: anacardic acid
Ac-CoA: acetyl coenzyme A
AML: acute myeloid leukemia
BME: β-mercaptoethanol
CQ: chloroquine
CRC: colorectal carcinoma
DAPI: 4′,6-diamidino-2-phenylindole
DMEM: Dulbecco’s modified eagle medium
FBS: foetal bovine serum
GAFF: general AMBER force field
GSH: reduced glutathione
HCC: hepatocellular carcinoma
IF: immunofluorescence
IPTG: isopropyl-β-d-1-thiogalactopyranoside
KAT: lysine acetyltransferase
KAT8i: KAT8 inhibitors
KDAC: lysine deacetylase
MD: molecular dynamics
MSL: male-specific lethal
MTT: 3-(4,5-dimethylthiazol-2-yl)-2,5-diphenyltetrazolium bromide
MVD: Molegro Virtual Docker
NSCLC: non-small cell lung cancer
NSL: nonspecific lethal
OTSCC: oral tongue squamous cell carcinoma
PTMs: post-translational modifications
PyBOP: benzotriazol-1-yloxytripyrrolidinophosphonium hexafluorophosphate
SPR: surface plasmon resonance
WB: Western blot
