# Supplementary material for: First-in-Class Selective Inhibitors of the Lysine Acetyltransferase KAT8
Source: J Med Chem. 2023 May 8;66(10):6591–616. doi: 10.1021/acs.jmedchem.2c01937 (PMC10226132; doi:10.1021/acs.jmedchem.2c01937)
Supplement: Supplementary file 1 — jm2c01937_si_001.pdf [file jm2c01937_si_001.pdf]

SUPPORTING INFORMATION

First-in-Class Selective Inhibitors of the Lysine Acetyltransferase KAT8

Francesco Fiorentino,[a] Sara Sementilli,[b] Martina Menna,[a] Federica Turrisi,[b] Stefano Tomassi,[c] Francesca Romana Pellegrini,[b] Angela Iuzzolino,[b] Francesca D'Acunzo,[d] Alessandra Feoli,[e] Hannah Wapenaar,[f] Sophie Taraglio,[g] Caterina Fraschetti,[a] Donatella Del Bufalo,[h] Gianluca Sbardella,[e] Frank J. Dekker,[f] Alessandro Paiardini,[g] Daniela Trisciuoglio,[b]* Antonello Mai,[a,i]* and Dante Rotili[a,]*

[a]*Department of Drug Chemistry and Technologies, Sapienza University of Rome, P.le A. Moro 5, 00185 Rome, Italy;*

[b]*Institute of Molecular Biology and Pathology, National Research Council (CNR), Via degli Apuli 4, 00185 Rome, Italy;*

[c]*Department of Pharmacy, University of Naples "Federico II", via Domenico Montesano 49, 80131 Naples, Italy;*

[d]*Institute of Biological Systems (ISB), Italian National Research Council (CNR), Sezione Meccanismi di Reazione, c/o Department of Chemistry, Sapienza University of Rome, P. le A. Moro 5, Rome, 00185, Italy;*

[e]*Department of Pharmacy, University of Salerno, via Giovanni Paolo II 132, 84084 Fisciano (SA), Italy;*

[f]*Department of Chemical and Pharmaceutical Biology, University of Groningen, Antonius Deusinglaan 1, 9713 AV, Groningen, The Netherlands;*

[g]*Department of Biochemical Sciences, Sapienza University of Rome, P.le A. Moro 5, 00185 Rome, Italy;*

[h]*Preclinical Models and New Therapeutic Agents Unit, IRCCS-Regina Elena National Cancer Institute,*

Via Elio Chianesi 53, 00144 Rome, Italy;

[i]*Pasteur Institute, Cenci-Bolognetti Foundation, Sapienza University of Rome, P.le A. Moro 5, 00185 Rome, Italy.*

* Corresponding Authors

*For D.R.: Phone, +39-0649913237; Fax: +39 06 49693268; E-mail: dante.rotili@uniroma1.it

*For A.M.: Phone, +39 06 49913392; Fax, +39 06 49693268; E-mail, antonello.mai@uniroma1.it

*For D.T.: Phone, +39 06 49917515; E-mail, daniela.trisciuoglio@uniroma1.it

**Contents**	**Page(s):**
**Table S1.** Melting point, recrystallization system, and yield data for compounds **1-17** and **19-43**.	p. S4-S5
**Table S2.** Elemental analyses for final compounds **1-17** and **19-43.**	p. S6-S7
**Table S3.** Inhibitory activity of compounds **19**, **34**, **VII**, and SAHA against KDAC1-3, 6, and 8.	p. S8
**Purity control by HPLC of compounds 19, 34, and 39.**	p. S9
**Figure S1.** HPLC traces for compound **19**.	p. S10
**Figure S2.** HPLC traces for compound **34**.	p. S11
**Figure S3.** HPLC traces for compound **39**.	p. S12
**Figure S4.** HPLC traces of compound **19** in the presence of DTT, BME, or GSH.	p. S13
**Figure S5.** HPLC traces of compound **34** in the presence of DTT, BME, or GSH.	p. S14
**Figure S6.** Pre-incubation and jump-dilution assays performed on compounds **19** and **34**.	p. S15
**Figure S7.** Binding poses of KAT inhibitors or co-substrate Ac-CoA obtained from docking compared to experimentally determined KAT-inhibitor/co-substrate complexes structures from the PDB.	p. S16
**Analysis of C646 binding mode to KAT3B.**	p. S17
**Figure S8.** Molecular docking of C646 and compound **2** in the active sites of KAT3B and KAT8.	p. S17-S18
**Figure S9.** RMSD plots of the MD simulation (100 ns) of compounds **19** and **34**.	p. S18

**Figure S10.** Predicted binding mode of compounds **26** and **27** to KAT8.	p. S19
**Figure S11.** Predicted binding mode of compound **26** to KAT3B and focus on KAT2B active site.	p. S19
**Figure S12.** KAT8 aggregation temperature and target engagement of compounds **19** and **34** tested by CETSA in HT29 cells.	p. S20
**Figure S13.** Antiproliferative activities of KAT8i **19**, and **34** tested at 1.25, 2.5, 5, 10, 25, 50, 100, and 200 µM in HT29, HCT116, H1299, A549, and U937 cells for 72 h.	p. S21
**Figure S14.** WB analysis of autophagic marker expression levels in HeLa cells exposed to increasing doses of compounds **19** and **34** for 48 h.	p. S21
**Figure S15.** Representative dot plot of Annexin V-FITC fluorescence upon treatment of HCT116 cells with KAT8i **19** and **34** alone (100 µM) or in combination with autophagy inhibitor CQ (10 µM) for 72 h.	p. S22
**Supplementary Movies Captions.**	p. S22
**References**	p. S23

Table S1. Melting point, recrystallization system, and yield data for compounds **1-17** and **19-43**.

Lab Code	Compd	Mp (°C)	Recryst. system[a]	Yield (%)
MC3983	**1**	>300	A	77
MC3637	**2**	>300	A	70
MC4039	**3**	>300	A	70
MC4052	**4**	>300	A	67
MC3987	**5**	>300	A	64
MC4008	**6**	>300	A	83
MC4049	**7**	>300	A	75
MC3996	**8**	296-297	A	75
MC4023	**9**	>300	A	67
MC3991	**10**	>300	A	73
MC4028	**11**	279-280	A	58
MC4006	**12**	>300	A	79
MC3989	**13**	>300	A	69
MC4020	**14**	295-296	A	60
MC4248	**15**	>300	A	48
MC4050	**16**	206-207	A	86
MC4010	**17**	231-232	A	84
MC4033	**19**	294- 295	A	54
MC4155	**20**	257-259	A	50
MC4174	**21**	278-280	A	54
MC4156	**22**	259-262	A	52
MC4276	**23**	266-268	A	57
MC4283	**24**	>300	A	68
MC4241	**25**	>300	A	64

MC4215	**26**	272-275	A	52
MC4282	**27**	>300	A	56
MC4217	**28**	262-264	A	51
MC4274	**29**	235-236	A	63
MC4184	**30**	261-264	A	53
MC4264	**31**	158-160	B	60
MC4247	**32**	252-256	A	58
MC4170	**33**	251-254	A	81
MC4171	**34**	270-273	A	69
MC4280	**35**	228-229	A	56
MC4284	**36**	273-275	A	55
MC4281	**37**	256-258	A	64
MC4273	**38**	139-141	B	52
MC4270	**39**	211-213	A	71
MC4278	**40**	246-248	A	63
MC4277	**41**	170-172	C	53
MC4289	**42**	251-252	A	52
MC4285	**43**	260-261	A	57

[a]A: ethanol; B: acetonitrile; C: methanol.

Table S2. Elemental analyses for final compounds **1-17** and **19-43**.

Lab Code	Compd	Formula	MW	Calculated, %					Found, %				
				C	H	N	S	F/Cl	C	H	N	S	F/Cl
MC3983	**1**	$C_{23}H_{17}N_3O_4$	399.41	69.17	4.29	10.52			69.27	4.30	10.47		
MC3637	**2**	$C_{23}H_{16}N_4O_6$	444.40	62.16	3.63	12.61			62.28	3.63	12.55		
MC4039	**3**	$C_{23}H_{16}N_4O_6$	444.40	62.16	3.63	12.61			62.27	3.64	12.55		
MC4052	**4**	$C_{23}H_{16}N_4O_6$	444.40	62.16	3.63	12.61			62.29	3.64	12.54		
MC3987	**5**	$C_{23}H_{16}ClN_3O_4$	433.85	63.68	3.72	9.69		8.17	63.79	3.73	9.63		8.14
MC4008	**6**	$C_{23}H_{16}ClN_3O_4$	433.85	63.68	3.72	9.69		8.17	63.81	3.73	9.62		8.14
MC4049	**7**	$C_{23}H_{16}ClN_3O_4$	433.85	63.68	3.72	9.69		8.17	63.79	3.74	9.62		8.15
MC3996	**8**	$C_{23}H_{16}FN_3O_4$	417.40	66.18	3.86	10.07		4.55	66.30	3.87	10.03		4.52
MC4023	**9**	$C_{23}H_{16}FN_3O_4$	417.40	66.18	3.86	10.07		4.55	66.30	3.86	10.04		4.53
MC3991	**10**	$C_{24}H_{19}N_3O_5$	429.43	67.13	4.46	9.79			67.23	4.47	9.74		
MC4028	**11**	$C_{24}H_{19}N_3O_5$	429.43	67.13	4.46	9.79			67.24	4.46	9.74		
MC4006	**12**	$C_{23}H_{17}N_3O_5$	415.41	66.50	4.13	10.12			66.61	4.14	10.06		
MC3989	**13**	$C_{24}H_{19}N_3O_4$	413.43	69.72	4.63	10.16			69.85	4.63	10.10		
MC4020	**14**	$C_{24}H_{19}N_3O_4$	413.43	69.72	4.63	10.16			69.83	4.64	10.10		
MC4248	**15**	$C_{25}H_{20}N_4O_6$	472.46	63.56	4.27	11.86			63.67	4.28	11.80		
MC4050	**16**	$C_{22}H_{17}N_3O_2$	355.40	74.35	4.82	11.82			74.45	4.83	11.75		
MC4010	**17**	$C_{22}H_{16}N_4O_4$	400.39	66.00	4.03	13.99			66.11	4.04	13.93		
MC4033	**19**	$C_{16}H_{13}N_3O_3$	295.30	65.08	4.44	14.23			65.20	4.45	14.16		
MC4155	**20**	$C_{16}H_{12}N_2O_4$	296.28	64.86	4.08	9.46			64.98	4.09	9.41		
MC4174	**21**	$C_{16}H_{12}N_2O_3S$	312.34	61.53	3.87	8.97	10.26		61.64	3.88	8.93	10.24	
MC4156	**22**	$C_{18}H_{14}N_2O_3$	306.32	70.58	4.61	9.15			70.69	4.61	9.10		
MC4276	**23**	$C_{16}H_{13}N_3O_3$	295.30	65.08	4.44	14.23			65.19	4.44	14.17		
MC4283	**24**	$C_{15}H_{12}N_4O_3$	296.29	60.81	4.08	18.91			60.92	4.09	18.85		

MC4241	**25**	$C_{15}H_{12}N_4O_3$	296.29	60.81	4.08	18.91		60.93	4.08	18.84	
MC4215	**26**	$C_{20}H_{15}N_3O_3$	345.36	69.56	4.38	12.17		69.66	4.38	12.11	
MC4282	**27**	$C_{20}H_{15}N_3O_3$	345.36	69.56	4.38	12.17		69.65	4.39	12.11	
MC4217	**28**	$C_{17}H_{15}N_3O_3$	309.33	66.01	4.89	13.58		66.12	4.90	13.52	
MC4274	**29**	$C_{22}H_{17}N_3O_3$	371.40	71.15	4.61	11.31		71.26	4.62	11.25	
MC4184	**30**	$C_{17}H_{15}N_3O_3$	309.33	66.01	4.89	13.58		66.11	4.91	13.52	
MC4264	**31**	$C_{16}H_{15}N_3O_3$	297.31	64.64	5.09	14.13		64.75	5.10	14.07	
MC4247	**32**	$C_{18}H_{16}N_2O_3$	308.34	70.12	5.23	9.09		70.22	5.24	9.03	
MC4170	**33**	$C_{18}H_{17}N_3O_3$	323.35	66.86	5.30	13.00		66.97	5.31	12.93	
MC4171	**34**	$C_{21}H_{15}N_3O_3$	357.37	70.58	4.23	11.76		70.68	4.23	11.71	
MC4280	**35**	$C_{22}H_{17}N_3O_3$	371.40	71.15	4.61	11.31		71.25	4.62	11.26	
MC4284	**36**	$C_{16}H_{13}N_3O_3$	295.30	65.08	4.44	14.23		65.19	4.45	14.17	
MC4281	**37**	$C_{16}H_{14}N_4O_2$	294.31	65.30	4.79	19.04		65.40	4.80	18.97	
MC4273	**38**	$C_{18}H_{18}N_4O_2$	322.37	67.07	5.63	17.38		67.16	5.63	17.31	
MC4270	**39**	$C_{23}H_{20}N_4O_2$	384.44	71.86	5.24	14.57		71.95	5.25	14.51	
MC4278	**40**	$C_{18}H_{18}N_4O_3$	338.37	63.89	5.36	16.56		64.00	5.37	16.50	
MC4277	**41**	$C_{23}H_{27}N_5O_2$	405.50	68.13	6.71	17.27		68.23	6.71	17.20	
MC4289	**42**	$C_{15}H_{13}N_3O_4S$	331.35	54.37	3.95	12.68	9.68	54.50	3.96	12.64	9.65
MC4285	**43**	$C_{15}H_{14}N_4O_3S$	330.36	54.54	4.27	16.96	9.70	54.65	4.29	16.91	9.67

Table S3. Inhibitory activity of compounds **19**, **34**, **VII**, and SAHA against KDAC1-3, 6, and 8.[a]

**Compd**	***% Inhibition at 50 µM***					***% Inhibition at 100 µM***				
	**KDAC1**	**KDAC2**	**KDAC3**	**KDAC6**	**KDAC8**	**KDAC1**	**KDAC2**	**KDAC3**	**KDAC6**	**KDAC8**
**19**	0.03 ± 0.01 %	0.01 ± 0.02 %	0.5 ± 0.1 %	0.4 ± 0.08 %	0.02 ± 0.004 %	0.3 ± 0.05 %	0.7 ± 0.04 %	1.1 ± 0.09 %	1.3 ± 0.2 %	0.8 ± 0.1 %
**34**	0.04 ± 0.002 %	0.03 ± 0.004 %	0.02 ± 0.003 %	0.6 ± 0.2 %	0.04 ± 0.01 %	0.5 ± 0.2 %	0.4 ± 0.1 %	0.6 ± 0.07 %	2.1 ± 0.4 %	0.6 ± 0.08 %
**VII (C646)**	0.7 ± 0.1 %	75.4 ± 1.3 %	54.5 ± 1.0 %	87.2 ± 2.6 %	77.6 ± 1.9 %	1.3 ± 0.2 %	87.6 ± 1.2 %	86.2 ± 2.6 %	98.1 ± 1.8 %	89.1 ± 3.3 %
**SAHA**	100 ± 1.1 %	100 ± 2.2 %	100 ± 1.3 %	100 ± 0.9 %	98.9 ± 0.7 %	100 ± 0.5 %	100 ± 1.6 %	100 ± 0.9 %	100 ± 1.4 %	99.6 ± 2.1 %

[a] Values are means of three technical replicates ± standard deviation (SD).

Purity control by HPLC of compounds 19, 34, and 39.

The purity of compounds **19**, **34**, and **39** was analyzed by HPLC. The HPLC system consisted of a Dionex UltiMate 3000 UHPLC (Thermo Fisher) system equipped with an automatic injector, column heater and coupled with a Diode Array Detector DAD-3000 (Thermo Fisher). The analytical controls were performed on a Hypersil GOLD™ C18 Selectivity 5 μm (4.6 × 250 mm) HPLC Column (Thermo Fisher) in gradient elution. Eluents: A) H_2O/CH_3CN, 95/5 (*v/v*) + 0.1% TFA; B) CH_3CN/H_2O, 95/5 (*v/v*) + 0.1% TFA. A 20 min linear gradient elution from 30% to 100% solvent B was followed by 5 min at 100% B. The flow rate was 1.0 mL/min and the column was kept at a constant temperature of 30 °C. Samples were dissolved in solvent B at a concentration of 0.25 mg/mL and the injection volume was 10 μL.

By analysing the HPLC traces at 286 nm (wavelength of maximum absorbance for all compounds), a chemical purity > 96 % was recorded for all molecules. Specifically, the chemical purities were:

i) 97.91% for compound **19** (retention time 11.46 min);

ii) 98.08% for compound **34** (retention time 15.53 min);

iii) 99.64% for compound **39** (retention time 13.91 min).

In addition, chromatographic traces acquired at three wavelengths (254 nm, 286 nm, and 395 nm) for all compounds are reported in Figures S1, S2, and S3.

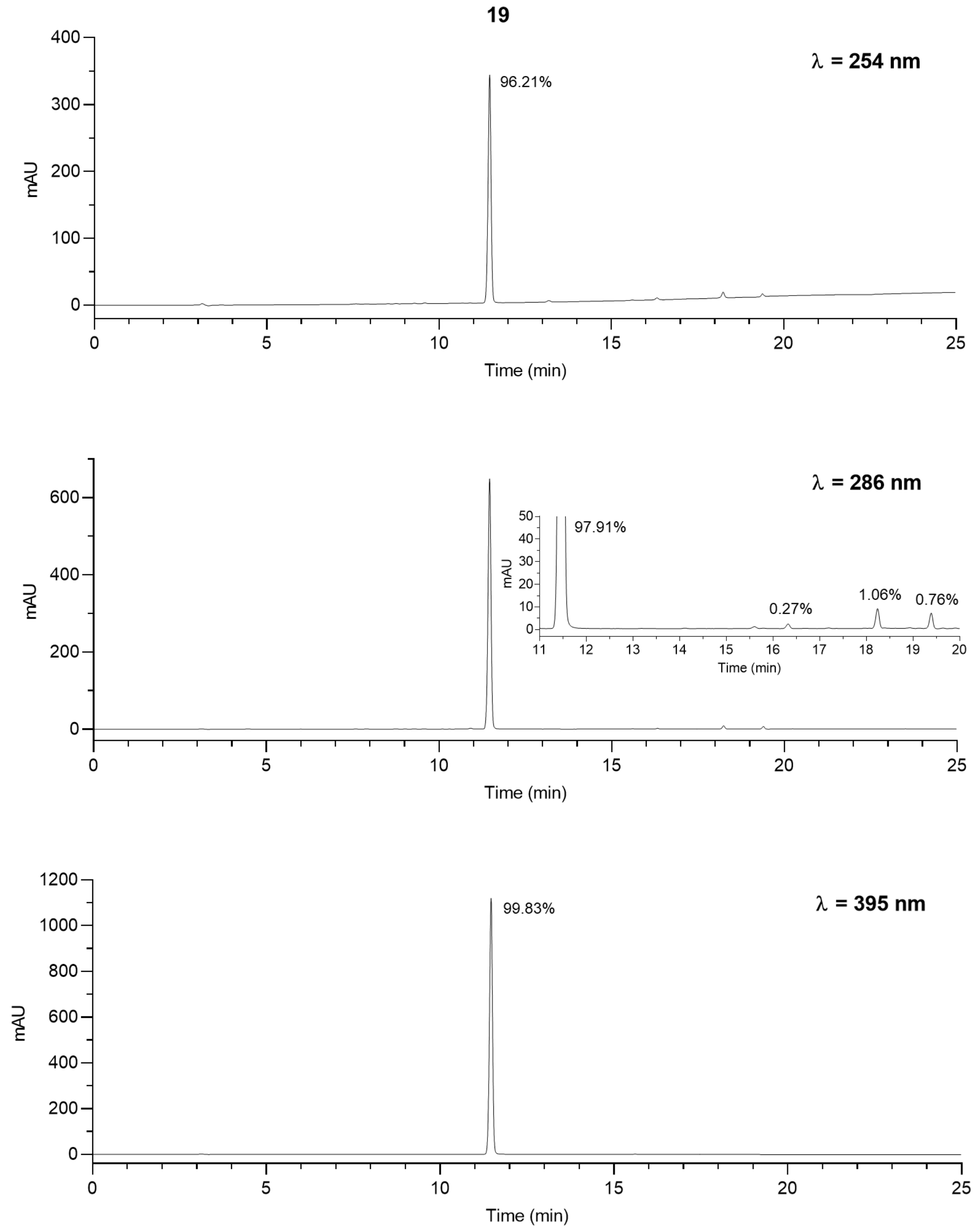

Figure S1. HPLC traces for compound **19**.

34

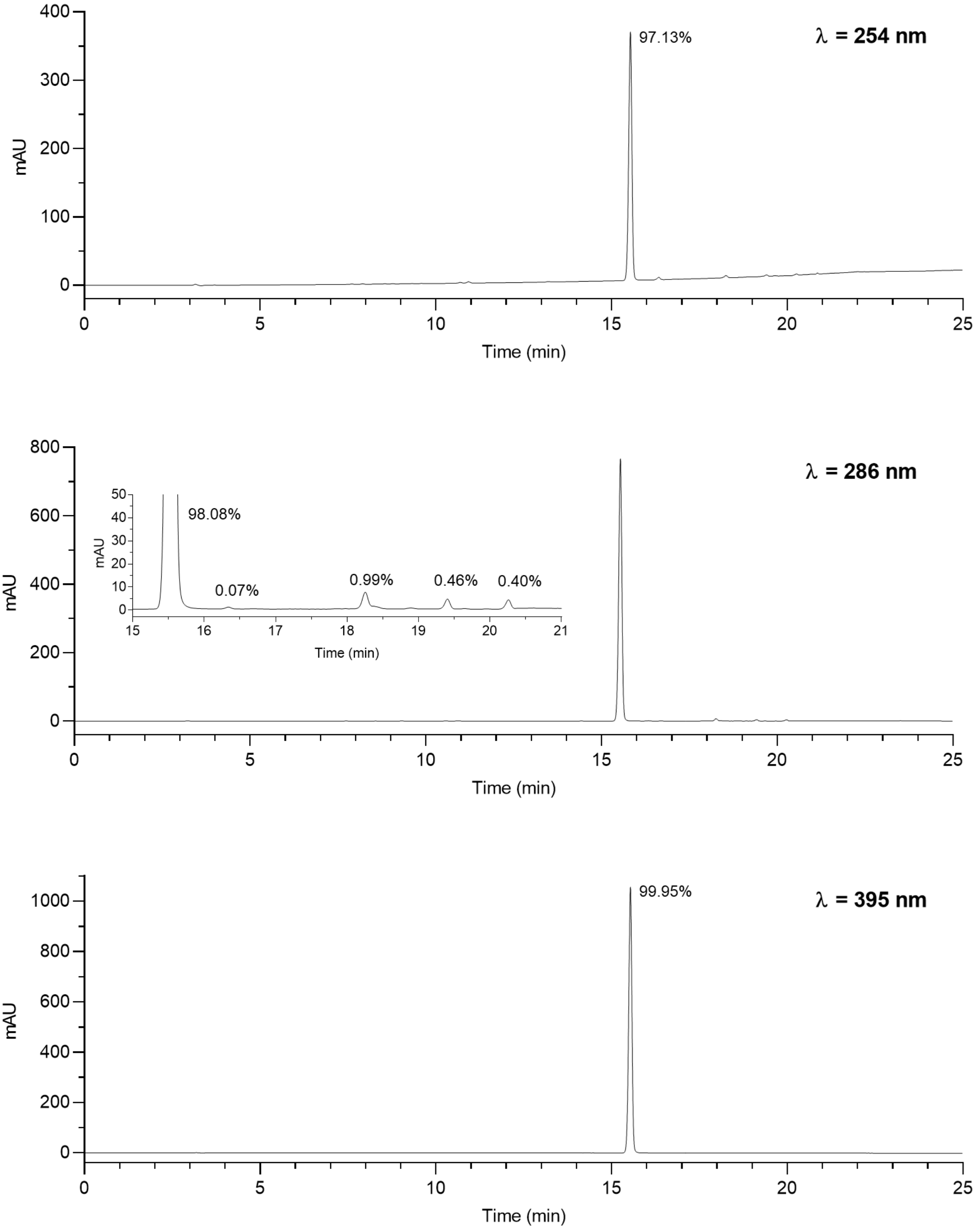

Figure S2. HPLC traces for compound **34**.

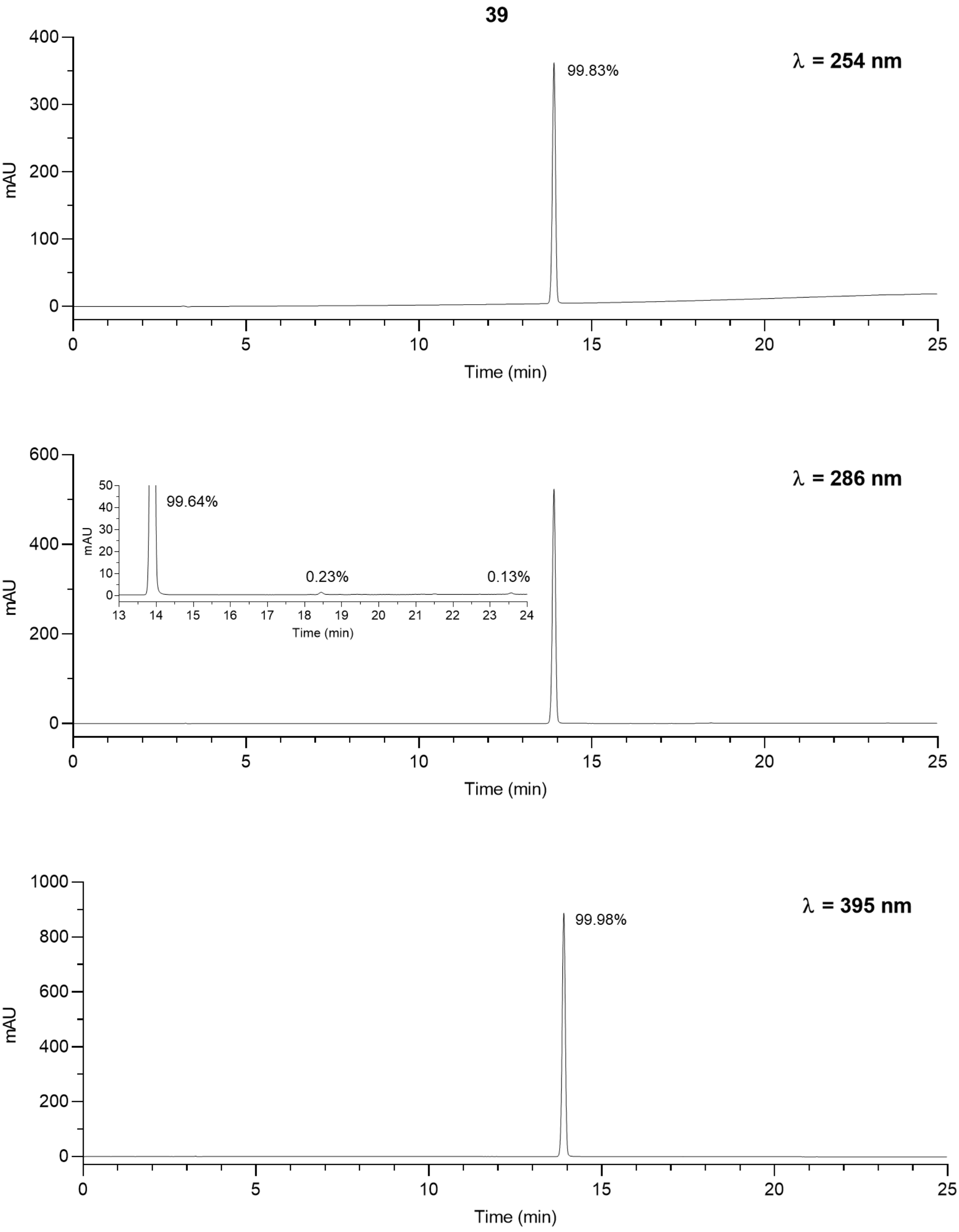

Figure S3. HPLC traces for compound **39**.

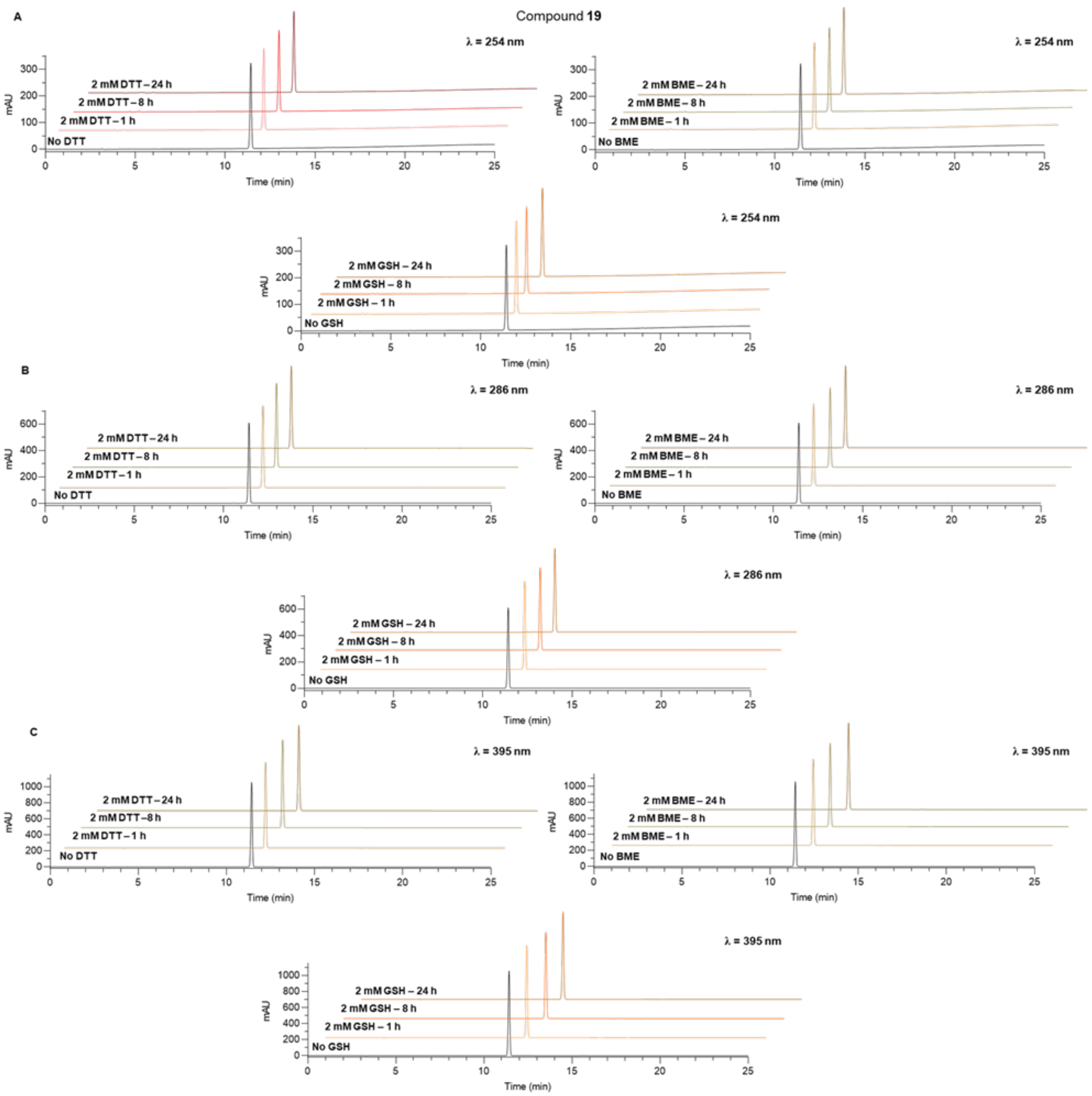

Figure S4. HPLC traces of compound **19** in the presence of DTT, BME, or GSH. We assessed the reactivity of **19** with thiol-containing nucleophiles in a solution containing 100 mM NaCl and 40 mM Tris·HCl (pH 7.5) with the addition of DTT, BME, or GSH (each at 2 mM). We dissolved the compound in the buffer (final concentration = 0.5 mM) with the relevant nucleophile, incubated the solution at rt and then performed analytical HPLC under the same conditions described in the Experimental Section. HPLC runs were performed after 1, 8, and 24 h of incubation and acquired at three wavelengths: 254 nm (A), 286 nm (B), and 395 nm (C).

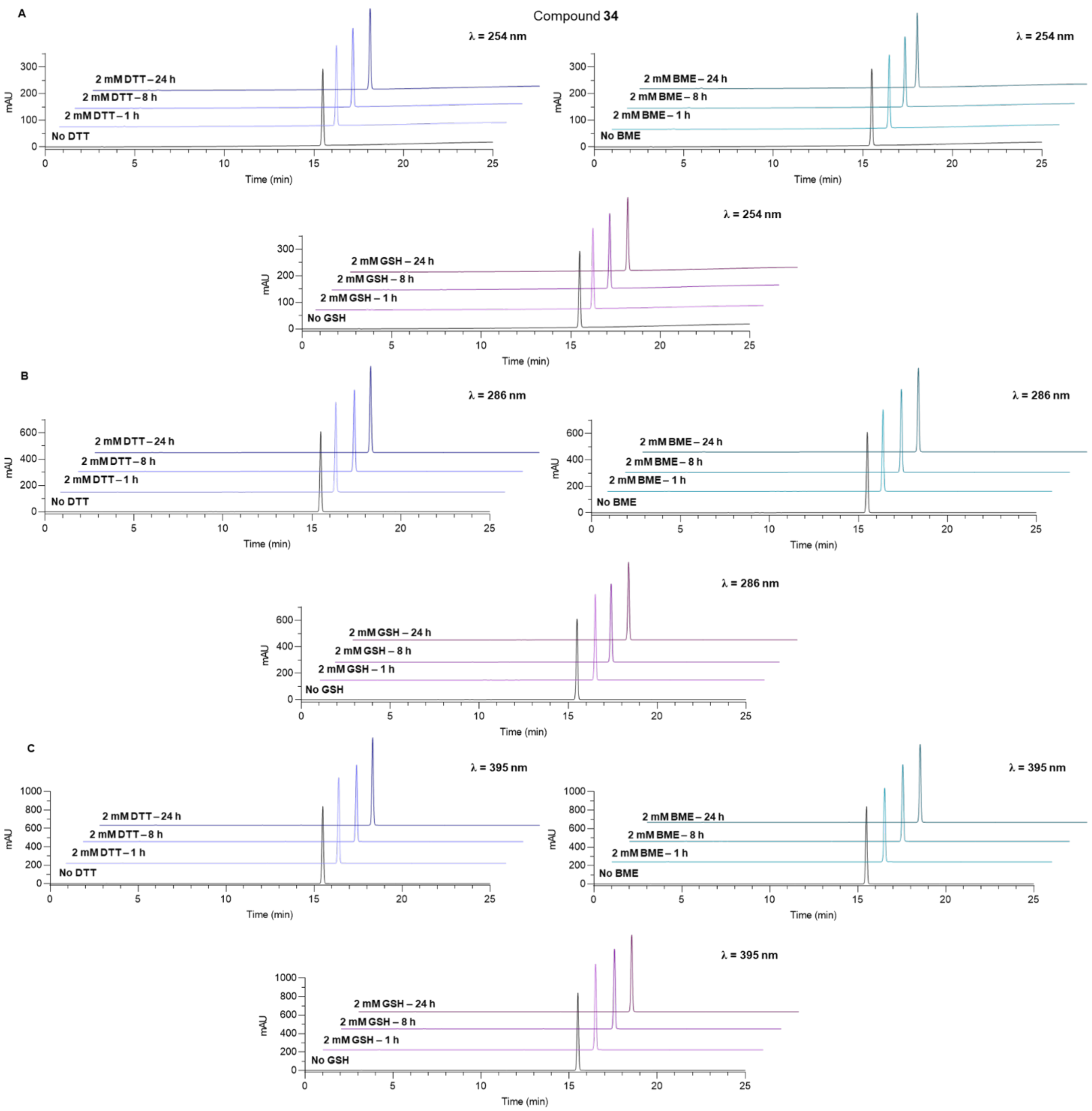

Figure S5. HPLC traces of compound **34** in the presence of DTT, BME, or GSH. We assessed the reactivity of **34** with thiol-containing nucleophiles in a solution containing 100 mM NaCl and 40 mM Tris·HCl (pH 7.5) with the addition of DTT, BME, or GSH (each at 2 mM). We dissolved the compound in the buffer (final concentration = 0.5 mM) with the relevant nucleophile, incubated the solution at rt and then performed analytical HPLC under the same conditions described in the Experimental Section. HPLC runs were performed after 1, 8, and 24 h of incubation and acquired at three wavelengths: 254 nm (A), 286 nm (B), and 395 nm (C).

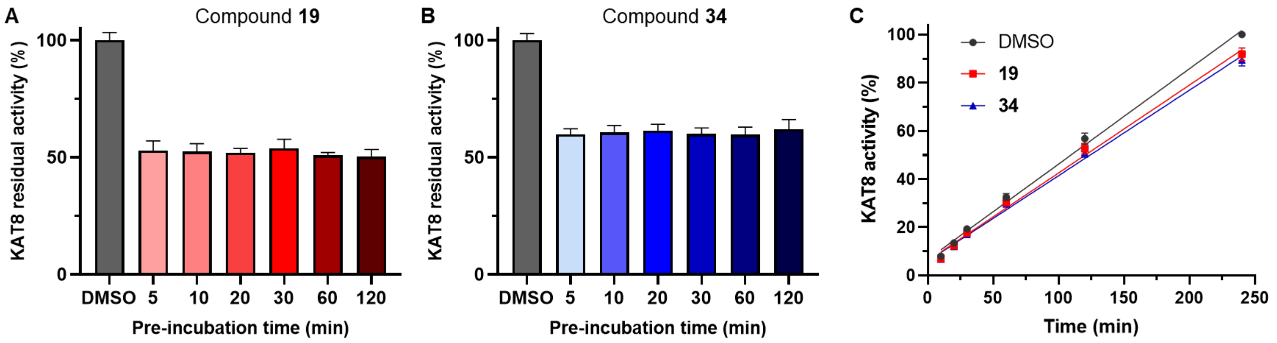

Figure S6. Pre-incubation assays performed on compounds **19** (A) and **34** (B). Each compound (at a final concentration of 12.5 μM) was pre-incubated with KAT8 for 5, 10, 20, 30, 60, and 120 min before adding the H4 peptide and Ac-CoA substrates and initiating the enzyme reaction. (C) Jump-dilution experiment performed on **19** and **34**. Following a 30 min exposure of 0.5 μM KAT8 to each compound at a concentration equal to 10x their IC_{50} (121 μM for **19** and 81 μM for **34**), the mixtures were diluted 100-fold and KAT8 activity was measured after 10, 20, 30, 60, 120, and 240 min. KAT8 activity is indicated as % of the activity measured after 240 min of incubation (assay inhibition condition) in the presence of the only DMSO control.

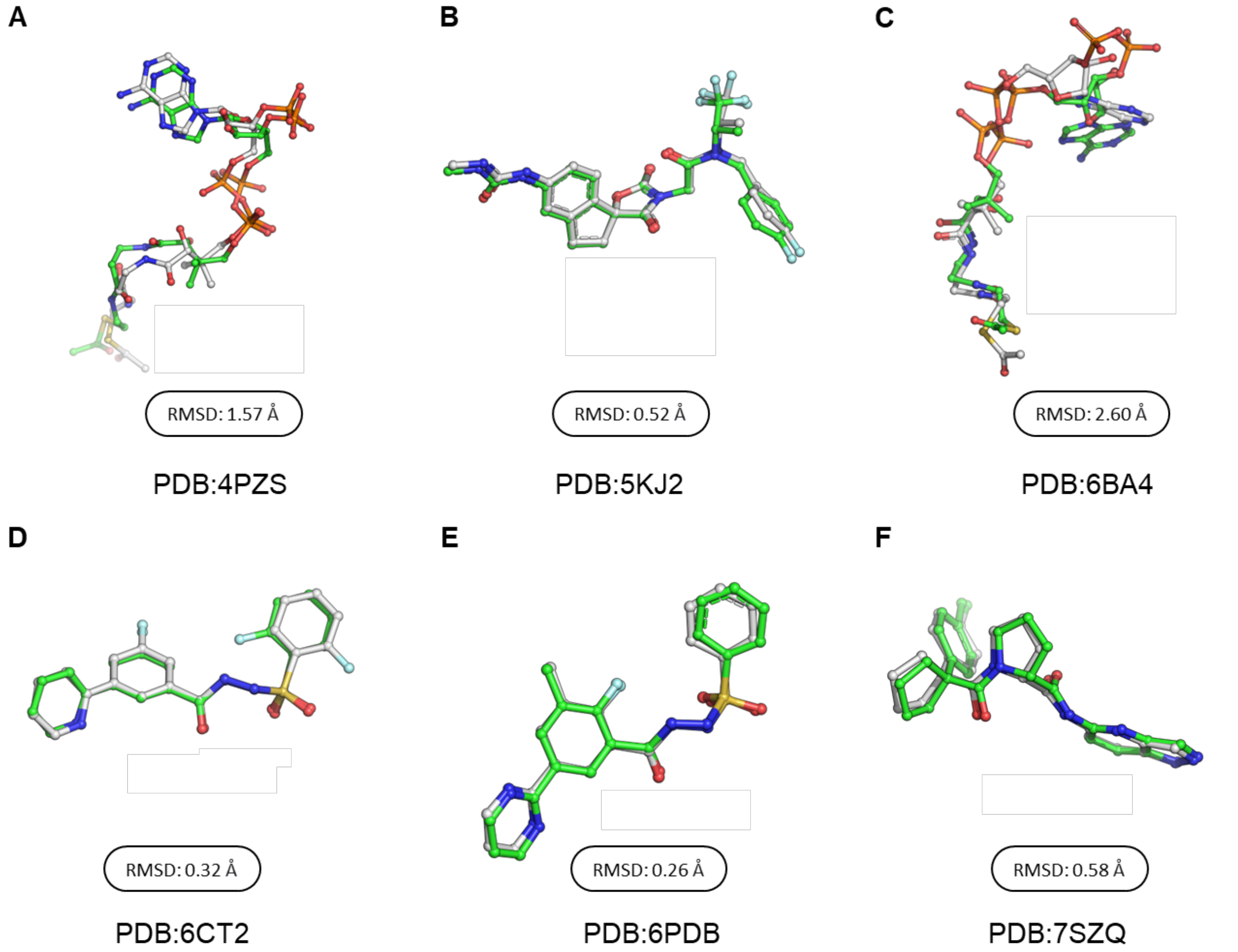

Figure S7. Binding poses of KAT inhibitors or co-substrate Ac-CoA obtained from docking (green), compared to experimentally determined KAT-inhibitor/co-substrate complexes structures from the PDB (white). The docking parameters used for simulating the interactions of the obtained hit compounds were previously setup on representative PDB structures. (A) Binding pose of Ac-CoA in complex with KAT3B obtained from docking compared to the co-crystal structure (PDB ID: 4PZS); (B) Binding pose of A-485 in complex with KAT3B obtained from docking compared to the co-crystal structure (PDB ID: 5KJ2); (C) Binding pose of Ac-CoA in complex with MYST acetyltransferase obtained from docking compared to the co-crystal structure (PDB ID: 6BA4); (D) Binding pose of WM-1119 in complex with MYST acetyltransferase obtained from docking compared to the co-crystal structure (PDB ID: 6CT2); (E) Binding pose of inhibitor 80 in complex with MYST acetyltransferase obtained from docking compared to the co-crystal structure (PDB ID: 6PDB); (F) Binding pose of azaindazole inhibitor ETL in complex with KAT3B obtained from docking compared to the co-crystal structure (PDB ID: 7SZQ).

Analysis of C646 binding mode to KAT3B.

The docked inhibitor C646 is predicted to assume a "V-shaped" conformation, fitting well in the pocket of KAT3B and mimicking the same interactions of acetyl-CoA co-substrate (PDB Code: 4PZS)[1] (Figure S8A), while showing severe steric clashes in KAT8 (Figure S8B). Specifically, in KAT3B, C646 forms hydrogen bonds with the following residues: i) Arg1410 via the nitro group of the 2-nitro-4,5-dimethylbenzene moiety; ii) Ser1400/Gln1455 through the carbonyl oxygen atom of the 5-pyrazolone moiety; iii) Asp1399 via the nitrogen atom at 2-position of the 5-pyrazolone ring; iv) Gln1455/Lys1456 through the carboxyl group of the benzoic acid moiety. Additional important stacking interactions involve His1451 and Trp1466 with the benzoic acid and nitrobenzene moieties, respectively (Figure S8A).

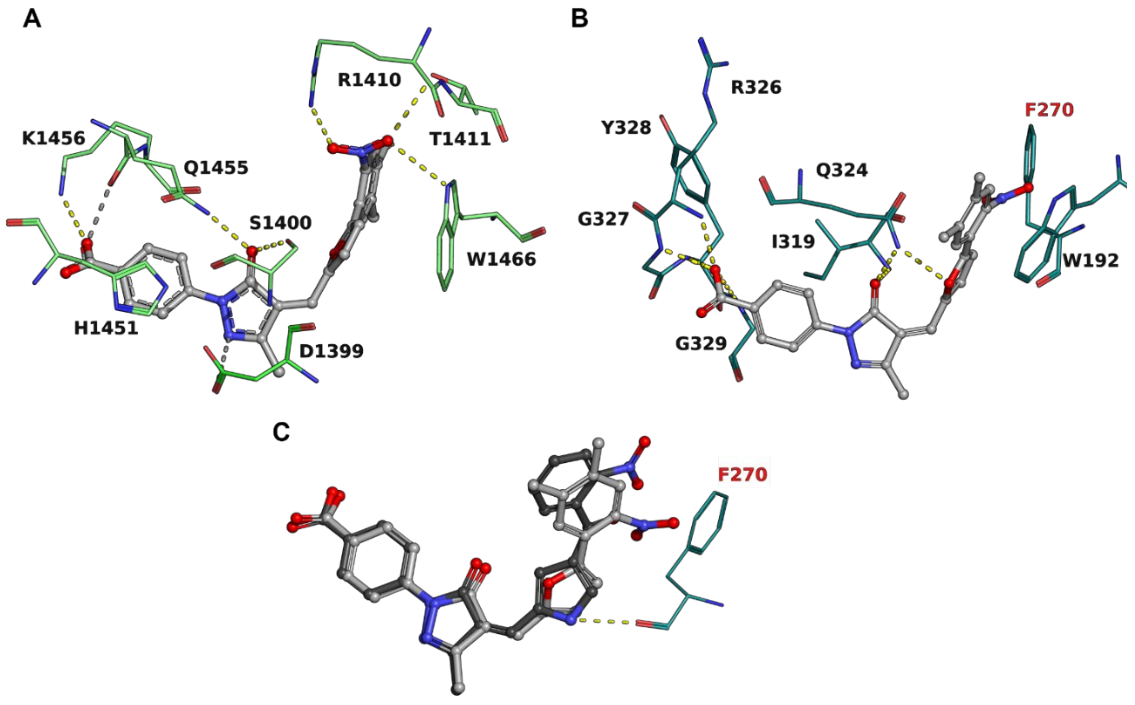

Figure S8. Molecular docking of C646 in the active sites of KAT3B (PDB ID: 5KJ2) and KAT8 (PDB ID: 6PDB). (A) C646 (light grey) docked into the active site of KAT3B. (B) C646 (light grey) docked in the active site of KAT8. (C) Structural alignment between C646 (light grey) and compound **2** (dark grey), showing the hydrogen bond formed between the NH group of the pyrrole ring of **2** and Phe270. C646 nitro group clashes with KAT8 Phe270, while **2**, due to the presence of the carbonyl carbon between the pyrrole and nitrobenzene rings, does not. Hydrogen bonds are shown as yellow dashed lines. Residues of the biding pocket involved in the interactions are shown. Clashing residues are labelled in red.

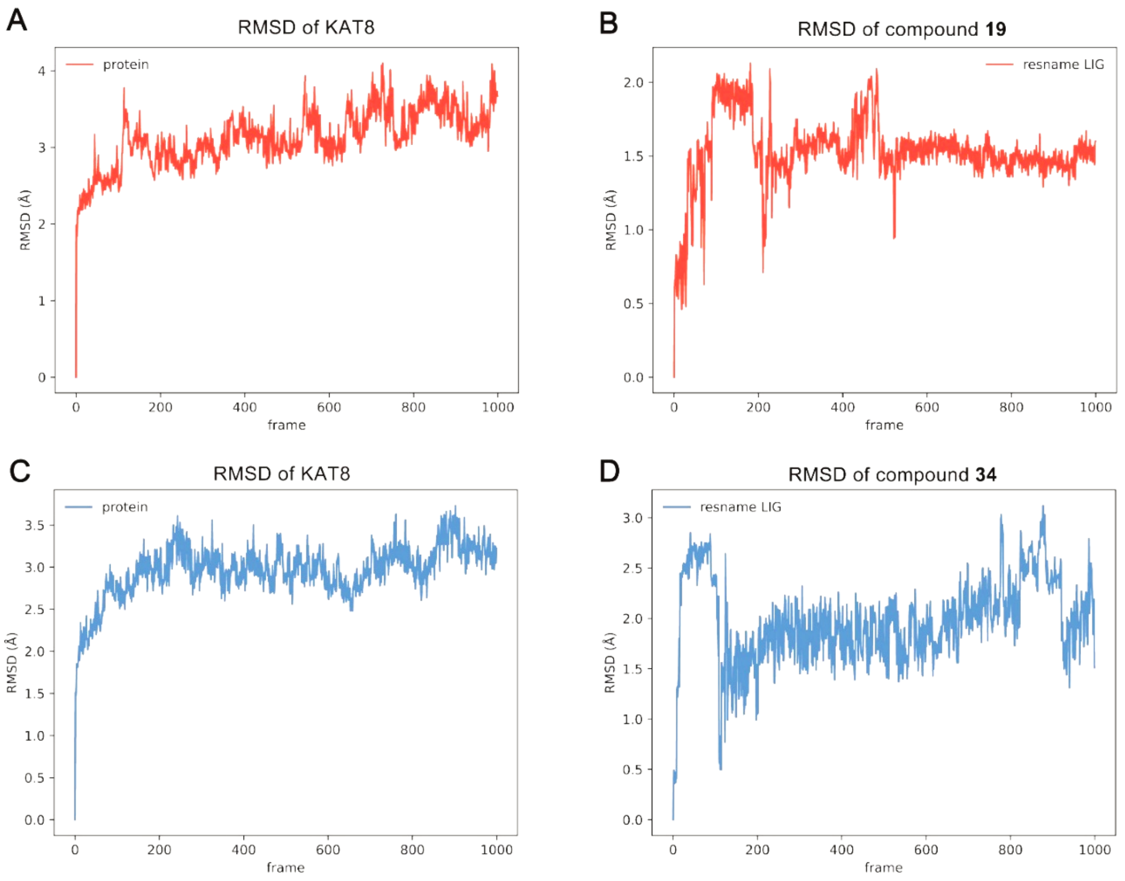

Figure S9. RMSD plots of the MD simulation (100 ns) of compounds **19** and **34**. A) RMSD Plot of KAT8 in complex with **19**. B) RMSD Plot of **19**. C) RMSD Plot of KAT8 in complex with **34**. D) RMSD Plot of **34**.

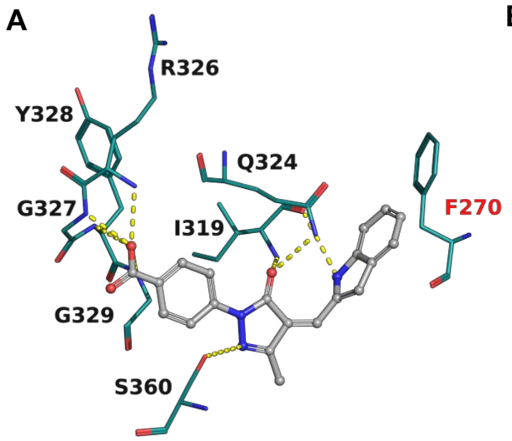

Figure S10. Predicted binding mode of compounds **26** and **27** to KAT8 (PDB ID: 6PDB). Compounds **26** (A) and **27** (B) docked in the active site of KAT8. Hydrogen bonds are shown as yellow dashed lines. Residues of the binding pocket involved in the interactions are shown. Clashing residues are labelled in red.

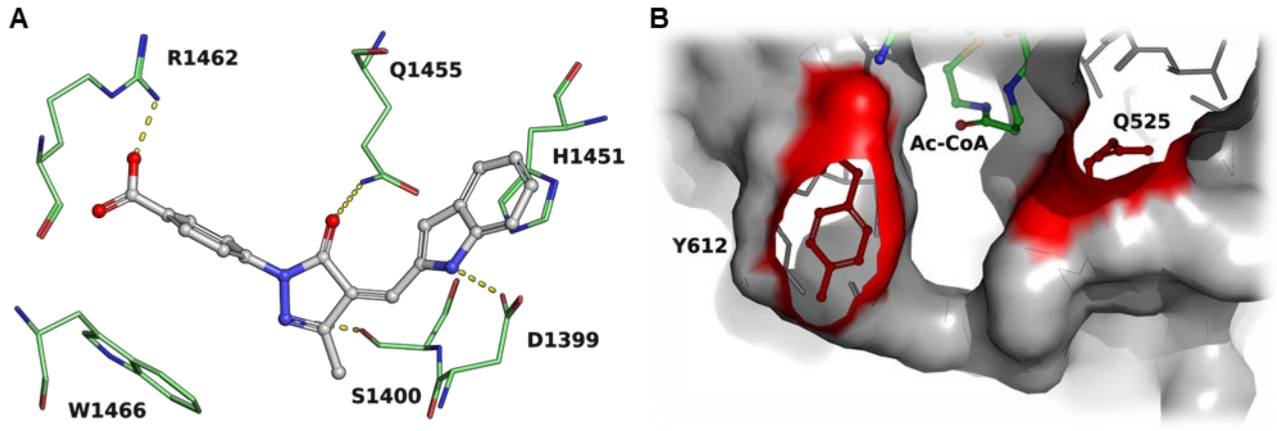

Figure S11. Predicted binding mode of compound **26** to KAT3B (PDB ID: 5KJ2) and focus on KAT2B active site (PDB ID: 4NSQ). (A) Compound **26** (white) docked in the active site of KAT3B. (B) KAT2B with the "disturbing" Tyr612 and Gln525 highlighted in red. Hydrogen bonds are shown as yellow dashed lines. Residues of the biding pockets involved in the interactions are shown.

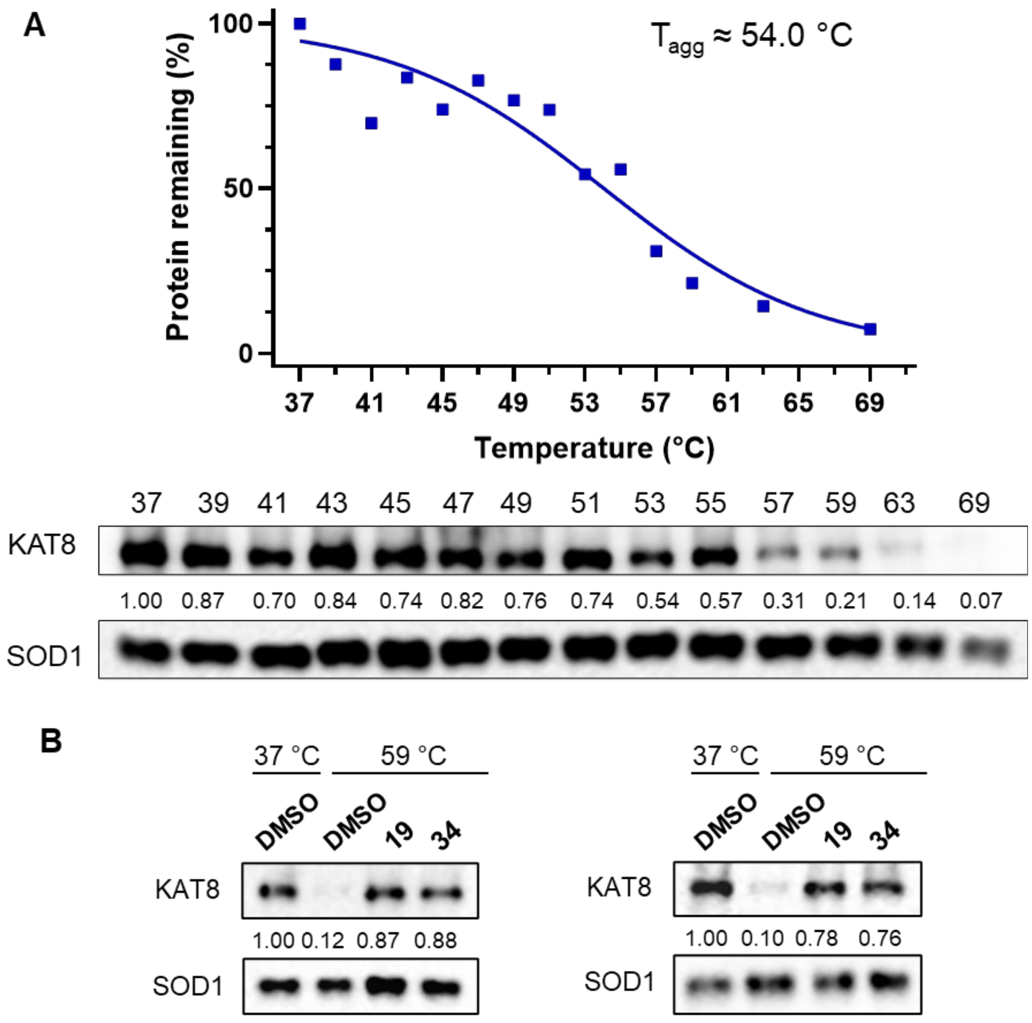

Figure S12. (A) KAT8 aggregation temperature (T_{agg}) calculated by CETSA melt curve in intact HT29 cells. The thermostable SOD1 was used as loading control. The relative protein levels are expressed as percentage of KAT8 levels at 37 °C and normalized to SOD1. (B) WB employed for the calculation of the target engagement of compounds **19** and **34** at 100 µM tested by CETSA in HT29 cells at 59 °C as shown in Figure 5B. The relative protein levels are expressed as percentage of KAT8 levels at 37 °C and normalized to SOD1. Control (DMSO) consists of 0.5% (*v/v*) DMSO-treated cells.

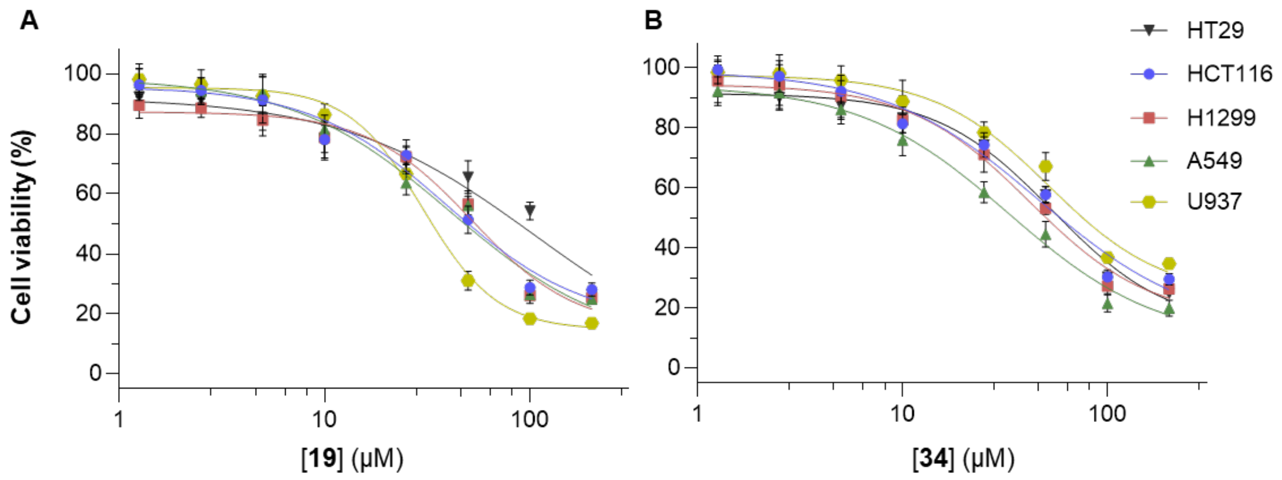

Figure S13. Antiproliferative activities of KAT8i **19** (A) and **34** (B) tested at 1.25, 2.5, 5, 10, 25, 50, 100, and 200 µM in HT29, HCT116, H1299, A549, and U937 cells for 72 h. The control (0 µM) consists of 0.5% (*v/v*) DMSO-treated cells.

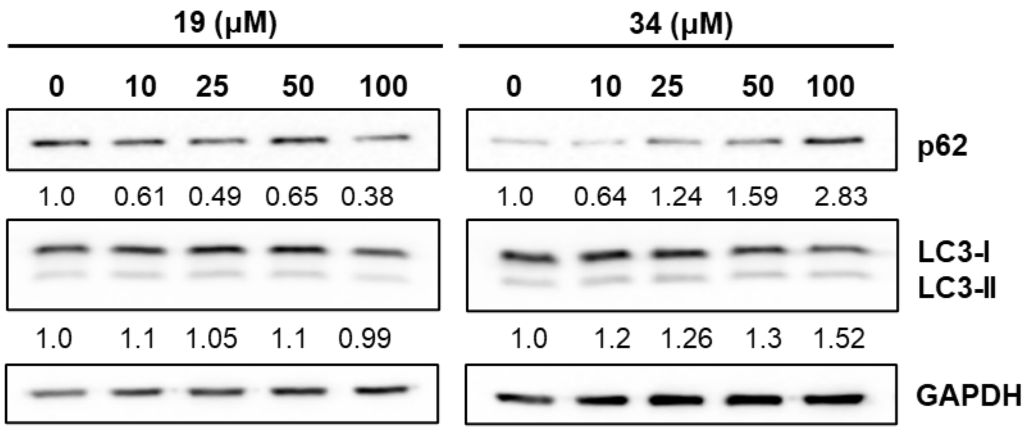

Figure S14. WB analysis of autophagic marker expression levels in HeLa cells exposed to increasing doses of compounds **19** and **34** for 48 h. HeLa cells were treated with increasing concentrations of KAT8i **19** and **34** for 48 h. Cell lysates were collected to assess LC3I/II conversion and SQSTM1/p62 levels by immunoblotting. GAPDH was used as loading and transferring control. Numbers report the densitometric values of band intensity, obtained by the densitometric analysis of the immunoblots. Values of LC3-II/-I ratio and of p62 were normalized to GAPDH as loading control and are reported as fold change (FC) over the control value.

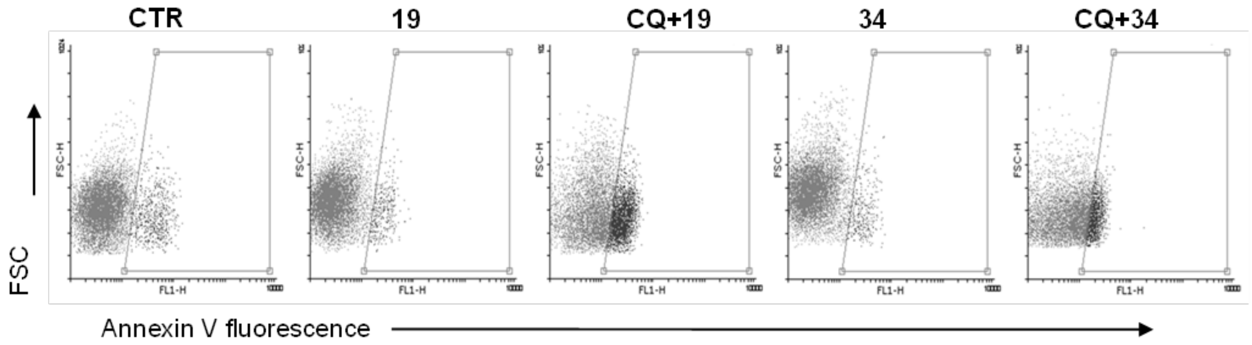

Figure S15. Representative dot plot of Annexin V-FITC fluorescence upon treatment of HCT116 cells with KAT8i **19** and **34** alone (100 μM) or in combination with autophagy inhibitor CQ (10 μM) for 72 h. Boxed areas represent Annexin V-positive cells.

Caption for Supplementary Movies 1-2 (separate files).

Supplementary Movies 1-2. MD simulations (100 ns) of **19** and **34** in complex with KAT8. The movies represent the trajectory of **19** (Supplementary Movie 1) and **32** (Supplementary Movie 2) within the active site of KAT8. A frame was stored from the MD simulation each 0.1 ns, for a total of 1000 frames. Each second in the movie represent the smoothing of 30 frames. The hydrogen bonds forming during the simulation are represented as blue dashed lines.

References

1. Maksimoska, J.; Segura-Pena, D.; Cole, P. A.; Marmorstein, R., Structure of the p300 histone acetyltransferase bound to acetyl-coenzyme A and its analogues. *Biochemistry* **2014**, 53(21), 3415-3422.
